# Nucleoside diphosphate kinase A (NME1) catalyses its own oligophosphorylation

**DOI:** 10.1038/s41557-025-01915-8

**Published:** 2025-08-20

**Authors:** Arif Celik, Felix Schöpf, Christian E. Stieger, Sarah Lampe, Björn Hanf, Jeremy A. M. Morgan, Max Ruwolt, Fan Liu, Christian P. R. Hackenberger, Daniel Roderer, Dorothea Fiedler

**Affiliations:** 1 Leibniz-Forschungsinstitut für Molekulare Pharmakologie (FMP), Berlin, Germany; 2 Institut für Chemie, Humboldt-Universität zu Berlin, Berlin, Germany

**Keywords:** Phosphorylation, Cryoelectron microscopy, Proteomic analysis

## Abstract

Protein phosphorylation is a central signalling mechanism in eukaryotic cells. The scope of this post-translational modification includes protein pyro- and polyphosphorylation. Here we report the discovery of another mode of phosphorylation: protein oligophosphorylation. Using site-specifically phosphorylated and pyrophosphorylated nucleoside diphosphate kinase A (NME1), the effects of these modifications on enzyme activity were investigated. Phosphorylation, and more so pyrophosphorylation, on Thr94 reduced the nucleoside diphosphate kinase activity. Nevertheless, both phosphoprotein and pyrophosphoprotein catalysed their own oligophosphorylation—up to the formation of a hexaphosphate chain—using ATP as a cofactor. Oligophosphorylation was critically dependent on the catalytic histidine residue His118, and cryogenic electron microscopy analysis of the modified proteins suggests an intramolecular phosphoryl transfer mechanism. Oligophosphorylation of NME1 in biochemical samples, and in cell lysates, was further confirmed using mass spectrometry, and was found to promote a new set of protein interactions. Our results highlight the complex nature of phosphoregulation, and the methods described here provide the opportunity to investigate the impact of this unusual modification in the future.

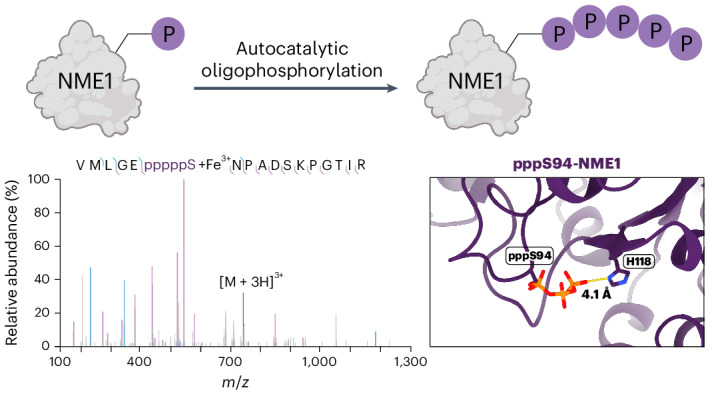

## Abstract (web summary)

Our understanding of how post-translational modification—protein phosphorylation—impacts the complexity of eukaryotic signalling pathways is continuously expanding. Now, protein oligophosphorylation has been characterized as an additional phosphorylation mode. Structural and mass spectrometry methods revealed that NME1 catalysed its own oligophosphorylation, leading to altered protein–protein interactions.

## Main

Phosphorylation of proteins has a central role in cellular signalling and contributes to the regulation of a plethora of processes^1,2^. Following the characterization of serine, threonine and tyrosine phosphorylation in mammalian cells, a wide range of methods for the interrogation of protein phosphorylation have been developed. For example, bottom-up phosphoproteomic analyses enable the annotation of thousands of phosphorylation sites in a single experiment^3,4^. Highly selective pan-specific antibodies are available for the detection of phosphotyrosine (pY), and phosphoserine (pS) and phosphothreonine (pT) are readily detected within specific sequences^5–7^. Furthermore, the Protein Data Bank (PDB) contains many structures of proteins phosphorylated on serine, threonine and tyrosine, which have contributed notably to our understanding of phosphoregulation.

More recently, the investigation of the mammalian phosphoproteome has been expanded to include non-canonical phosphorylation (on histidine, arginine, cysteine, aspartate, glutamate and lysine side chains)^8–12^, but the corresponding analytical tools are lagging behind. Useful antibodies for the detection of phosphohistidine (pHis) and phosphoarginine (pR) have been reported^13–15^. But owing to the higher lability of these phosphorylation sites, their characterization using mass-spectrometric and/or structural methods has been challenging^8,12,16^.

Another emerging mode of non-canonical phosphorylation is protein pyrophosphorylation, in which a pS or pT residue is further phosphorylated by inositol pyrophosphate messengers to yield a diphosphorylated (also termed pyrophosphorylated) side chain^17,18^. Moreover, the complexity of protein phosphorylation is further increased with the discovery of protein polyphosphorylation. This modification entails the attachment of inorganic polyphosphate (polyP) chains to PASK (polyacidic, serine- and lysine-rich) domain-containing proteins^19^. The exact nature of the polyP chain attachment is still under debate, and it is not clear if the polyP chain is covalently linked to lysine side chains via a phosphoramidate bond or if the attachment is based on very strong electrostatic interactions with the target sequences^20–22^. Because structural and/or mass spectrometry (MS) analyses of this intriguing modification have not been reported to date, it remains to be seen if one or both of these hypotheses can be validated.

In the case of protein pyrophosphorylation, a recently developed MS approach identified around 150 pyrophosphorylation sites, but structural characterization has been lacking^23^. Although most pyrophosphorylation sites reside in disordered, serine-rich polyacidic stretches^23^, several pyrophosphorylation sites did not fit that pattern and localized to proline-directed consensus sequences within structurally resolved regions. Among these latter examples were a number of kinases, including glycogen synthase kinase-3α/β (GSK3α/β), *N*-acetyl-d-glucosamine kinase (NAGK) and nucleoside diphosphate kinase A/B (NME1/2).

Nucleoside diphosphate kinase A (NME1) is a multifunctional enzyme that was found to be pyrophosphorylated on Thr94 in human cells^23^. A major role of NME1 is the synthesis of nucleoside triphosphates, other than ATP. NME1 catalyses this process by transferring the γ-phosphoryl group from ATP to a nucleoside diphosphate (NDP) via an active-site phosphohistidine intermediate^24^. Moreover, NME1 was recognized as a serine/threonine kinase, and it phosphorylates the kinase suppressor of Ras1 (KSR1)^25^. NME1 has also been proposed to be a mammalian histidine kinase^8^ because it can phosphorylate histidine residues of protein substrates, such as ATP-citrate lyase and succinate thiokinase, in vitro^8,26–28^.

**Fig. 1:**
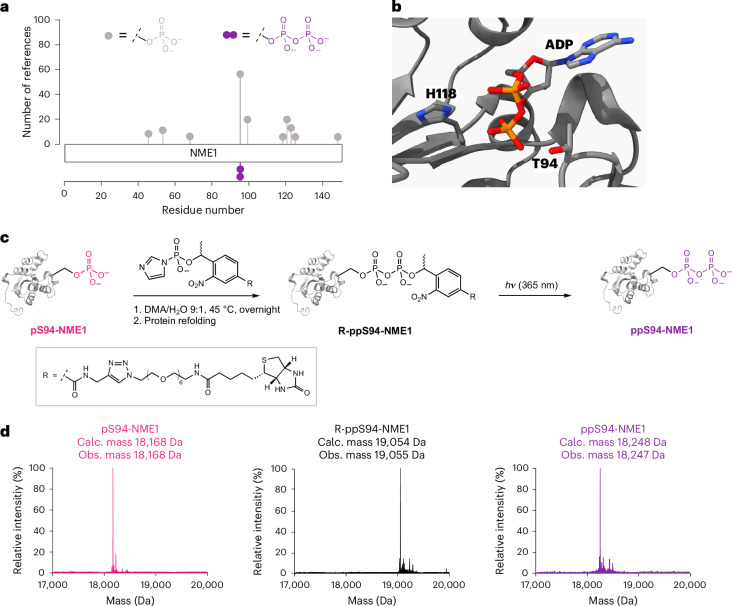
(Pyro)phosphorylation sites of NME1 and generation of pS94-NME1 and ppS94-NME1. **a**, Phosphorylation sites on NME1 reported in PhosphoSitePlus^33^ (grey) and previously discovered pyrophosphorylation site at Thr94 (purple)^23^. Accessed 29 April 2025. **b**, Structure of NME1 co-crystallized with ADP, highlighting the location of Thr94, His118 and ADP (PDB: 7ZLW)^39^. Carbon atoms are shown in grey, oxygen atoms in red, nitrogen atoms in blue, and phosphorus atoms in orange. **c**, Chemical phosphorylation of pS94-NME1 to provide pyrophosphoprotein ppS94-NME1. pS94-NME1 is derivatized by using biotin-PEG_6_-Tz-NPE-P-imidazolide for the selective modification of the phosphoserine moiety, to yield R-ppS94-NME1. Subsequent irradiation releases the pyrophosphoprotein ppS94-NME1. Protein structure, PDB: 7ZLW. **d**, Deconvoluted Q-TOF-MS spectra of the intermediates and products of the reaction sequence in **a**.

Functionally, NME1 has been characterized as a metastasis suppressor in both melanoma and breast cancer, where NME1 expression levels directly correlate with its ability to suppress cell migration^29,30^. However, phosphorylation at Ser120, Ser122 and Ser125 has been linked to tumour progression, highlighting the complex role of NME1 and its regulation via phosphorylation^31,32^. Several additional phosphorylation sites on NME1 have been annotated in high-throughput studies (Fig. 1a), yet the functional relevance of the most frequently detected phosphorylation site at Thr94, which is in close proximity to the ATP binding site, has not been fully investigated^33^^,^^34^ (Fig. 1b).

Thr94 is also the site at which NME1 is pyrophosphorylated, and we now report the synthesis and characterization of stoichiometrically phosphorylated and pyrophosphorylated NME1. Phosphorylation reduced NDP kinase activity, and pyrophosphorylation led to complete inactivation of the enzyme. Unexpectedly, MS analysis and cryogenic electron microscopy (cryo-EM) revealed an ATP-dependent, autocatalytic formation of an oligophosphate chain on Thr94 in vitro, which only required the initial installation of a monophosphate group by cyclin-dependent kinase 1 (CDK1). Oligophosphorylated NME1 was subsequently detected in cell lysates, and this unprecedented post-translational modification appears to mediate a wide range of protein interactions. Our results highlight protein oligophosphorylation as yet another mode of phosphoregulation, and the methods described here will pave the way to investigate the occurrence and the function of this intriguing modification in the future.

## Results

### Generation of phosphorylated and pyrophosphorylated NME1

**Extended Data Fig. 1:**
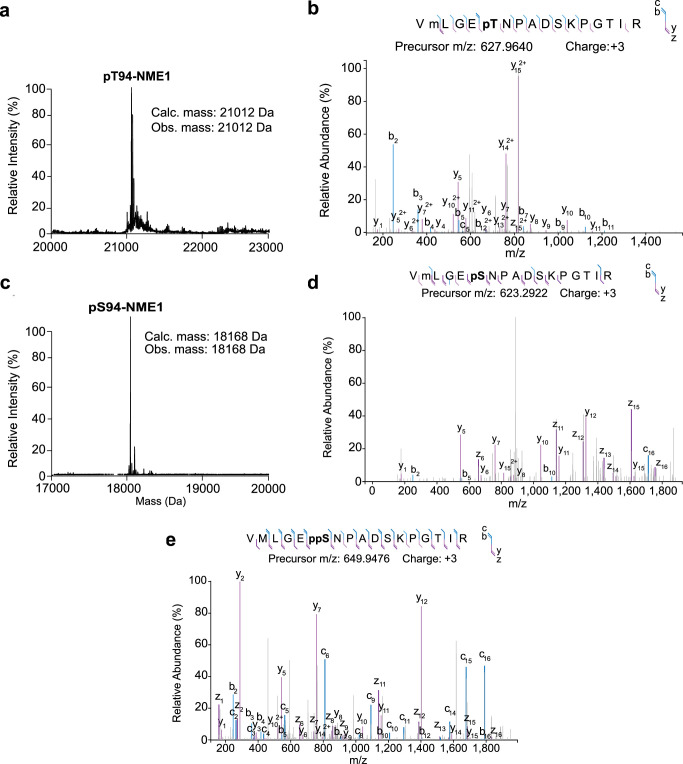
Characterization of recombinantly expressed pT94-NME1, pS94-NME1, and ppS94-NME1. **a)** Q-TOF-MS measurement of pT94-NME1. **b)** MS/MS analysis measured on an Orbitrap Fusion, localizing phosphorylation on T94. **c)** Q-TOF-MS measurement of pS94-NME1. **d)** MS/MS analysis measured on an Orbitrap Fusion, localizing phosphorylation on S94. **e)** MS/MS analysis measured on an Orbitrap Fusion, localizing pyrophosphorylation on S94. pT94-NME1 has a higher molecular mass because it was expressed in a pET151a vector that contains an intact TEV-cleavage site, V5 Epitope tag, and TOPO cloning vector sequence, whereas pS94-NME1 was expressed in a pNIC28-Bsa vector, which does not have a TEV-cleavage site.

To investigate how phosphorylation and pyrophosphorylation at Thr94 may influence the properties of NME1, a site-specifically and stoichiometrically modified protein is required. This was accomplished by using a previously reported method that combines amber codon suppression and a chemoselective reaction using a photolabile P-imidazolide reagent^35,36^. Initial attempts focused on the expression of pT94-NME1 in *Escherichia coli*^37^. Although the desired phosphoprotein pT94-NME1 was obtained in high purity, the yields were very low (0.05 mg l^−1^, compared with 33 mg l^−1^ for wild-type NME1; Supplementary Figs. 1 and 2, and Extended Data Fig. 1a,b). Because a decent quantity of phosphoprotein is required for subsequent conversion to the pyrophosphoprotein, we decided to incorporate pS as a surrogate for pT^38^. This strategy proved effective, yielding high amounts of pS94-NME1 (0.75 mg l^−1^) with the expected mass of 18,168 Da (Supplementary Fig. 3 and Extended Data Fig. 1c,d). pS94-NME1 was then treated with biotin-polyethylenglycol-6-triazole-nitrophenylethyl-P-imidazolide (biotin-PEG_6_-Tz-NPE-P-imidazolide) overnight at 45 °C in a solvent mixture of dimethylacetamide (DMA)/H_2_O (9:1 v/v) (Fig. 1c). Following protein refolding, the formation of the derivatized protein, R-ppS94-NME1, was confirmed using quadrupole time-of-flight mass spectrometry (Q-TOF-MS). After exposure to 365-nm light, the pyrophosphoprotein was released, resulting in the isolation of ppS94-NME1. Compared to pS94-NME1, ppS94-NME1 displayed a mass increase of 80 Da, confirming the addition of a phosphoryl group (Fig. 1d and Extended Data Fig. 1e).

To verify the effectiveness of the refolding step, the derivatization/refolding conditions were also applied to wild-type NME1. The refolded protein displayed activity levels comparable to those of the reference protein (Supplementary Fig. 4), indicating successful restoration of the protein structure, further confirmed by circular dichroism spectroscopy (Supplementary Fig. 5).

### Pyrophosphorylation reduces the NDP kinase activity of NME1

**Fig. 2:**
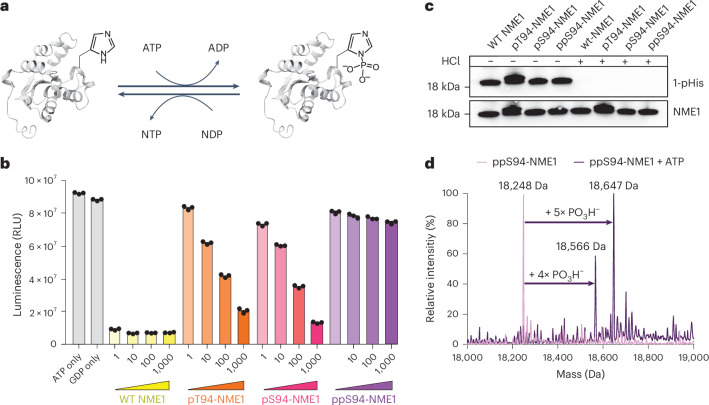
Phosphorylation and pyrophosphorylation of NME1 reduce the NDP kinase activity. **a**, NME1 catalyses phosphoryl transfer via an active-site phosphohistidine intermediate. (PDB: 7ZLW). **b**, NDP kinase activity was measured utilizing GDP as a substrate, at 37 °C for 1 h in 50 mM Tris–HCl (pH 8.0), 150 mM NaCl, 10 mM MgCl_2_, 900 μM GDP and 100 µM ATP. Concentrations of NME1 are in nM (1–1,000). Data are presented as mean ± s.e.m. of three technical replicates. RLU, relative light units; WT, wild-type. **c**, Autophosphorylation reactions of NME1 were carried out at 37 °C for 1 h in 50 mM Tris–HCl, 10 mM MgCl_2_, 1 mM DTT and 1 mM ATP. Hydrolysis was induced by incubating the autophosphorylated samples in 1 M HCl for 1 h at 37 °C. Subsequently, all samples were analysed by western blot using an anti-NME1 and a pan-specific 1-pHis antibody^14^. pT94-NME1 has a higher molecular mass due to an additional TEV-cleavage site between the His_6_-tag and the NME1 sequence. Representative images from *n* = 3 independent experiments are shown. **d**, ppS94-NME1 was incubated at 37 °C for 1 h in 50 mM Tris–HCl, 10 mM MgCl_2_, 1 mM DTT and 1 mM ATP. Deconvoluted Q-TOF-MS spectra of ppS94-NME1 and ATP-treated ppS94-NME1 indicate additional phosphorylation. Source data

With the different (pyro)phosphoprotein variants of NME1 in hand, we next evaluated how these modifications impacted the NDP kinase activity of NME1, using a standard kinase assay with GDP or TDP as substrates and monitoring the consumption of ATP (Fig. 2a). With GDP as a substrate, wild-type NME1 (at an enzyme concentration of 1 nM) exhibited high enzymatic activity. By contrast, pT94-NME1 showed no detectable activity at the same enzyme concentration. Even when the enzyme concentration was increased, the overall activity of pT94-NME1 was approximately 100-fold decreased compared with wild-type NME1, consistent with previous reports of a T94D mutant, which may be regarded as a phosphomimetic^34,39,40^. The activity profile of pS94-NME1 was very similar to that of pT94-NME1, reduced approximately 100-fold compared with wild-type NME1 (Fig. 2b), suggesting that the pS94 mutation is a suitable substitute for pT94.

**Extended Data Fig. 2:**
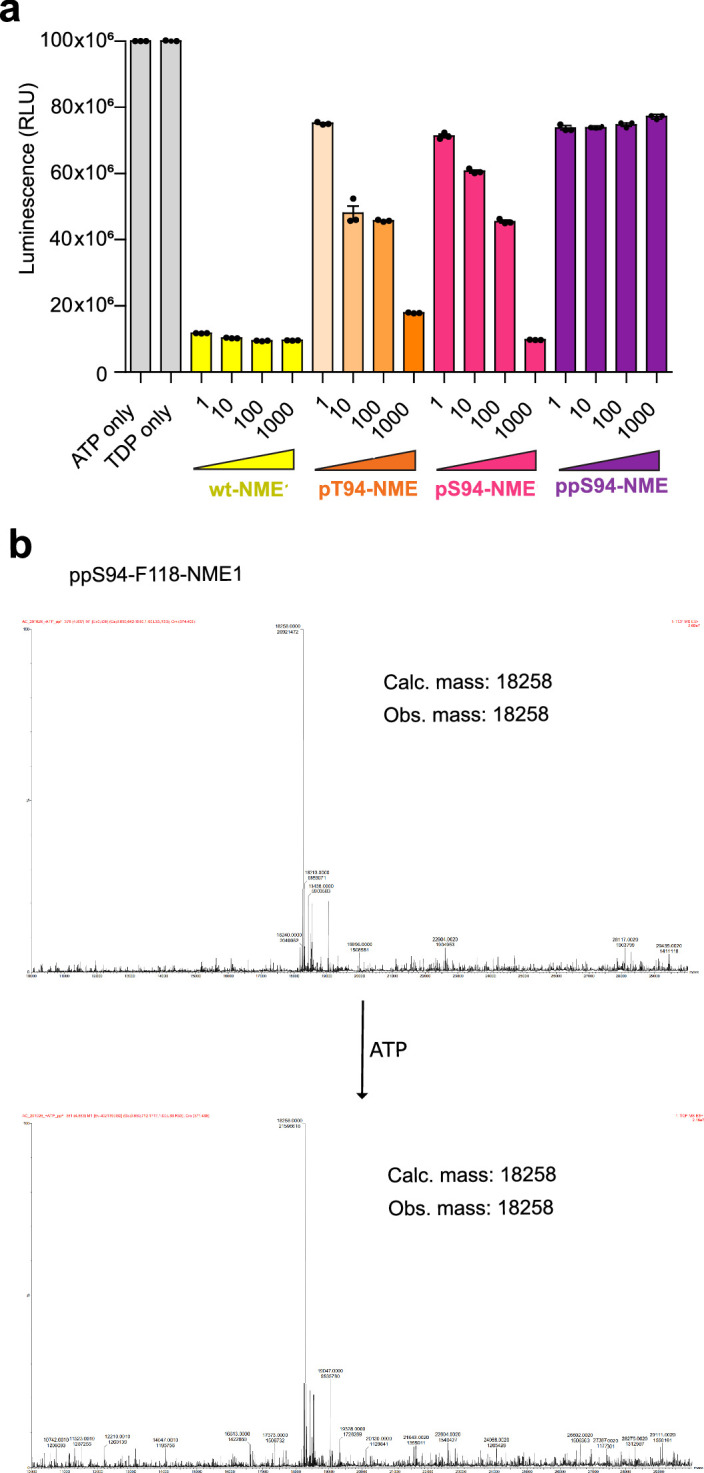
NME1 NDP kinase activity and diminished autophosphorylation by H118 mutation. **a)** Phosphorylation and pyrophosphorylation of NME1 reduce the nucleoside diphosphate (NDP) kinase activity. NDP kinase activity assay utilizing TDP as a substrate was measured at 37 °C for 1 h in 50 mM Tris–HCl (pH 8.0), 150 mM NaCl, 10 mM MgCl_2_, 900 µM TDP, and 100 µM ATP. Concentrations of NME1 (1–1000) are shown in nM. **b)** ATP-treatment of ppS94-F118-NME1 analysed by Q-TOF-MS. Autophosphorylation experiments were performed at 37 °C for 1 h in 50 mM Tris–HCl, 10 mM MgCl2, 1 mM DTT, and 1 mM ATP. Source data

The assay for ppS94-NME1 revealed that the pyrophosphoprotein exhibited almost no NDP kinase activity, even at a protein concentration of 1 µM. A very similar trend for the different (pyro)phosphoproteins was observed when TDP was used as a substrate: wild-type NME1 demonstrated robust activity towards TDP, whereas the activity of pS94-NME1 was reduced ∼100-fold; ppS94-NME1 was essentially inactive (Extended Data Fig. 2a).

Given that Thr94 is adjacent to the nucleotide binding site, phosphorylation or pyrophosphorylation at this residue may make substrate access more difficult, due to electrostatic repulsion. The lowered NDP kinase activity of NME1 may therefore also correlate with reduced phosphorylation of the catalytic histidine, His118. This residue is critical for the phosphoryl transfer mechanism of NME1, as a NME1(H118F) mutant showed no activity in an NDP kinase assay (Supplementary Fig. 6). However, for all (pyro)phospho-NME1 variants, the presence of the acid-labile 1-pHis118 intermediate was detected at similar levels, using a pan-specific 1-pHis antibody (Fig. 2c). The presence of acid-labile phosphorylation following ATP treatment was further confirmed by Q-TOF-MS for both wild-type NME1 and pS94-NME1 (Supplementary Figs. 7 and 8).

Interestingly, when ppS94-NME1 was incubated with ATP, an addition of four or five phosphoryl groups (+360 and +400 Da) became apparent (Fig. 2d). This ‘hyperphosphorylation’ was dependent on the catalytic His118 residue because the kinase-inactive mutant ppS94-NME1(H118F) did not undergo this modification (Extended Data Fig. 2b).

Overall, the NDP kinase activity of NME1 is critically regulated by phosphorylation and pyrophosphorylation at Thr94/Ser94. Phosphorylation greatly reduced the NDP kinase activity, and pyrophosphorylation virtually inactivated the NDP kinase activity of NME1. Unexpectedly, the pyrophosphoprotein underwent ‘hyperphosphorylation’, prompting us to further investigate this process.

### NME1 can undergo auto-oligophosphorylation

**Fig. 3:**
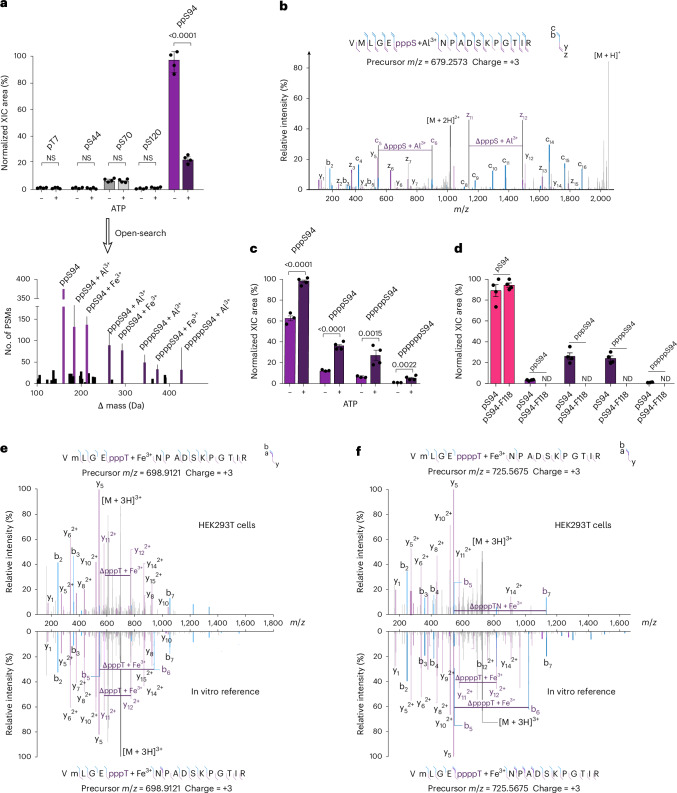
Characterization of oligophosphorylated NME1 using MS. **a**, Relative quantification of ATP-treated ppS94-NME1 showed no increase of known phosphorylation sites but a decrease of pyrophosphorylation, while a substantial number of oligophosphorylated PSMs revealed modification corresponding to oligophosphorylated NME1 peptides. The samples were processed in four technical replicates. *P* values were determined by an unpaired two-sided *t*-test: *P* < 0.0001 for ppS94; NS, not significant. **b**, Unambiguous localization of triphosphorylation to Ser94 by EThcD MS/MS. **c**, Relative quantification of different oligophosphorylation states before and after ATP treatment of ppS94-NME1. Assay was performed by incubating 1 µM ppS94-NME1 in 20 mM Tris–HCl (pH 8.0), 1 mM ATP, 5 mM MgCl_2_ and 1 mM DTT. *P* values were determined by an unpaired two-sided *t*-test: *P* < 0.0001 for pppS94, *P* < 0.0001 for ppppS94, *P* = 0.0015 for pppppS94, *P* = 0.0022 for ppppppS94. **d**, Relative quantification of different oligophosphorylation states in recombinantly expressed pS94-NME1 and pS94-NME1(H118F). The relative quantification was determined by the FreeStyle 1.7 software, integrating the MS1 intensities of Fe^3+^-bound oligophosphorylated peptide masses relative to the integrated area of the full chromatogram. Data are presented as mean ± s.e.m. of four technical replicates. ND, not detected. **e**,**f**, Comparison of stepped-HCD MS/MS spectra obtained from triphosphorylated (**e**) and tetraphosphorylated (**f**) NME1 peptide in enriched HEK293T cell lysate and recombinantly expressed pT94-NME1 shows excellent sequence coverage while preserving the modification. Source data

To understand where the addition of phosphoryl groups occurred when ppS94-NME1 was incubated with ATP, the reaction products were digested with trypsin and analysed by tandem mass spectrometry (MS/MS) and label-free quantification (LFQ). Surprisingly, no additional phosphorylation sites could be identified, nor did any of the known phosphorylation sites show a substantial increase following ATP treatment. The only notable change in the MS1 spectra was a large reduction of the signal for the pyrophosphopeptide upon exposure to ATP (Fig. 3a). Because the Q-TOF-MS clearly indicated the addition of several phosphoryl groups, we reasoned that an extension of the pyrophosphate to an oligophosphate chain could explain the data.

To identify putative oligophosphorylation, the open-search feature integrated into MSFragger was leveraged to facilitate an unbiased exploration of potential modifications^41,42^. Alongside a substantial number of pyrophosphorylated peptide-spectrum-matches (PSMs; +160 Da, all localized to the sequence containing Ser94), more than 100 spectra revealed a modification corresponding to the mass of pyrophosphorylation with the additional presence of Al^3+^ or Fe^3+^ ions, resulting in respective mass shifts of +184 Da or +213 Da (Fig. 3a). Remarkably, peptides that featured three to five phosphoryl groups on Ser94 were also identified. Further validation through manual inspection of the top-scoring spectrum matches confirmed the precise localization of these modifications to Ser94 (Fig. 3b).

**Extended Data Fig. 3:**
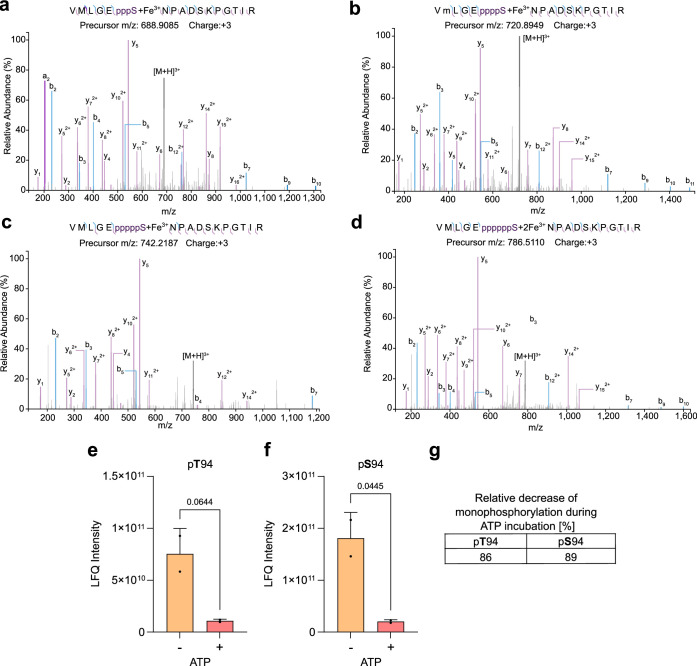
Analysis of oligophosphorylated S94-NME1 by LC–MS/MS and evaluation of the effect of the amino acid residue (pThr vs. pSer) upon ATP incubation. **a-d)** Analysis of ATP-treated ppS94-NME1 by stepped-HCD MS/MS measured on Orbitrap Fusion localizing **a)** triphosphorylation, **b)** tetraphosphorylation, **c)** pentaphosphorylation, and **d)** hexaphosphorylation on S94-NME1. **e-g)** Evaluation of the amino acid residue (Thr *versus* Ser) effect on the oligophosphorylation during ATP incubation. The decrease in monophosphorylation indicates the oligophosphorylation of pT94/pS94. The assay was performed using a mixture of pT94-NME1 and pS94-NME1, which was incubated with or without 1 mM ATP at 37 °C and 300 rpm for 1 h in 20 mM Tris–HCl (pH 8.0), 5 mM MgCl2, and 1 mM DTT. After a tryptic digest, the samples were measured on an Orbitrap Lumos using HCD fragmentation. **e)** LFQ data of pT94-NME1, and **f)** pS94-NME1. Data is represented as mean ± SD of two biological duplicates. **g)** Decrease of monophosphorylation during ATP incubation calculated using the mean values. See Supplementary Information for experimental procedures. Source data

Although this method enabled the unambiguous identification of oligophosphorylation on Ser94, many spectra exhibited poor fragment intensity. Instead, instances of electron transfer with no dissociation were observed, in which the peptide ion loses a charge without undergoing subsequent fragmentation (Supplementary Fig. 9). This phenomenon was more pronounced in peptide ions containing an iron ion and could be attributed to the high electron affinity of Fe^3+^ (ref. ^43^). Therefore, fragmentation techniques were screened, and higher-energy C-trap dissociation (HCD) with low normalized collision energy (NCE) values (NCE = 20–25) was identified as a more reliable method to detect oligophosphorylated peptides^44^^,^^45^ (Supplementary Fig. 10). As a result of optimization, a stepped-HCD method (NCE = 20–23–26) was chosen and numerous tryptic peptides containing oligophosphate chains—up to hexaphosphate—were successfully identified, accompanied by excellent sequence coverage (Extended Data Fig. 3a–d). Again, the peptides were exclusively observed as Fe^3+^ or Al^3+^ adducts, underscoring the strong chelating properties of oligophosphorylated peptides. Nevertheless, the NDP kinase activities of wild-type, pS94- and ppS94-NME1 were not affected by the presence of these trivalent metals (Supplementary Figs. 11–13).

Using relative quantification, a notable increase across all oligophosphorylation states was observed after ppS94-NME1 was treated with ATP (Fig. 3c). Interestingly, oligophosphorylation was also detected in the ppS94-NME1 sample that had not been ATP treated (Fig. 3c). Because it seemed unlikely that oligophosphorylation could occur during the chemical phosphorylation reaction, we reasoned that oligophosphorylation might have taken place before the derivatization, during the expression of the pS94 mutant in *E. coli*. To validate this hypothesis, pS94-NME1 was freshly expressed and analysed by MS/MS, indeed revealing the autocatalytic formation of a pyrophosphate group at Ser94, and the formation of oligophosphate chains at this position. By contrast, neither wild-type NME1 nor the double mutant pS94-NME1(H118F) showed any higher phosphorylation states (Fig. 3d). These observations suggest an autocatalytic phosphoryl transfer between His118 and prephosphorylated Ser94, to generate the pyro- and oligophosphorylation states. Given that the innate modification site is a threonine residue, we incubated pT94-NME1 with ATP and analysed the sample via MS/MS using stepped-HCD, which clearly confirmed pyro- and oligophosphorylation on threonine (Extended Data Fig. 3e–g and Supplementary Fig. 14).

In summary, MS-based characterization of NME1 uncovered an autocatalytic pyrophosphorylation and oligophosphorylation mechanism in vitro that critically depends on His118. Because prephosphorylation on Thr94 appears to be the only requirement for auto-oligophosphorylation, we wondered whether these higher modes of protein phosphorylation could also be observed in human cells.

### Oligophosphorylation of NME1 occurs in human cell lines

To potentially detect oligophosphorylated NME1 in complex samples, we used an enrichment workflow originally designed for pyrophosphorylated peptides^23^. Briefly, HEK293T cells were lysed, digested and subsequently treated with λ-phosphatase to lower the amounts of monophosphorylated and multiply phosphorylated peptides while leaving oligophosphate chain modifications intact (Supplementary Fig. 15). Following enrichment via sequential ion metal affinity chromatography (SIMAC)^23,46^ and offline fractionation, all peptide fractions were measured using the optimized stepped-HCD method.

Intriguingly, alongside the already known endogenously pyrophosphorylated NME1 tryptic peptide^23^, triphosphorylated and tetraphosphorylated NME1 peptides were identified. Accordingly, ion couplets in the b/y series in the fragmentation spectra for the endogenous peptides (as well as the corresponding in vitro reference spectra) allowed the unambiguous localization of tri- and tetraphosphorylation of NME1 to Thr94, thereby providing direct evidence of endogenous oligophosphorylation (Fig. 3e,f). The measurements also substantiate the reliability of the stepped-HCD method for accurate identification of endogenously oligophosphorylated peptides and will be a good starting point to develop optimized bottom-up oligophosphoproteomic methods in the future.

**Extended Data Fig. 4:**
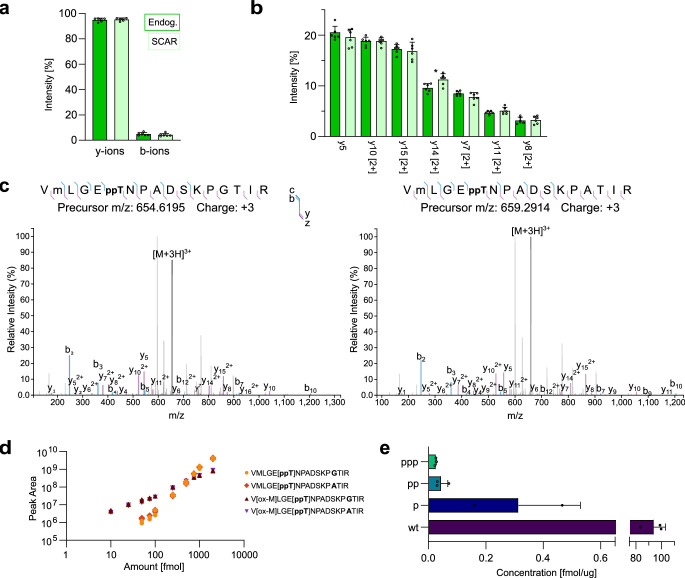
Quantitation of endogenous T94-NME1 phosphorylation states. **a-d)** Validation of the pyrophosphorylated SCAR standard peptide. 2 pmol standard peptides (endogenous and SCAR sequence) were measured on Orbitrap Lumos using stepped HCD. **a)** C- and N-terminal charge retention using y- and b-ion signals (mean ± SD, n = 6 spectra). **b)** Relative intensities of the most abundant y-ion transitions were calculated as the percentage of the total y-ion signal (mean ± SD, n = 6 spectra, t-test indicates no significant differences between endogenous and SCAR signals, except for the y14[2 + ]-transition with P = 0.017 – P < 0.05 is considered significant). **c)** MS/MS spectra of the standard peptides. **d)** Peak areas of a dilution series of a standard peptide mixture (10 fmol, 25 fmol, 50 fmol, 75 fmol, 100 fmol, 250 fmol, 500 fmol, 750 fmol, 1 pmol, and 2 pmol). After synthesis, the peptides were partly present with M-oxidation and partly without oxidation. The samples were measured with stepped HCD (20‒23‒26) on an Orbitrap Lumos instrument. Data is presented as mean ± SD of technical duplicates. **e)** Absolute quantitation of the endogenous T94-NME1 peptide (VMLGETNPADSKPGTIR) in different phosphorylation states using corresponding SCAR standard peptides (VMLGETNPADSKPATIR) shows a notable amount of endogenous pyro- and triphosphorylation. The sample preparation was dependent on the phospho-species: Briefly, the respective SCAR peptide was spiked into HEK293T lysate before digestion. For the wild-type species (wt), before LC–MS/MS, an additional offline fraction was performed. Phosphorylated peptides were enriched *via* SIMAC, and the samples were fractionated before LC–MS/MS. The samples for pyro- and triphosphorylated T94-NME1 were prepared equally. The samples were treated with λ-phosphatase. Subsequently, pyro- and oligophosphorylated peptides were enriched *via* SIMAC, followed by an offline-fractionation analogue to the aforementioned samples. All samples were measured with stepped-HCD on an Orbitrap Lumos instrument using PRM, targeting all possible peptide species, including iron/aluminium adducts with and without M-oxidation. All fractions containing the peptides of interest were combined and measured with the same method. All species were identified exclusively in the oxidized state after sample preparation. For data analysis, the peak area ratio (endogenous *vs* SCAR) of the respective species (wt: V[oxM]LGETNPADSKPGTIR; p: V[oxM]LGE[ppT]NPADSKPGTIR; pp: V[oxM]LGE[ppT]NPADSKPGTIR; ppp: V[oxM]LGE[pppT+Al^3+^]NPADSKPGTIR) was used to calculate the endogenous peptide concentration normalized to the protein amount [µg] of HEK293T lysate. Validation experiments showed equal fragmentation behaviour for SCAR and endogenous standard peptides (Supplementary Fig. 17-19). Dilution series showed comparable peak areas except for the triphosphorylated species (Supplementary Fig. 20). Data are presented as mean ± SD of biological triplicates. Source data

Comparison of the different phosphorylation states by LFQ analysis indicated similar abundances of pyro-, tri- and tetraphosphorylation (Supplementary Fig. 16). To obtain absolute quantification data, wild-type and (oligo)phosphorylated SCAR (single conservative amino acid replacements) standard peptides were synthesized^47,48^. The standards were spiked into HEK293T cell lysates, which were specifically enriched for the different phospho-species before targeted parallel reaction monitoring (PRM). The measurements confirmed a notable amount of pyro- and triphosphorylated NME1—namely, 8–15%, relative to the monophosphorylated species—present in cells (Extended Data Fig. 4 and Supplementary Figs. 17–20).

### Phosphorylation by CDK1 reduces NDP kinase activity

**Fig. 4:**
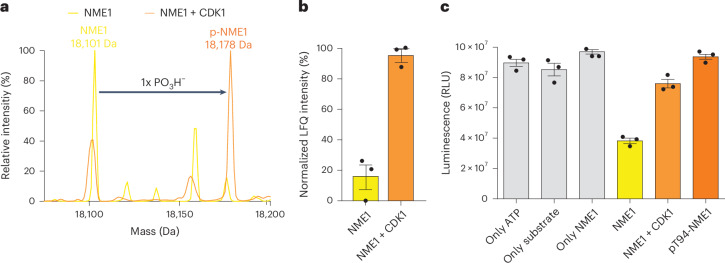
CDK1 catalyses phosphorylation of NME1 on Thr94. **a**, Deconvoluted Q-TOF-MS spectra of wild-type NME1 before (yellow) and after (orange) incubation with wild-type NME1 showed the successful phosphorylation of wild-type NME1 by CDK1. **b**, LFQ of pT94 before and after CDK1 treatment showing the relative intensity of pT94. **c**, NDP kinase activity of wild-type NME1, before and after treatment with CDK1. The activity of pT94-NME1 is shown for comparison. Assays were performed by incubating 1 μM wild-type NME1 and 50 nM CDK1 in 25 mM MOPS (pH 7.2), 10 mM MgCl_2_, 1 mM ATP and 1 mM DTT at 37 °C overnight. Data are presented as mean ± s.e. of three technical replicates. Source data

Considering the pronounced effect of a single phosphorylation event on the NDP kinase activity, and the fact that phosphorylation of Thr94 is a prerequisite for pHis118-mediated oligophosphorylation, the question arose how this phosphorylation is installed. To date, no biochemical data on protein kinases targeting Thr94 on NME1 have been reported. However, a previous high-throughput proteomic investigation identified this residue as a potential site for phosphorylation by cyclin-dependent kinase 1 (CDK1)^49^. To investigate this connection, CDK1 was incubated with wild-type NME1 in the presence of ATP and MgCl_2_. The addition of the phosphoryl group to NME1 was confirmed by Q-TOF-MS, and a substantial increase of phosphorylation on Thr94 was measured by LFQ^41^ (Fig. 4a,b; localization shown in Supplementary Fig. 21). At the biochemical level, the treatment of NME1 with CDK1 reduced NDP kinase activity, consistent with our previous observation using recombinantly expressed pS94-NME1 and pT94-NME1 (Fig. 4c).

Considering that the phosphorylation of NME1 by CDK1 can potentially instigate a whole sequence of phosphorylation events and that the different phosphorylation modes regulate NDP kinase activity, we next evaluated the structural features of NME1 in different phosphorylated states.

### ppS94-NME1 structure suggests intramolecular phosphoryl transfer

**Fig. 5:**
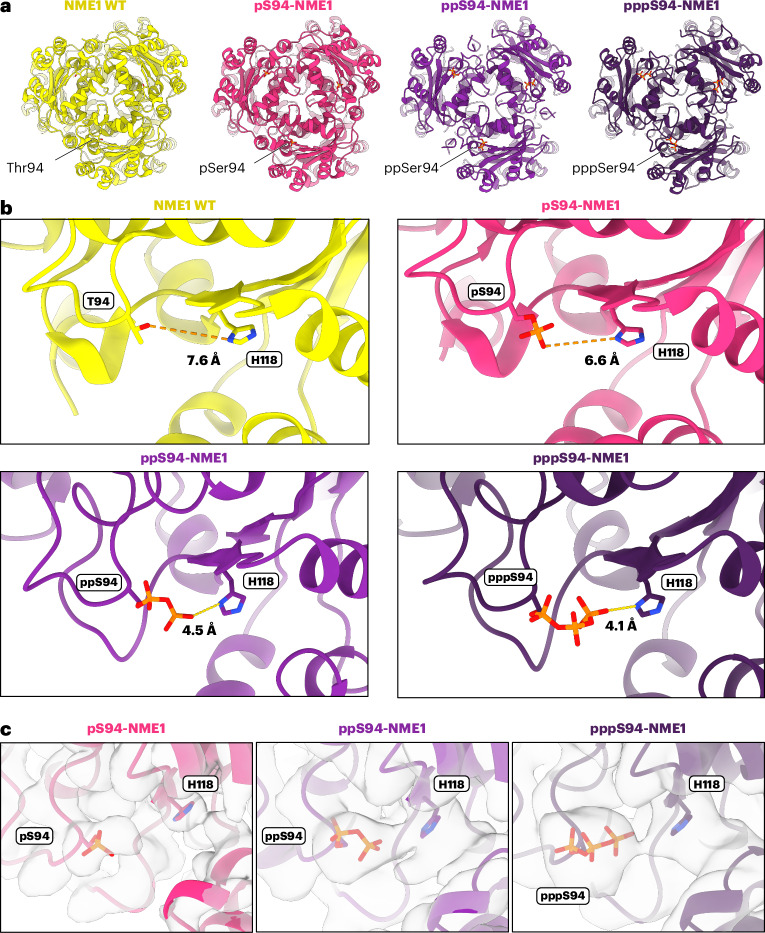
Cryo-EM structures of NME1 in different phosphorylation modes, as obtained at a resolution of 2.8 Å for pS94-NME1, 3.3 Å for ppS94-NME1 and 3.8 Å for oligo-pS94-NME1. **a**, Overview of the hexameric states of NME1 (wild-type (PDB: 7ZLW), pS94, ppS94, pppS94). Phosphoryl groups are highlighted. Note that the Ser94 residues and the attached modifications face outwards and away from the other subunits in the hexamer, making inter-subunit oligophosphorylation unlikely. **b**, Distances between the terminal atoms of side chains of residue 94 (wild-type Thr94, and phosphorylation modes on Ser94) and His118. **c**, Models of pS94, ppS94 and pppS94 in the corresponding cryo-EM maps plotted at similar thresholds, demonstrating the connecting density between ppS94 and His118 and between pppS94 and His118.

**Extended Data Fig. 5:**
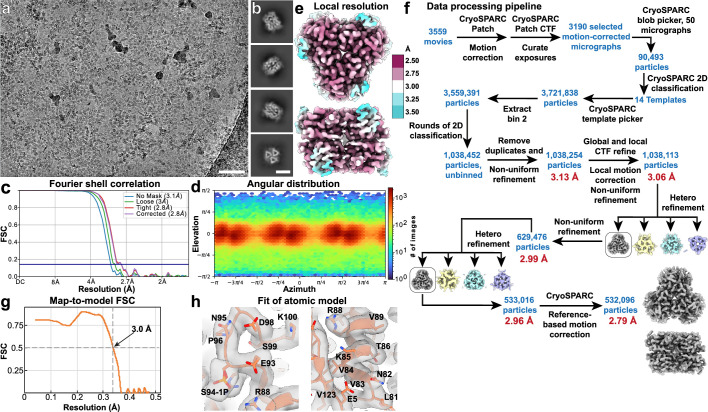
Cryo-EM and SPA of pS94-NME1. **a)** Representative cryo-EM micrograph recorded at 300 kV and -1.7 μm defocus. Scale bar: 50 nm. **b)** Representative 2D class averages, showing hexameric pS94-NME1 in side views and top view. Scale bar: 5 nm. **c,d,e)** Fourier shell correlation (**c**), angular distribution (**d**), and density map coloured by local resolution (**e**) of the final cryo-EM density map of pS94-NME1 from 532,096 particles with D3 symmetry applied. **f)** SPA data processing scheme. All steps were carried out in cryoSPARC, and all refinements were carried out with D3 symmetry. **g)** Map-to-model correlation of the pS94-NME1 model refined against the final non-sharpened density map. **h)** Fit of the atomic model in selected parts of the density map. The density maps in **e**, **f**, and **h** have been sharpened with DeepEMhancer. Source data

**Extended Data Fig. 6:**
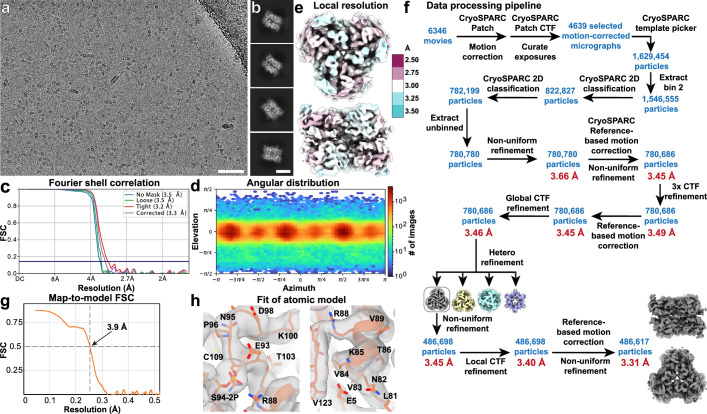
Cryo-EM and SPA of ppS94-NME1. **a)** Representative cryo-EM micrograph recorded at 300 kV and -1.9 μm defocus. Scale bar: 50 nm. **b)** Representative 2D class averages, showing hexameric ppS94-NME1 in side views and tilted views. Scale bar: 5 nm. **c,d,e)** Fourier shell correlation (**c**), angular distribution (**d**), and density map coloured by local resolution (**e**) of the final cryo-EM density map of ppS94-NME1 from 486,698 particles with D3 symmetry applied. **f)** SPA data processing scheme. All steps were carried out in cryoSPARC, and all refinements were carried out with D3 symmetry. **g)** Map-to-model correlation of the ppS94-NME1 model refined against the final non-sharpened density map. **h)** Fit of the atomic model in selected parts of the density map. The density maps in **e**, **f**, and **h** have been sharpened with DeepEMhancer. Source data

We used cryo-EM and single-particle analysis (SPA) to visualize potential structural alterations of NME1 following phosphorylation and pyrophosphorylation. For this, purified pS94-NME1 and ppS94-NME1 were vitrified, and cryo-EM datasets were recorded and refined to final resolutions of 2.8 Å for pS94-NME1 and 3.3 Å for ppS94-NME1 (Extended Data Figs. 5 and 6). Both cryo-EM maps showed that the overall hexameric structure remained unaffected by the (pyro)phosphorylation when compared to the wild-type NME1 model (PDB: 7ZLW, Fig. 5a). Notably, additional cryo-EM density at Ser94 was observed in both cases, which corresponds to the monophosphate or pyrophosphate group. The resulting model for pS94-NME1 revealed that the phosphoryl group is directed towards the His118 side chain, with a distance of 6.6 Å between the monophosphate group and the N1 of the His118 side chain (compared with 7.6 Å in wild-type NME1). The model for ppS94-NME1 indicates a more pronounced effect, and the distance between the β-phosphoryl group and the N1 of His118 amounts to only 4.5 Å (Fig. 5b). Further efforts to visualize the link between both side chains by comparing the maps at the same threshold showed that the densities for the His118 side chain and the pyrophosphate group are forming a bridge in the ppS94-NME1 map (Fig. 5c). The short distance between His118 and ppS94 and the density bridge may suggest an hydrogen bond between the N1 of His118 and the terminal phosphate group of ppS94.

**Extended Data Fig. 7:**
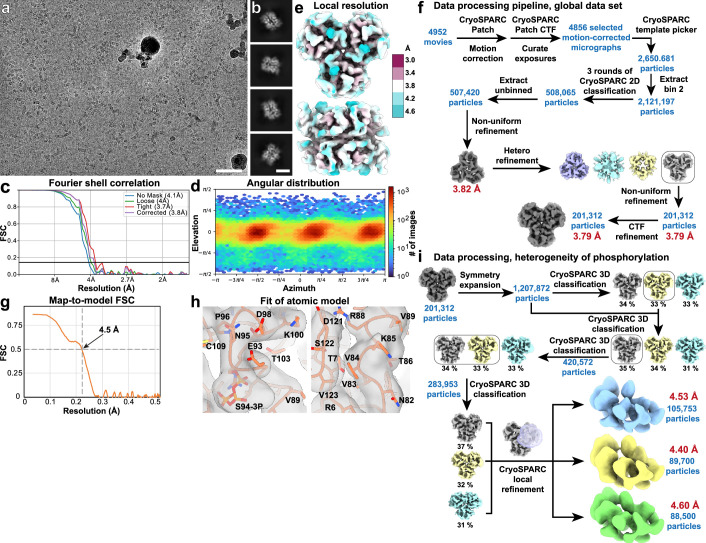
Cryo-EM and SPA of oligo-pS94-NME1. **a)** Representative cryo-EM micrograph of oligo-pS94-NME1 (that is, ppS94-NME1 with 1 mM ATP) recorded at 300 kV and -1.9 μm defocus. Scale bar: 50 nm. **b)** Representative 2D class averages, showing hexameric oligo-pS94-NME1 in side views and tilted views. Scale bar: 5 nm. **c,d,e)** Fourier shell correlation (**c**), angular distribution (**d**), and density map coloured by local resolution (**e**) of the final cryo-EM density map of oligo-pS94-NME1 from 201,312 particles with D3 symmetry applied. **f)** SPA data processing scheme applied for the global map with D3 symmetry. All steps were carried out in cryoSPARC, and all refinements were carried out with D3 symmetry. **g)** Map-to-model correlation of the pppS94-NME1 model refined against the final non-sharpened density map. **h)** Fit of the atomic model in selected parts of the density map. **i)** Data processing scheme after symmetry expansion to identify heterogeneity in phosphorylation. The density maps in **e**, **f**, and **h** have been sharpened with DeepEMhancer. Source data

Next, we investigated the structural aspects of NME1 in oligophosphorylated states and treated purified ppS94-NME1 with ATP to generate a heterogeneous mixture of oligophosphorylated NME1 for subsequent vitrification and cryo-EM data collection. After refining the data, a cryo-EM density map with an overall resolution of 3.8 Å was obtained^50^ (Extended Data Fig. 7 and Supplementary Table 1). The hexameric structure remained unaltered, even in these putatively oligophosphorylated states (Fig. 5a). Although heterogeneity posed a challenge in visualizing individual oligophosphorylation states, a larger density around ppS94 was identified. We were able to verify that this density corresponds to the pppS94 species localized at Ser94, with a likely further decrease in distance between His118 and the terminal phosphate (Fig. 5b).

**Extended Data Fig. 8:**
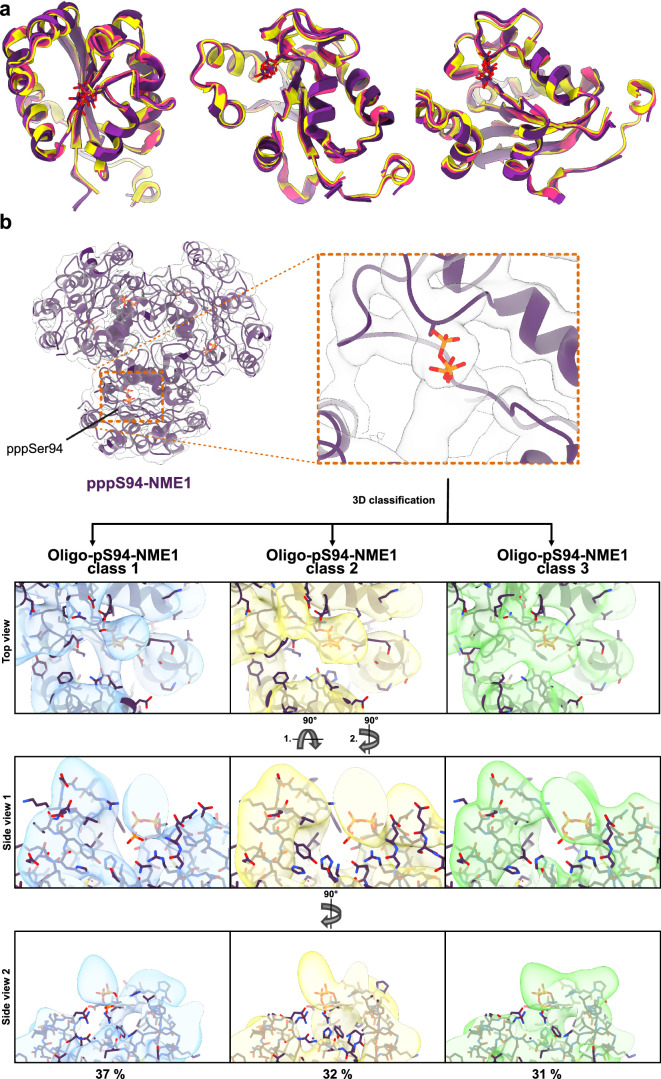
Symmetry expansion and focused 3D classification of one subunit. **a)** Overlay of one subunit of wt-NME1, pS94-NME1, ppS94-NME1, and oligo-pS94-NME1. **b)** Identification of three subsets with different extents of additional cryo-EM density appearing at the protein’s surface, shown by a top view and side view.

To visualize individual higher oligophosphorylation states in the heterogeneous dataset, symmetry expansion and focused three-dimensional (3D) classification of the phosphorylation site of one subunit were applied^51^. This analysis revealed three subsets with different extents of cryo-EM density appearing around ppSer94, which is indicative of the presence of different chain-length oligophosphates (Extended Data Fig. 8). Class 1 contained 37% of sorted particles and exhibited only little additional density, which probably corresponds to pppSer94 in a different orientation, whereas class 2 (32% of particles) and class 3 (31% of particles) had larger densities, possibly indicative of higher phosphorylation states. Furthermore, class 3 showed a connection between the additional density found for the oligophosphate chain and the density for the loop consisting of residues Lys65–Phe61, which includes the positively charged Arg58 that could interact with the negatively charged oligophosphates.

Overall, these data present high-resolution structural analyses of NME1 across higher phosphorylation states, demonstrating the reduced distance between pyrophosphorylated and phosphorylated Ser94 and the catalytic His118 side chain. The identification of additional cryo-EM density on the surface of the oligophosphorylated form by the subset classes 2 and 3 may suggest a potential role in mediating protein–protein interactions, and motivated us to identify such interactors.

### Many proteins preferentially interact with oligo-pS94-NME1

Given the known scaffolding role of inorganic polyP, and the observation that protein polyphosphorylation of yeast nuclear localization sequence-binding protein (NSR1) negatively regulated its interaction with DNA topoisomerase 1 (TOP1), we next investigated whether oligophosphorylation of NME1 might alter its interactions with protein binding partners^19,52,53^.

For an initial reference dataset, HEK293T cell lysates were prepared and subsequently incubated with immobilized wild-type NME1 or pS94-NME1 (in a buffer containing 150 mM NaCl to reduce non-specific ionic interactions). Following washing and elution, the proteins were analysed by bottom-up proteomics. We identified around 70 out of approximately 200 reported interactors of NME1^54^; however, a comparison of the LFQ values for pS94-NME1 versus wild-type NME1 suggested that hardly any of these protein interactions were affected by phosphorylation (Supplementary Fig. 22 and Supplementary Table 2).

**Fig. 6:**
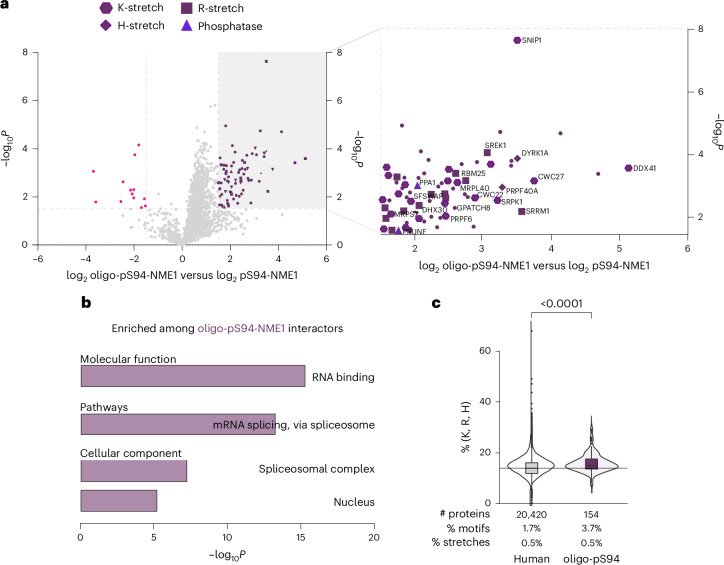
Interactome analysis of oligo-pS94-NME1 versus pS94-NME1. **a**, Volcano plot depicting LFQ values of oligo-pS94-NME1 versus pS94-NME1. The *x* axis displays the difference of LFQ values on a log_2_ scale, and the *y* axis shows the −log_10_*P* value. The left side (pink) of the volcano plot illustrates the preferential enrichment with pS94-NME1; the right side (dark purple) illustrates preferential enrichment with oligo-pS94-NME1. Proteins with a log_2_-transformed fold change >1.5 and a −log_10_*P* > 1.5 were considered to be significantly enriched. Named interactors have amino acid stretches of ≥4 H/K/R. *P* values were obtained using a two-sample *t*-test without adjustment from three biological replicates for each condition. **b**, Gene ontology analysis of proteins that preferentially interacted with oligo-pS94-NME1. *P* values were obtained using Fisher’s exact test. **c**, Percentage of positively charged amino acids over total protein length for human proteome and oligo-pS94-NME1 interactors above the significance threshold (log_2_-transformed fold change >1.5 and −log_10_*P* > 1.5) and a horizontal line highlighting the median percentage from the human proteome background. Below is the sum of matched stretches or motifs divided by all proteins for each condition. Stretch: 3–7 repeats of either K, R or H. Motif: triplet of either K, R or H allowing up to two interruptions. *P*-value was obtained using a two-sample *t*-test without adjustment. *P* = 4.3 × 10^−8^ between human proteome (*n* = 20,420) and extracted oligo-pS94 interactors (*n* = 154). Box plots were constructed as follows: the centre line indicates the median, the bounds of the box represent the interquartile range (IQR), whiskers depict values within 1.5× of the IQR, and points beyond the whiskers are plotted as individual outliers.

Next, the impact of oligophosphorylation on protein–protein interactions was explored using oligo-pS94-NME1, which was generated by treating ppS94-NME1 with ATP. Many known interactors were bound to both pS94-NME1 and oligo-pS94-NME1, exhibiting no or only low preference for either phosphorylation state (Supplementary Fig. 23 and Supplementary Table 3). However, a unique set of around 80 proteins exhibited a preferential association with oligo-pS94-NME1, based on the threshold set to log_2_ > 1.5 and −log_10_*P* > 1.5 (Fig. 6a).

**Extended Data Fig. 9:**
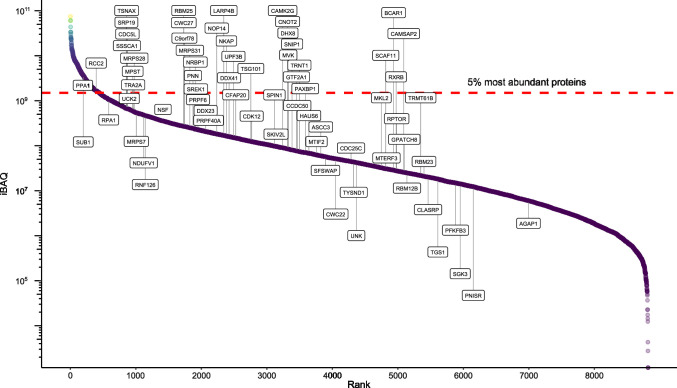
iBAQ abundance scores of protein interactors from HEK293T whole-proteome. iBAQ values were plotted against their rank in the list of iBAQs. Protein interactors of oligo-pS94-NME1 (vs. wt-NME1 and pS94-NME1) are highlighted.

To eliminate the possibility that the affinity enrichment strategy was strongly biased towards highly abundant proteins, the data were examined in relation to total protein abundance. Ranked proteins from a HEK293T whole-proteome study were plotted against their corresponding log_10_ iBAQ (intensity-based absolute quantification) values^55^. The enriched proteins spanned an abundance range over seven orders of magnitude, including several proteins of low abundance, such as CWC22 (pre-mRNA-splicing factor CWC22 homologue) and SNIP1 (smad nuclear-interacting protein 1), but high-abundance proteins were also present (Extended Data Fig. 9).

To associate the proteins significantly enriched by oligo-pS94-NME1 with pathways or functions, we used Enrichr for gene ontology analysis^56^^,^^57^ (Fig. 6b). This analysis suggests a connection of oligo-pS94-NME1 to mRNA splicing via the spliceosome because proteins from this pathway are overrepresented among the interactors (CDC5L, PRPF40A, DDX41, CWC27, CWC22, SRRM1, SRPK1, SART1, PNN, PRPF6, TRA2B, SNIP1, RNPS1, PPIL3). The overrepresentation of proteins involved in mRNA splicing is consistent with the association with the spliceosomal complex (cellular component) and RNA binding (molecular function) (Supplementary Table 4).

Additionally, we observed an interaction between tyrosine phosphorylation-regulated kinase 1A (DYRK1A) and oligo-pS94-NME1. DYRK1A was recently shown to non-covalently bind to inorganic polyP via strong ionic interactions with polyhistidine stretches, which in turn negatively regulated the in vitro activity^20^. Because previous reports have showcased the ionic interactions with polyhistidine/polylysine stretches^22^, we consequently screened the list of oligo-pS94-NME1 interactors for stretches containing consecutive H, K or R residues and shorter interrupted H/K/R rich motifs, such as HxHH and HxxHH. We found that the majority of the interactors indeed harboured such positively charged motifs (Fig. 6c and Supplementary Tables 5 and 6). Furthermore, we determined the percentage composition of stretches and shorter interrupted motifs of all proteins preferentially enriched in the oligo-pS94-NME1 dataset and compared it to the HEK293T proteome. Interestingly, a significant enrichment of these motifs was present among the oligo-pS94-NME1-interacting proteins (Fig. 6c).

Finally, two phosphatases, exopolyphosphatase PRUNE1 and inorganic pyrophosphatase 1 (PPA1), that exhibited a preferential interaction with oligo-pS94-NME1 were identified. However, in biochemical assays, these phosphatases did not show any hydrolytic activity towards the corresponding (oligo)phosphorylated NME1 peptides (Supplementary Figs. 15 and 24–27), illustrating the need to further investigate the metabolism of oligophosphorylated species in the future.

## Discussion

NME1 continues to be a multifaceted enzyme with a diverse range of functions. We initially set out to investigate the influence of phosphorylation and pyrophosphorylation near the substrate-binding site on the structure and function of NME1. To do so, genetic code expansion to produce stoichiometrically phosphorylated NME1 was combined with a chemical strategy to convert the phosphoprotein to the corresponding pyrophosphoprotein. When the NDP kinase activity of pS/pT94-NME1 and ppS94-NME1 was assessed, a strong, progressive reduction in activity was observed, compared with wild-type NME1. It seems probable that the presence of the negatively charged (pyro)phosphoryl group leads to strong electrostatic repulsion between the modification and the triphosphate moiety of ATP, slowing the rates of formation of the pHis intermediate. Similarly, subsequent binding of the nucleoside diphosphate will be slowed down, resulting in an overall decrease in catalytic activity. This observation is consistent with our previous findings where (pyro)phosphorylation close to the substrate-binding site of NAGK resulted in a strong reduction of enzymatic activity^36^.

As in the case of pyrophospho-NAGK, the formation of ppS94-NME1 was autocatalytic and ATP dependent. Unexpectedly, ppS94-NME1 proved competent to undergo subsequent ATP-dependent ‘hyperphosphorylation’, which turned out to be an auto-oligophosphorylation event. Using MS/MS analysis with stepped-HCD, covalently attached oligophosphate—up to a hexaphosphate chain—could be detected and localized to Ser94. Formation of the pyro- and oligophosphate chains was found to be dependent on the catalytic histidine residue His118 because the kinase-inactive p/ppS94-NME1(H118F) mutants lacked any signs of auto-oligophosphorylation.

Importantly, the stepped-HCD MS/MS analysis also uncovered the presence of oligophosphorylated NME1 in lysates from HEK293T cells, thus adding oligophosphorylation as a potential new mode of endogenous phosphoregulation. This observation then raises the question of whether oligophosphorylation is restricted to NME1 or if this modification occurs more broadly in eukaryotic cells. The initial MS/MS analysis already highlighted the complexities of detecting oligophosphorylated peptides—such as metal ion adduct formation—and warrants the development of a dedicated oligophospho-proteomics workflow. Such a development will greatly benefit from the availability of oligophosphorylated peptide standards so that conditions for lysate preparation, peptide enrichment and MS fragmentation methods can be systematically explored and optimized^47^.

Our hypothesis that NME1 oligophosphorylation proceeds via an intramolecular mechanism is supported by the cryo-EM data for phosphorylated, pyrophosphorylated and oligophosphorylated NME1. Both the phosphorylated and pyrophosphorylated side chains are oriented towards His118, with a distance that would be compatible with an intramolecular transfer. As such, oligophosphorylation appears to result from a nucleophilic attack of the p/ppS94 residue on a 1-pHis118 intermediate. The structural data suggest that the β-phosphoryl group of ppS94 is optimally placed (within 4.5 Å from His118) for a nucleophilic attack, and in closer proximity to the 1-pHis118 intermediate than the pS94 residue. This observation is corroborated by the biochemical data, which showed that ppS94-NME1 was much more competent to undergo oligophosphorylation, compared with pS94-NME1. In fact, when pS94-NME1 was treated with ATP, detection of the pyrophosphorylated intermediate was difficult because it appeared to subsequently convert to the tri- and tetraphosphorylated species at a much higher rate. In addition, it is clear from our structural data that the tri- and tetraphosphorylated species are formed, but with a heterogeneous orientation of the oligophosphate chains (Extended Data Fig. 8). It will be interesting to investigate in the future if certain stimuli, or added factors, can promote the ‘rate-limiting’ addition of the β-phosphoryl group—thereby facilitating auto-oligophosphorylation.

Oligophosphorylated NME1 displayed a notable amount of cryo-EM density at the surface of the NME1 hexamer, and it seems plausible that oligophosphorylation could act as a recruiting element to engage a different set of NME1 interaction partners. Indeed, many new interactors could be identified upon oligophosphorylation. The majority of these proteins harboured stretches of polyhistidine, polylysine or polyarginine and are predominantly associated with RNA splicing via the spliceosome. Although the role of oligophosphorylation in these pathways remains to be determined, a simple explanation could be that the high negative charge of the oligophosphate modification resembles the negatively charged RNA backbone, thereby targeting a similar set of proteins.

The ability to catalyse the formation of oligophosphate chains is not an exclusive feature of NDP kinases. Many other enzymes across species are competent to catalyse phosphoanhydride bond formation^58^. One set of enzymes encompasses the bacterial polyphosphate kinases PPK1 and PPK2, which are involved in the generation of long chains of inorganic polyphosphate^52^. PPK2 is also known to have NDP kinase activity by catalysing the transfer of phosphoryl groups from inorganic polyP to an NDP, while PPK1 catalyses the synthesis of inorganic polyP from ATP using a pHis intermediate^59–61^. Interestingly, these two functionalities seem to be united in NME1.

Given the remarkable reactivity of the pHis118-NME1 intermediate, the ability to generate oligophosphate chains may also be present in other human enzymes that harness a pHis intermediate. Interesting candidates include other proteins from the NME family (NME2–9), serine/threonine-protein phosphatase 5 (PGAM5), phosphoglycerate mutase 1 (PGAM1), 6-phosphofructo-2-kinase bisphosphatase (PFKFB3), and the kinase substrates of NME1, including succinyl CoA synthetase (SUCLG1) and ATP-citrate lyase (ACLY)^62–64^. For example, the crystal structure of PGAM5 illustrates the close proximity between the catalytic H105 and Y108 (5.6 Å)^62^. As Y108 is a known phosphorylation site, one could speculate that subsequent oligophosphorylation may take place^65^ (Supplementary Fig. 28).

In principle, oligophosphorylation does not have to be an intramolecular process, but could also occur intermolecularly. This may also be the case for NME1, which could—assuming it is provided with the appropriate phosphoprotein substrate— act as an oligophosphate kinase. Therefore, much remains to be explored regarding the extent, the functional relevance and the regulation of protein oligophosphorylation. All of the hypotheses and questions raised above will require the development of new analytical tools that can decipher, distinguish and quantify all the different phosphorylation states of a given peptide or protein. The structural and MS methods reported here provide the necessary starting points and should be built upon in the future.

## Methods

### General information

All solvents and reagents were purchased from commercial sources and used without further purification. All aqueous solutions were prepared using ultrapure laboratory-grade water (deionized and filtered). Site-directed mutagenesis was performed on a Bio-Rad C1000 Touch Thermal Cycler. All bacterial growth media and cultures were handled using sterile conditions. Antibiotics were prepared as stock solutions at a concentration of 1,000× and stored at −20 °C. Protein concentrations were determined with the Pierce Coomassie (Bradford) Protein-Assay-Kit (Thermo Fisher Scientific). Mass spectra were acquired on an Agilent 6220 TOF Accurate Mass coupled to an Agilent 1200 LC (Agilent Technologies). Intact proteins were analysed using a Waters H-class instrument equipped with a quaternary solvent manager, a Waters Sample Manager-FTN, a Waters PDA detector and a Waters Column Manager with an Acquity UPLC protein BEH C4 column (300 Å, 1.7 μm, 2.1 mm × 50 mm). Liquid chromatography (LC)–MS/MS analysis was performed using an UltiMate 3000 RSLC nano-LC system coupled online to an Orbitrap Fusion operated using Xcalibur software v.3.4 or an Orbitrap Fusion Lumos mass spectrometer operated using Xcalibur software v.4.1 (Thermo Fisher Scientific). HEK293T cells (ATCC, CRL-3216) were grown in 15-cm dishes to 70–80% confluency in Dulbecco’s Modified Eagle’s Medium (DMEM), supplemented with 10% FBS (Gibco), 1× penicillin–streptomycin (Gibco), and glutamine (Gibco) (2 mM) in a 5% humidified CO_2_ incubator at 37 °C. Cell counting was performed with a Bio-Rad TC-20 Cell counter. Antibodies used in this study for western blot analysis were: anti-Nl-Phosphohistidine (1-pHis) antibody, clone SC50-3 (Sigma-Aldrich, MABS1341, dilution 1:1,000); anti-NMEl antibody (Cell Signaling Technology, 3345, dilution 1:1,000); and anti-rabbit IgG, HRP-linked antibody (Cell Signaling Technology, 7074, dilution 1:1,000). The following phosphatases were used: alkaline phosphatase (FastAP, Thermo Fisher Scientific), inorganic pyrophosphatase (rhPPA1, Bio-Techne), Lambda Protein Phosphatase (New England Biolabs) and hPRUNE1 (expressed and purified according to previously published protocols)^66^.

### Generation of expression plasmids

The NME1 plasmid was obtained from GeneArt Gene Synthesis (Thermo Fisher Scientific) (pCDF-NME1 T94 amber codon full length); the NME1 plasmid (pNIC28-Bsa-NME1) was a gift from the Kelly laboratory (The Scripps Research Institute).

### Site-directed mutagenesis

Plasmid DNA harbouring the corresponding NME1 ORF was extracted from an overnight culture of the pET21-NAGK vector in TB-Kan using a QIAGEN Miniprep kit. PCR reactions (50 µl) were set up following the NEB Phusion High-Fidelity DNA polymerase protocol using the following temperature programme: 5 min 98 °C → 30 s 98 °C → 3 min 62 °C → cycle to step 2 30× → 5 min 72 °C. The primers 5′-GCTGGGTGAGTAGAATCCTGCTG-3′ and 3′-ATCACGCGACCTGTCTTT-5′ were used to incorporate the amber codon at position 94.

The primers 5′-CAACATCATTTTTGGGTCGGACAG-3′ and 3′-CGGCCTACCTGAATACAG-5′ were used to replace His118 with H118F.

### Expression and purification of His_6_-tagged wt-NME1

Chemically competent *E. coli* was transformed by heat-shock with plasmids containing wt-NME1 (pNIC28-Bsa-NME1). The cells were cultured in the presence of kanamycin (50 μg ml^−1^) and incubated overnight at 37 °C. The overnight culture was diluted to an OD_600_ of 0.05 in 1 litre of LB medium supplemented with kanamycin (50 μg ml^−1^) and cultured at 37 °C, 150 r.p.m. until an OD_600_ of 0.6–0.8 was reached. Isopropylthiogalactoside (IPTG) was added to a final concentration of 1 mM, protein expression was induced for 18 h at 18 °C, and Co-NTA affinity purification was performed using a fast protein liquid chromatography (FPLC) system. After purification, the fractions containing the protein were collected and concentrated, and the buffer was exchanged (50 mM Tris–HCl (pH 7.8), 150 mM NaCl, 1 mM dithiothreitol (DTT) and 10% glycerol) using Amicon centrifugal filter units with a 10-kDa molecular weight cut-off (MWCO; Millipore).

### Expression and purification of His_6_-tagged pT94-NME1

Chemically competent *E. coli* BL21 (DE3) ΔserCΔycdX was co-transformed by heat-shock with plasmids containing both pUC plasmid and pT94-NME1 (pET151a). The cells were cultured in the presence of kanamycin (50 μg ml^−1^) and ampicillin (50 μg ml^−1^) and incubated overnight at 37 °C. The overnight culture was diluted to an OD_600_ of 0.05 in 4 litres of TB medium supplemented with kanamycin (50 μg ml^−1^) and ampicillin (50 μg ml^−1^) and cultured at 37 °C, 150 r.p.m. until an OD_600_ of 0.6–0.8 was reached. IPTG was added to a final concentration of 1 mM, protein expression was induced for 18 h at 18 °C, and Co-NTA affinity purification was performed using an FPLC system. After purification, the fractions containing the protein were collected and concentrated, and the buffer was exchanged (50 mM Tris–HCl (pH 7.8), 150 mM NaCl, 1 mM DTT and 10% glycerol) using Amicon centrifugal filter units with a 10-kDa MWCO (Millipore).

### Expression and purification of His_6_-tagged pS94-NME1 and pS94-NME1(H118F)

Electrocompetent *E. coli* BL21 (DE3) ΔserB was sequentially transformed by electroporation (voltage, 1,800 V; capacitance, 25 μF; resistance, 200 Ω; gap length, 1.0 mm) with first pKW1-Sep (Camp^R^) and then pNIC28-Bsa (Kan^R^) containing pS94-NME1 or pS94-NME1(H118F). The cells were cultured in the presence of kanamycin (50 μg ml^−1^) and chloramphenicol (25 μg ml^−1^) and incubated overnight at 37 °C. The overnight culture was diluted to an OD_600_ of 0.05 in 4 litres of TB medium, supplemented with kanamycin (50 μg ml^−1^) and chloramphenicol (25 μg ml^−1^), and cultured at 37 °C, 150 r.p.m. until an OD_600_ of 0.6–0.8 was reached. IPTG was added to a final concentration of 1 mM, and additionally, (l)-*O*-phosphoserine (pH 7.0) was added to a final concentration of 2 mM. Protein expression was induced for 18 h at 18 °C, and Co-NTA affinity purification was performed using an FPLC system. After purification, the fractions containing the protein were collected and concentrated, and the buffer was exchanged (50 mM Tris–HCl (pH 7.8), 150 mM NaCl, 1 mM DTT and 10% glycerol) using Amicon centrifugal filter units with a 10-kDa MWCO (Millipore).

### Nucleoside diphosphate kinase activity assay

Purified NME1 (1 nM, 10 nM, 100 nM or 1,000 nM) was added to a reaction mixture (50 µl) containing 50 mM Tris–HCl (pH 8.0), 150 mM NaCl, 10 mM MgCl_2_, 900 μM GDP or TDP, and 100 μM ATP. Additionally, 100 μM Fe_2_(SO_4_)_3_ or Al_2_(SO_4_)_3_ were added if indicated. After 1 h at 37 °C, Promega Kinase-Glo Plus reagent was added in a white 384-well plate (1:1 with reaction mixture, 10 µl + 10 µl) and after 10 min of equilibration at room temperature, the luminescence signals were read out with a Tecan Infinite M Plex plate reader using an exposure time of 100 ms. Data were plotted and analysed using GraphPad Prism v.5.

### NME1 in vitro autophosphorylation

In vitro autophosphorylation of purified NME1 (50 ng μl^−1^) was performed by incubating with 1 mM ATP in TMD buffer (20 mM Tris–HCl (pH 8.0), 5 mM MgCl_2_ and 1 mM DTT) at 37 °C for 1 h. Reactions were stopped by the addition of 5× sample buffer (5× = 10% SDS, 250 mM Tris–HCl (pH 8.8), 0.02% bromophenol blue, 50% glycerol, 50 mM EDTA, 500 mM DTT; pH 8.8) and analysed by SDS-PAGE and Q-TOF-MS.

### NME1 in vitro phosphorylation by CDK1

A mixture of 1 µM wt-NME1 and 50 nM CDK1 was incubated in 25 mM MOPS (pH 7.2), 10 mM MgCl_2_, 1 mM ATP and 1 mM DTT at 37 °C overnight, and subsequently analysed by Q-TOF-MS, MS/MS and NDP kinase activity assay.

### Generation of pyrophosphorylated NME1

For the chemical pyrophosphorylation of pS94-NME1, the reported protocol^36^ was used with an alternative refolding buffer (50 mM Tris–HCl (pH 8.5), 35 mM KCl, 0.3 mM GSSG, 3 mM GSH, 10 mM EDTA, 0.2% CHAPS).

### Q-TOF-MS

High-resolution electrospray ionization MS spectra were recorded on two different instruments: (1) Agilent 6220 TOF Accurate Mass coupled to an Agilent 1200 LC (Agilent Technologies), measured at 35 °C between 100 and 2,000 *m*/*z*. Column, Accucore RP-MS (30 × 2.1 mm; 2.6-μm particle size); flow rate, 0.8 ml min^−1^; solvents, A = H_2_O + 0.1% TFA, B = MeCN + 0.1% TFA. Gradient, 5% B for 0–0.2 min, 5–99% B for 0.2–1.1 min, 99% B for 1.1–2.5 min. (2) Agilent Technologies 6230 Accurate Mass TOF LC/MS linked to Agilent Technologies HPLC 1260 Series. Column, Thermo Accucore RP-MS; particle size, 2.6 µM; dimensions, 30 × 2.1 mm; flow rate, 0.8 ml min^−1^; Solvents, A = H_2_O + 0.1% FA, B = MeCN + 0.1% FA; gradient, 5% B for 0.0–0.2 min, 5–99% B for 0.2–1.1 min, 99% B for 1.1–3.6 min, 5% B for 3.6–4.9 min; UV detection, 220 nm, 254 nm, 300 nm.

### Optimizing the fragmentation of oligophosphorylated peptides

Based on the obtained modification masses, an inclusion list was generated, and samples were reanalysed using a targeted method. LC–MS/MS analysis was performed using an UltiMate 3000 RSLC nano-LC system coupled online to an Orbitrap Fusion mass spectrometer (Thermo Fisher Scientific). For sample loading, a PepMap C18 trap column (Thermo Fisher Scientific; 0.075 mm internal diameter × 50 mm length; particle size, 3 μm; pore size, 100 Å) was used. The loading mobile phase A contained 1% MeCN and 0.05% TFA in water, and mobile phase B 0.05% TFA in MeCN. Reversed-phase separation was performed using a 50-cm analytical column (in-house packed with Poroshell 120 EC-C18, 2.7 μm, Agilent Technologies) with mobile phase A containing 0.1% FA in water, and mobile phase B 0.1% FA in MeCN, using a 93 min gradient (4–5% B for 0–8 min; 5–25% B for 8–74 min; 25–28% B for 74–80 min; 28–31% B for 80-86 min; 31–36% B for 86-92 min; 36-40% B for 92–95 min; 40–50% B for 95-96 min; 50–80% B for 96–101 min; 80% B for 101–104 min; 80–4% B for 104–104.1 min). Data were acquired using survey scans in a range of 380–1,400 *m*/*z* with a resolution of 120,000 and an automatic gain control (AGC) target value of 4 × 10^5^. If a feature with *m*/*z* and *z* matching a peptide from the inclusion list was observed, 20 independent fragmentation events were triggered (Supplementary Fig. 10). Fragment spectra were recorded in an Orbitrap mass analyser using a resolution of 30,000 and a maximum injection time of 100 ms with an AGC target of 50,000. Dynamic exclusion of 30 s was enabled after two full fragmentation cycles. Raw files were analysed as described above, and the obtained PSMs were manually inspected for fragmentation efficiency and unambiguous site localization.

### In solution tryptic digestion for relative quantification experiments

Lyophilized samples were resolubilized in 100 µl buffer (50 mM TEAB, 6 M urea) and diluted threefold. Samples were subsequently reduced and alkylated with 5 mM TCEP and 20 mM iodoacetamide (IAA) at 37 °C for 1 h. Trypsin was added at an enzyme-to-protein ratio of 1:100 (w/w) to digest overnight at 37 ˚C. Trypsin was then quenched with 1% FA (final concentration) and centrifuged for 10 min at 20,000*g*. The supernatant was collected and desalted using Sep-PAK C18 cartridges and lyophilized. Peptides were quantified by bicinchoninic acid assay.

### LC–MS

LC–MS/MS analysis was performed using an UltiMate 3000 RSLC nano-LC system coupled online to an Orbitrap Fusion mass spectrometer (Thermo Fisher Scientific) with instrument control software v.3.4. For sample loading, a PepMap C-18 trap column (Thermo Fisher Scientific; 0.075 mm internal diameter × 50 mm length; particle size, 3 μm; pore size, 100 Å) was used. The loading mobile phase A contained 1% MeCN and 0.05% TFA in water, and mobile phase B 0.05% TFA in MeCN. Reversed-phase separation was performed using a 50-cm analytical column (in-house packed with Poroshell 120 EC-C18, 2.7 μm, Agilent Technologies) with mobile phase A containing 0.1% FA in water, and mobile phase B 0.1% FA in MeCN. The gradient started with 4% buffer B, reaching 40% buffer B, with a total run time of 80 min or 120 min, including column wash and equilibration. MS1 scans were performed in the Orbitrap using the following settings: resolution, 120,000; AGC target, 400,000; scan range, 375–1500 *m*/*z*; maximum injection time, 50 ms. In 2-s cycles, precursors with a charge of 2–4 were sent to MS2 and excluded from fragmentation for 20 s. Depending on the purpose of the experiment, MS2 scans were acquired in the Orbitrap with the following settings: (1) isolation window, 1.6 *m*/*z*; resolution, 15,000; HCD, 23% or 30%; scan range, automatic; maximum injection time, 22 ms; AGC target, 50,000; (2) isolation window, 1.6 *m*/*z*; resolution, 60,000, EThcD with SA energy, 30%; scan range, 200–3,000 *m*/*z*; maximum injection time, 500 ms; AGC target, 100,000; (3) isolation window, 1.4 *m*/*z*; resolution, 30,000; stepped-HCD with collision energies of 20%, 23% and 26%; scan range, automatic; maximum injection time, 100 ms; AGC target, 100,000.

For targeted measurements used for the endogenous absolute quantification and validation experiments, the described stepped-HCD method was adapted by removing the dynamic exclusion and implementing corresponding inclusion lists. All possible peptide species for the SCAR (VMLGETNPADSKPATIR) and endogenous (VMLGETNPADSKPGTIR) sequence, including all phosphorylation states up to tetraphosphorylation, were targeted. This also included all possible iron and aluminium adducts and the potential M-oxidation.

### Analytical characterization of oligophosphorylated NME1

The obtained raw data were analysed with FragPipe v.21 using the built-in open-search workflow^41^ and NME1_T94S plus contaminants as search space. The following MSFragger settings were applied: precursor mass tolerance, −50 to +600 Da; fragment mass tolerance, ±20 ppm; mass calibration and parameter optimization, enabled; isotope error, 0; enzyme, trypsin (cuts after K and R, no cut before P) with two missed cleavages; peptide length, 7–50 amino acids; peptide mass range, 500–5,000 Da; variable modifications, oxidation (M, +15.9949 Da, up to 3×), acetylation (N-terminal, +42.0106 Da); carbamidomethylation of C was set as fixed modification (+57.02146 Da). Validation was performed using PeptideProphet (using default settings) and ProteinProphet (Philosopher v.5.1.0). A protein-level false discovery rate (FDR) of 50% was applied. Additionally, PTM-Shepard^67^ was enabled (using the default settings). Identified peptides containing a modified Ser94 were filtered for at least five PSMs of the corresponding modification mass over all replicates. The best-scoring PSM of each modification was manually validated and blotted using an interactive spectrum annotator.

### Determination of the normalized extracted ion chromatogram area

The normalized extracted ion chromatogram (XIC) area (%) was calculated using FreeStyle 1.7 by integrating the precursor area of a species in the MS1 relative to the integrated area of the full chromatogram to determine the contribution of the individual species to the total MS signal. Values obtained from this calculation were normalized to the species with the highest contribution.

### Enrichment workflow for endogenous identification of oligophosphorylation

The enrichment sample preparation workflow was performed as described in Morgan et al.^23^ with minor adaptations. Experimental procedures can be found in the Supplementary Information.

### Identification of oligophosphorylated peptides from enriched HEK293T lysate

Raw data were analysed with FragPipe (v.21) using the built-in offset-search workflow^41^ and NME1_T94S plus contaminants, NME1 plus contaminants, NME2 plus contaminants, or the human proteome plus contaminants as search space. The following MSFragger settings were applied: precursor mass tolerance, ±10 ppm; fragment mass tolerance, ±20 ppm; mass calibration and parameter optimization, enabled; isotope error, 0/1; enzyme, trypsin with two missed cleavages; peptide length, 7–50 amino acids; peptide mass range, 500-5,000 Da; variable modifications, oxidation (M, +15.9949 Da, up to 3×), acetylation (N-terminal, +42.0106 Da); carbamidomethylation of C was set as fixed modification (+57.02146 Da). Additionally, the following masses were considered as offset-mass: 0.0, 101.947, 103.9256, 105.9816, 122.973, 132.8784, 136.9884, 141.923, 159.933, 173.9882, 177.9424, 181.9128, 184.0552, 197.8802, 199.8884, 201.901, 209.0186, 212.845, 216.9552, 226.8986, 228.8408, 230.8544, 263.8566, 269.8656, 292.8104, 343.823, 361.8338, 372.7767, 389.8042, 390.788, 423.7912, 452.7431, 527.7141, 585.6209, 79.9666, 93.9798, 94.0186, 95.9618, 98.0492. Validation was performed with PeptideProphet (using default settings) and ProteinProphet (Philosopher v.5.1.0). A protein-level FDR of 1% was applied. Additionally, PTM-Shepard^3^ was enabled (using the default settings).

### HEK293T cell culture and lysate processing for interactome analysis

HEK293T cells were grown in 15-cm dishes to 70–80% confluency in DMEM, complemented with 10% FBS, penicillin–streptomycin (100 U ml^−1^) and glutamine (2 mM). Cells were washed twice with ice-cold DPBS (10 ml) and lysed by sonication (IKA Labortechnik, U200S control, 0.5 cycles, 50% intensity, five rounds) in 50 mM TBS buffer supplemented with phosphatase and protease inhibitors (Roche PhosStop and cOmplete EDTA-free protease inhibitor cocktail). The cells were scraped off, transferred to protein low-binding microcentrifuge tubes and incubated on ice for 10 min. The lysate was then centrifuged at 4 °C for 10 min at 17,900*g*. The supernatants were combined, and lysate protein concentration was determined using a Pierce Coomassie (Bradford) protein assay kit.

### Affinity capture experiments for proteomic analysis

All steps were conducted at 4 °C. A suspension of nickel beads (50 µl) was washed three times with 1 ml Milli-Q H_2_O and three times with 1 ml TBS buffer (50 mM Tris–HCl (pH 7.5), 150 mM NaCl) supplemented with 2 mM MgCl_2_ and 2 mM MnCl_2_. Subsequently, 25 µg of recombinant NME1 (wild-type, T94pS, ATP-treated T94ppS) in 100 µl TBS buffer was added to the beads and incubated with constant rotation for 1 h at 4 °C. Beads were centrifuged at 2,000*g*, and the supernatant was discarded. The beads were washed three times with 1 ml TBS buffer, 1 mg of HEK293T cell lysate in TBS buffer was added to the beads, and the mixture was incubated for 3 h at 4 °C under rotation. The beads were centrifuged at 2,000*g*, the supernatant was discarded and the beads were washed three times with 1 ml TBS buffer. Finally, the beads were incubated with elution buffer (50 mM Tris–HCl (pH 7.5), 150 mM NaCl, 500 mM Imidazole) for 1 h at 4 °C under constant rotation. After centrifugation at 2,000*g*, the supernatant was collected and lyophilized.

### Identification, quantification and statistics of interactome data

Raw data were analysed and processed with MaxQuant software v.2.0.3.0. Analysis was done with standard settings. Search parameters included two missed cleavage sites, fixed cysteine carbamidomethyl modification, and variable modifications including methionine oxidation and N-terminal protein acetylation. The peptide mass tolerance was 4.5 ppm for MS scans and 20 ppm for MS/MS scans. The match between runs option was enabled. Database search was performed using Andromeda against the Human UniProt/Swiss-Prot database with common contaminants. The FDR was set to 1% at both the peptide and protein levels. Protein quantification was done based on razor and unique peptides. Label-free quantification was enabled. Bioinformatic analysis was carried out with Perseus software v.1.6.7.0. Proteins were filtered to exclude reverse database hits, potential contaminants and proteins only identified by site. Proteins were further filtered by rows, requiring a valid value for at least two proteins out of four technical replicates. Data was imputed using the Perseus default parameters: width, 0.3; downshift, 1.8. Volcano plots were generated based on the log_2_-transformed fold change and the −log_10_*P* derived from a *t*-test (number of randomizations, 250).

### Assessment of primary sequence motifs

Proteomics data analysis and visualization were conducted using R (RStudio v.2024.04.2). Proteins enriched with oligo-pS94-NME1 (log_2_ fold change >1.5) were compared to all 20,421 human protein sequences retrieved on 7 October 2024. Metrics derived from the primary amino acid sequence included the number of aromatic and charged residues, and the net charge per residue. The overall hydrophobicity of proteins was calculated based on the Kyte & Doolittle scale using the peptides R package^68^. Modified residues were retrieved from UniProt. Stretches of positively charged residues were identified as three to seven consecutive repeats of the same amino acid (Lys, His or Arg). Additionally, positively charged motifs were identified when a positively charged amino acid appeared three times, interrupted by up to two other amino acids, such as KxKK or KKxK.

### Cryo-EM sample preparation and data acquisition

For pS94-NME1 and ppS94-NME1, purified protein was vitrified on Quantifoil 1.2/1.3 Cu 300 mesh grids at 0.9 mg ml^−1^ using a Vitrobot Mark IV set to a blot force of 0 and a blotting time of 3.0 s. For oligo-pS94-NME1, ppS94-NME1 was incubated at 1 mM concentration with ATP for 3 h at 37 °C before vitrification as above. Micrographs for all three datasets were acquired using a FEI Titan Krios G3i microscope (Thermo Fisher Scientific) operated at 300 kV equipped with a Bioquantum K3 direct electron detector and energy filter (Gatan) running in CDS counting mode at a slit width of 20 eV and a nominal magnification of 105,000×, giving a calibrated physical pixel size of 0.83 Å per pixel on the specimen level. EPU 2.12 was utilized for automated data acquisition with AFIS enabled. For pS94-NME1, movies were recorded for 2.0 s, accumulating a total electron dose of 47.7 *e*^−^ Å^−^^2^ fractionated into 50 frames. Nominal defocus values were between −1.1 and −2.6 µm. For ppS94-NME1, movies were recorded for 2.0 s, resulting in a total electron dose of 44.6 *e*^−^ Å^−^^2^ fractionated into 50 frames with nominal defocus values between −1.2 and −2.4 μm. For oligo-pS94-NME1, movies were recorded for 2.0 s, accumulating a total electron dose of 44.1 *e*^−^ Å^−^^2^ distributed over 50 frames. Here, nominal defocus values were between −1.4 and −2.4 μm.

### Data processing of pS94-NME1, ppS94-NME1 and oligo-pS94-NME1

All data-processing steps were carried out using CryoSPARC and are outlined in Supplementary Figs. 12–14^69^. Obtained movies were aligned using Patch Motion correction, and contrast transfer function (CTF) was determined using patch CTF estimation. After sorting out bad images, micrographs were selected for further processing. Particles were picked using the blob picker in cryoSPARC and extracted for initial 2D classification and generation of autopicking templates. These were used in subsequent template-based autopicking, where 3,559,391, 1,546,555, and 2,121,197 particles have been identified and extracted after particle curation for pS94-NME1, ppS94-NME1 and oligo-pS94-NME1, respectively. After three rounds of 2D classification (70 online-expectation–maximization iterations), an initial 3D heterorefinement (three classes) and duplicate removal, particles were re-extracted without binning and subjected to non-uniform refinement using *D*3 symmetry and a molecular map of PDB 5UI4 (low-pass-filtered to 12 Å) as an initial model^70^. After iterative rounds of global and local CTF refinement and local motion correction, particles were sorted in two successive rounds of 3D heterorefinement (four classes each), resulting in a final class of particles^71^. These were then further processed using non-uniform refinement, followed by reference-based motion correction, yielding 532,096 (for pS94-NME1), 486,617 (for ppS94-NME1) and 201,312 (oligo-pS94-NME1) particles. A final non-uniform refinement resulted in a resolution of 2.8 Å (for pS94-NME1), 3.3 Å (for ppS94-NME1) and 3.79 Å (for oligo-pS94-NME1) according to the gold-standard Fourier Shell Correlation criterion. DeepEMhancer^72^ was applied for map sharpening. A detailed description of the data processing can be found in Extended Data Figs. 5-7.

### Atomic modelling of pS94-NME1

The atomic model of human NME1 (PDB: 5UI4) was used as a starting model, and the attached imidazole fluorosulfate group and water molecules were removed. The model was rigid-body fitted into the sharpened density map using UCSF ChimeraX^73^, manually adjusted in Coot^74^, where Thr94 was mutated to phosphoserine and ISOLDE^75^, and then refined using real-space refinement in Phenix^76^ (Extended Data Fig. 5g,h). Cryo-EM data processing and model refinement statistics are summarized in Supplementary Table 1.

### Atomic modelling of ppS94-NME1 and pppS94-NME1

The atomic model of pS94-NME1 (see above) was used as a starting point. The model was first rigid-body fitted into the obtained sharpened density maps for ppS94-NME1 and oligo-pS94-NME1, respectively, using UCSF ChimeraX^73^, and then manually adjusted in Coot. Geometry restraints for ppS and pppS were generated using phenix.elbow^77^, and the resulting cif files were manually changed from a ligand to an amino acid. The changed amino acids were incorporated in the models, manually adjusted in Coot and then refined using real-space refinement in Phenix^76^ (Extended Data Figs. 6g,h and 7g,h). Cryo-EM data-processing and model-refinement statistics are summarized in Supplementary Table 1.

### Reporting summary

Further information on research design is available in the Nature Portfolio Reporting Summary linked to this article.

## Online content

Any methods, additional references, Nature Portfolio reporting summaries, source data, extended data, supplementary information, acknowledgements, peer review information; details of author contributions and competing interests; and statements of data and code availability are available at 10.1038/s41557-025-01915-8.

## Supplementary information

Supplementary Information Supplementary Figs. 1–28, Table 1, Methods, chemical synthesis and characterization, protein Q-TOF-MS spectra, and unprocessed blots and gels for supplementary figures.

Reporting Summary

Supplementary Table 2 LFQ values for wt-NME1 vs pS94-NME1 analysis. Supplementary Table 3 LFQ values for oligo-pS94-NME1 vs pS94-NME1 analysis. Supplementary Table 4 GO analysis. Supplementary Table 5 LFQ values for oligo-pS94-NME1 vs wt-NME1 analysis. Supplementary Table 6 Assessment of primary sequence motifs.

## Source data

Source Data Fig. 2b Numerical Source Data

Source Data Fig. 2c Unprocessed western blots

Source Data Fig. 3 Numerical Source Data

Source Data Fig. 4 Numerical Source Data

Source Data Extended Data Fig. 2 Numerical Source Data

Source Data Extended Data Fig. 3 Numerical Source Data

Source Data Extended Data Fig. 4 Numerical Source Data

Source Data Extended Data Fig. 5 Numerical Source Data

Source Data Extended Data Fig. 6 Numerical Source Data

Source Data Extended Data Fig. 7 Numerical Source Data

## Data Availability

Supporting figures and tables are available in the Supplementary Information. The MS proteomics data have been deposited in the ProteomeXchange Consortium via the PRIDE partner repository with the dataset identifier PXD054175^78^. Database for the human proteome: uniprot (https://www.uniprot.org/uniprotkb?query=Human). Database for NME1 phosphorylation sites: PhosphoSitePlus (https://www.phosphosite.org/-proteinAction.action?id=3836&showAllSites=true). The cryo-EM densities and resulting structural models of pS94-NME1, ppS94-NME1 and oligo-pS94-NME1 have been deposited in the EMDB and PDB under accession numbers 51248 and 9GD6, 51250 and 9GD8, and 51251 and 9GD9, respectively. Source data are provided with this paper.
