# Supplementary material for: Nucleoside diphosphate kinase A (NME1) catalyses its own oligophosphorylation
Source: Nat Chem. 2025 Aug 20;17(11):1757–67. doi: 10.1038/s41557-025-01915-8 (PMC12580328; doi:10.1038/s41557-025-01915-8)
Supplement: Supplementary file 1 — Supplementary Figs. 1–28, Table 1, Methods, chemical synthesis and characterization, protein Q-TOF-MS spectra, and unprocessed blots and gels for supplementary figures. [file 41557_2025_1915_MOESM1_ESM.pdf]

nature chemistry

Supplementary information

https://doi.org/10.1038/s41557-025-01915-8

Nucleoside diphosphate kinase A (NME1) catalyses its own oligophosphorylation

In the format provided by the authors and unedited

Table of Contents

Supplementary Figures 1–28

Supplementary Figure 1. Recombinant expression of wt-NME1.
Supplementary Figure 2. Recombinant expression of pT94-NME1.
Supplementary Figure 3. Recombinant expression of pS94-NME1.
Supplementary Figure 4. Refolding of wt-NME1 analyzed by SDS-PAGE.
Supplementary Figure 5. Assessment of NME1 secondary structure and activity before and after refolding.
Supplementary Figure 6. NDPK activity assay of H118-NME1 mutants.
Supplementary Figure 7. Autophosphorylation and hydrolysis of NME1 (wt, pS94, wt-H118F).
Supplementary Figure 8. Autophosphorylation and hydrolysis of pS94-NME1.
Supplementary Figure 9. Electron transfer no dissociation (ETnoD) event observed for the tryptic peptide containing pppS94.
Supplementary Figure 10. Screening different fragmentation techniques on oligophosphorylated NME1 peptide.
Supplementary Figure 11. NDPK activity assay of wt-NME1 in the absence and presence of aluminum or iron.
Supplementary Figure 12. NDPK activity assay of pS94-NME1 in the absence and presence of aluminum or iron.
Supplementary Figure 13. NDPK activity assay of ppS94-NME1 in the absence and presence of aluminum or iron.
Supplementary Figure 14. Analysis of expressed pT94-NME1 without further treatment by LC-MS/MS.
Supplementary Figure 15. Malachite Green phosphate detection assay with λ-phosphatase.
Supplementary Figure 16. LFQ analysis of endogenous T94-NME1 phosphorylation states.
Supplementary Figure 17. Fragmentation pattern evaluation of V[ox-M]LGE**T**NPADSKPGTIR and V[ox-M]LGE**T**NPADSKPATIR.
Supplementary Figure 18. Fragmentation pattern evaluation of V[ox-M]LGE[**pT**]NPADSKPGTIR and V[ox-M]LGE[**pT**]NPADSKPATIR.
Supplementary Figure 19. Fragmentation pattern evaluation of V[oxM]LGE[**pppT+Al^{3+}**]NPADSKPGTIR and V[oxM]LGE[**pppT+Al^{3+}**]NPADSKPATIR.
Supplementary Figure 20. LC-MS/MS signals of a dilution series of a standard peptide mixture.
Supplementary Figure 21. HCD MS/MS spectra of pT94 NME1 peptide after CDK1 treatment.
Supplementary Figure 22. Volcano plot depicting LFQ values of pS94-NME1 *versus* wt-NME1.
Supplementary Figure 23. Volcano plot depicting LFQ values of oligo-pS94-NME1 *versus* pS94-NME1.
Supplementary Figure 24. Malachite Green phosphate detection assay with alkaline phosphatase.
Supplementary Figure 25. Malachite Green phosphate detection assay with human inorganic pyrophosphatase (rhPPA1).
Supplementary Figure 26. Malachite Green phosphate detection assay with hPRUNE1.
Supplementary Figure 27. Malachite Green phosphate detection assay with hPRUNE1 in the presence of aluminum or iron.
Supplementary Figure 28. Crystal structure of PGAM5 highlighting the location of H105 and Tyr108

Supplementary Table 1

Supplementary Methods

Chemical Synthesis and Characterization

Protein Q-TOF-MS Spectra

Unprocessed blots and gels for Supplementary Figures

References

Supplementary Figures

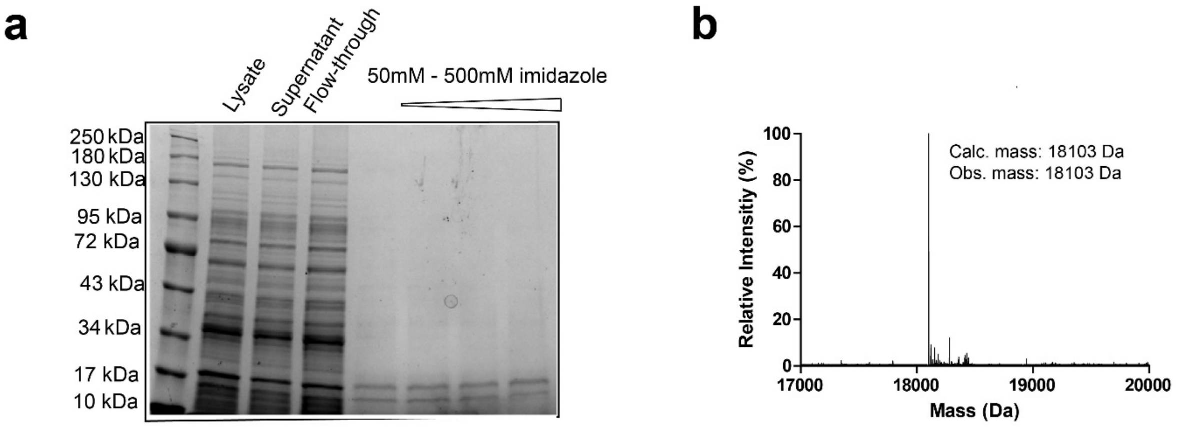

Supplementary Figure 1. Recombinant expression of wt-NME1. **a)** SDS-PAGE. Yield: 33 mg/L. **b)** Q-TOF-MS measurement.

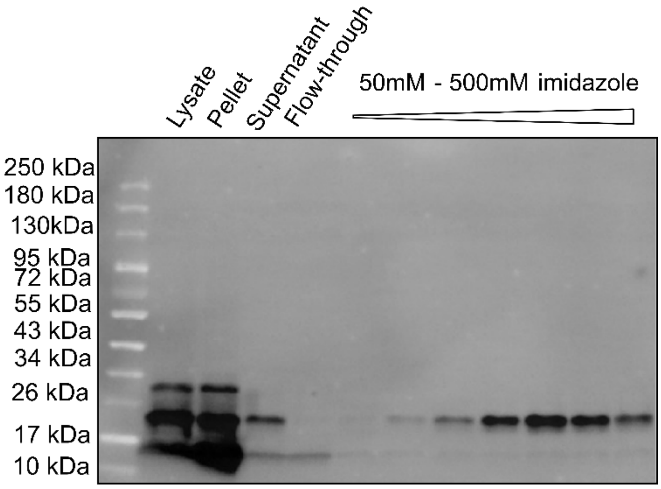

Supplementary Figure 2. Recombinant expression of pT94-NME1. Anti-His_6 Western blot (characterization by SDS-PAGE was not possible due to very low yield/concentration). Yield: 0.05 mg/L.

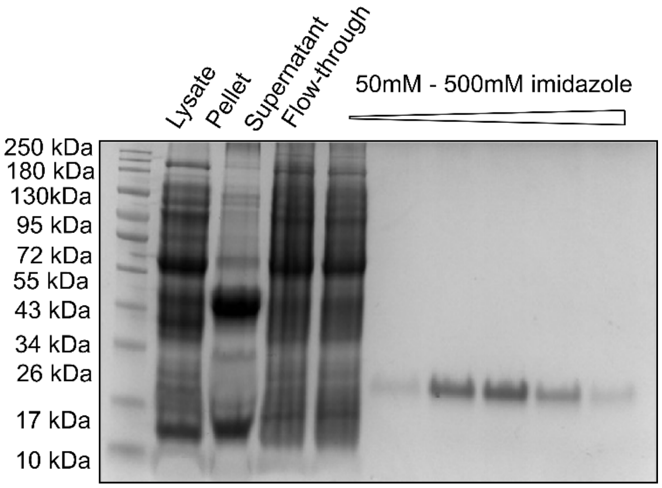

Supplementary Figure 3. Recombinant expression of pS94-NME1. **a)** SDS-PAGE. Yield: 0.75 mg/L.

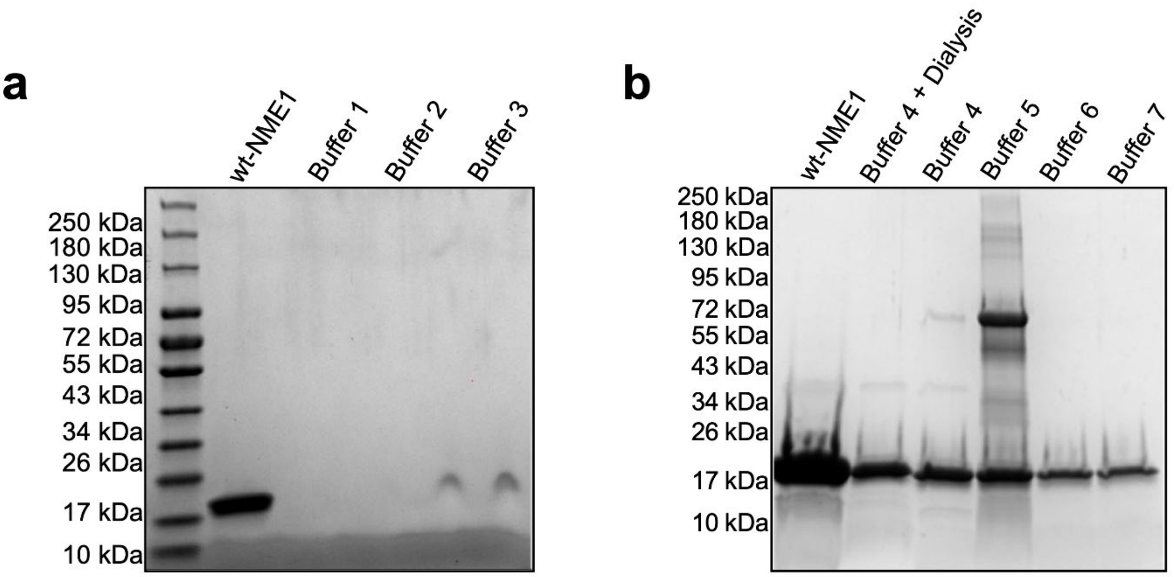

Supplementary Figure 4. Refolding of wt-NME1 analyzed by SDS-PAGE. **a)** SDS PAGE after applying the refolding protocol on wt-NME1. **b)** List of applied refolding buffers. **Buffer 1:** 50 mM Tris-HCl (pH 8.0), 35 mM KCl, 2 mM $MgCl_2$, 5 mM ADP, 100 mM L-Arg, 20 mM DTT. **Buffer 2:** 50 mM Tris-HCl (pH 8.0), 250 mM KCl, 2 mM $MgCl_2$, 5 mM ADP, 100 mM L-Arg, 20 mM DTT. **Buffer 3:** 50 mM Tris-HCl (pH 8.0), 500 mM NaCl, 50 mM $MgCl_2$, 50 mM L-Arg, 50 mM L-Glu, 5% glycerin. **Buffer 4**: 50 mM Tris-HCl (pH 8.5), 35 mM KCl, 0.3 mM GSSG, 3 mM GSH, 10 mM EDTA, 0.2% CHAPS. **Buffer 5**: 50 mM Tris-HCl (pH 8.5), 35 mM KCl, 20 mM DTT, 10 mM EDTA, 0.2% CHAPS. **Buffer 6**: 50 mM Tris-HCl (pH 8.5), 35 mM KCl, 0.3 mM GSSG, 3 mM GSH, 10 mM EDTA, 0.2% CHAPS, 400 mMsucrose. **Buffer 7**: 50 mM Tris-HCl (pH 8.5), 35 mM KCl, 0.3 mM GSSG, 3 mM GSH, 10 mM EDTA, 0.2% CHAPS, 500 mM L-Arg.

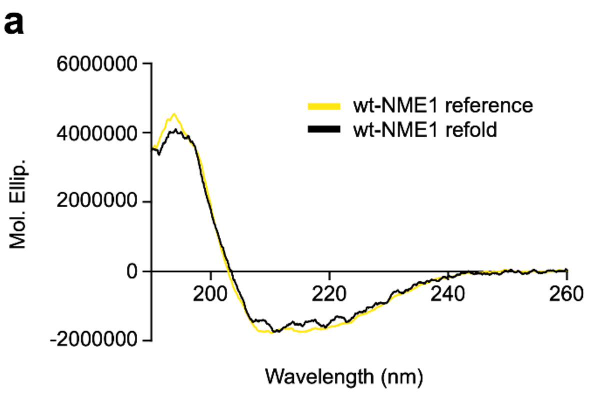

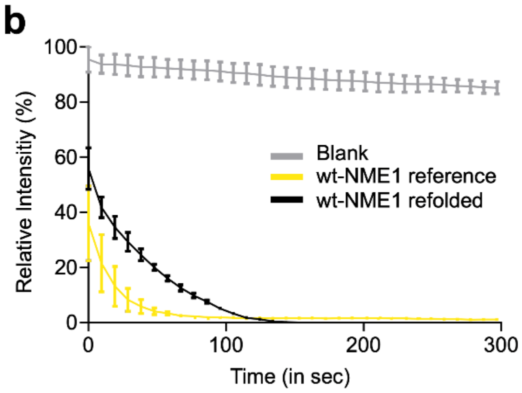

Supplementary Figure 5. Assessment of NME1 secondary structure and activity before and after refolding. **a)** CD-spectra of wt-NME1 before (in yellow) and after refolding (in black). **b)** Nucleoside diphosphate kinase activity before and after refolding monitored by NADH consumption. Assay condition: Purified NME1 (10 nM) was added to a reaction mixture containing 50 mM HEPES, 2 mM ATP, 2.2 mM ATP, 0.2 mM β-NADH, 1.1 mM PEP, 10 mM $MgCl_2$, 10 units lactic dehydrogenase, and 7 units pyruvate kinase to a final volume of 100 µL. The reaction was vortexed immediately and a decrease in absorbance of NADH at 340 nm was subsequently recorded for 5 min. UV signals were read out with a Tecan Infinite M Plex plate reader. Data is presented as mean ± SD of three technical replicates.

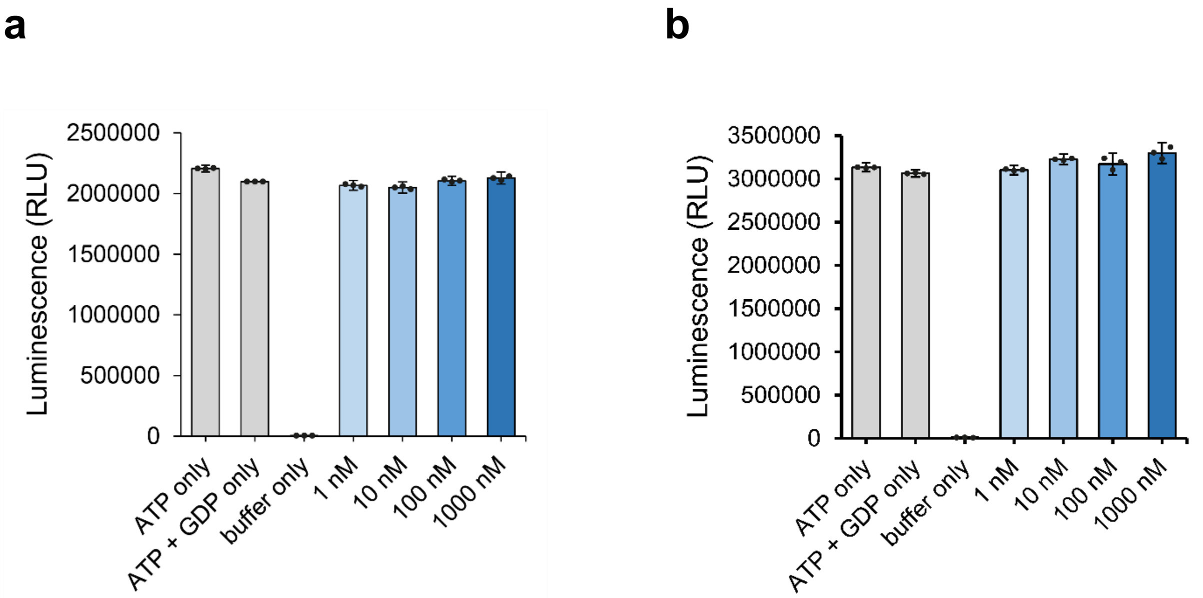

Supplementary Figure 6. NDPK activity assay of H118-NME1 mutants. **a)** T94pS-H118F-NME1, and **b)** H118F-NME1. Kinase activity was measured utilizing GDP as a substrate, at 37 °C for 1 h in 50 mM Tris-HCl (pH 8.0), 150 mM NaCl, 10 mM $MgCl_2$, 900 µM GDP, and 100 µM ATP. Data is presented as mean ± SE of three technical replicates.

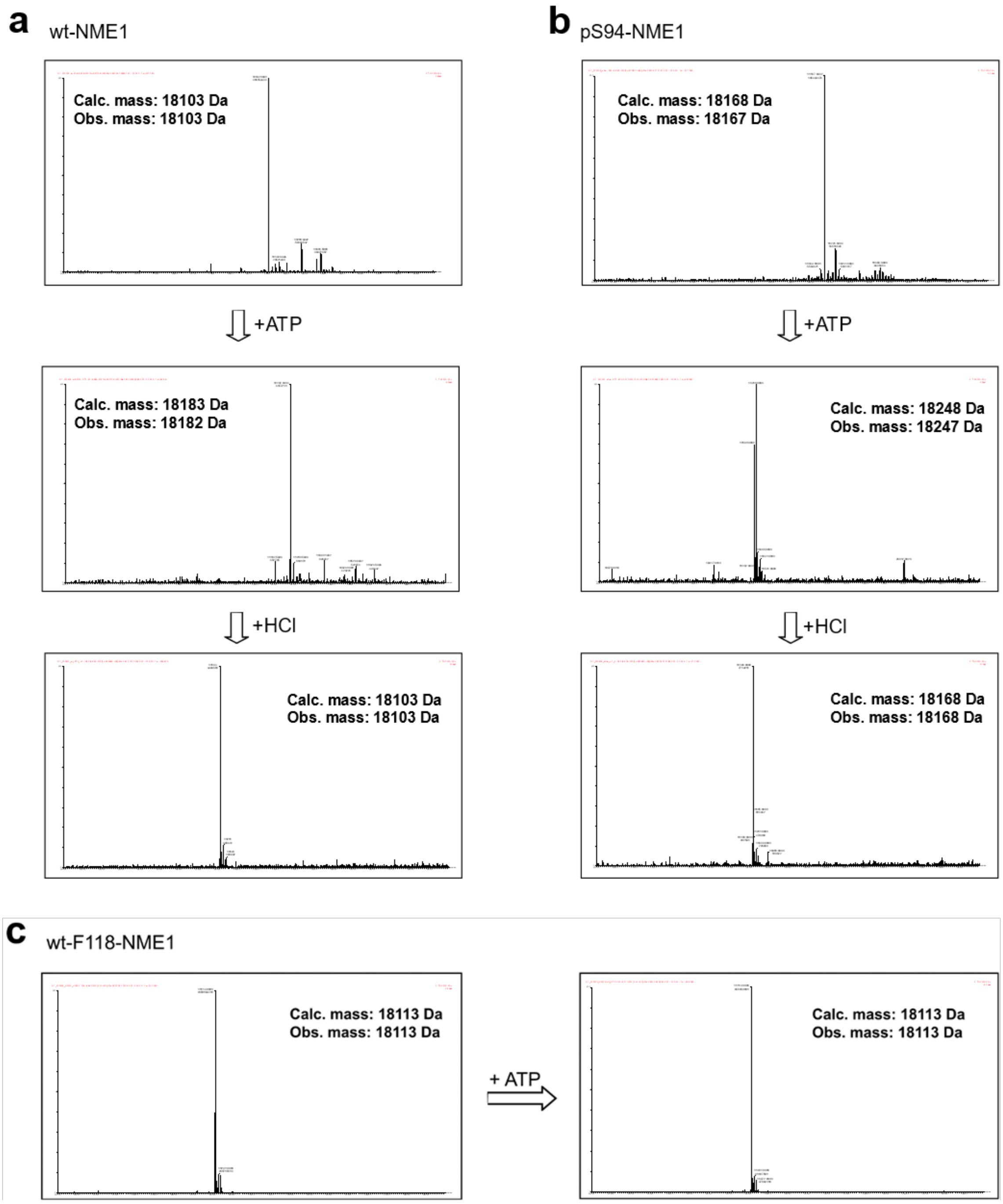

Supplementary Figure 7. Autophosphorylation and hydrolysis of NME1 (wt, pS94, wt-H118F). **a)** wt-NME1 treated with ATP (1 mM) and subsequently with HCl (1 M) monitored by Q-TOF-MS. **b)** pS94-NME1 treated with ATP (1 mM) and subsequently with HCl (1 M) monitored by Q-TOF-MS. **c)** H118F-NME1 treated with ATP (1 mM) monitored by Q-TOF-MS.

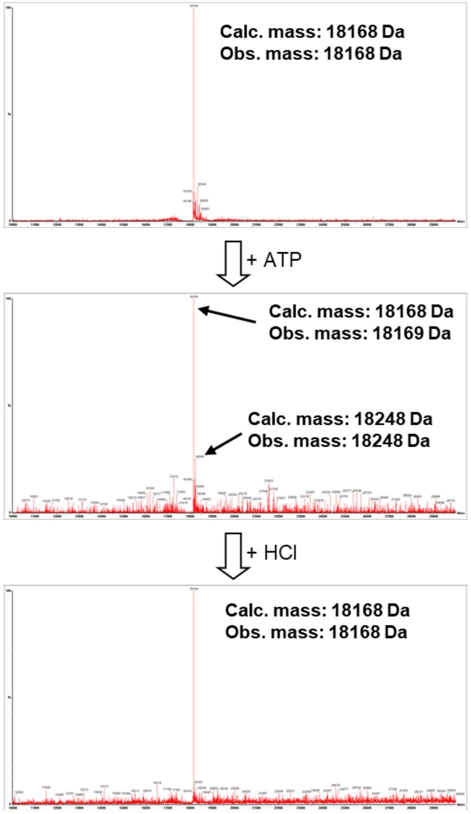

Supplementary Figure 8. Autophosphorylation and hydrolysis of pS94-NME1. Treatment with ATP (200 μM) in TBS buffer (50 mM Tris-HCl pH 7.5, 150 mM NaCl, PhosStop™, cOmplete™ Mini EDTA-free protease inhibitor cocktail) for 3 h at 4 °C, followed by HCl (1 M) for 1 h at 37 °C monitored by Q-TOF-MS. To determine the concentration of ATP in HEK293T cell lysate, ATP-Glo assay was performed as described in the manufacturer's protocol.

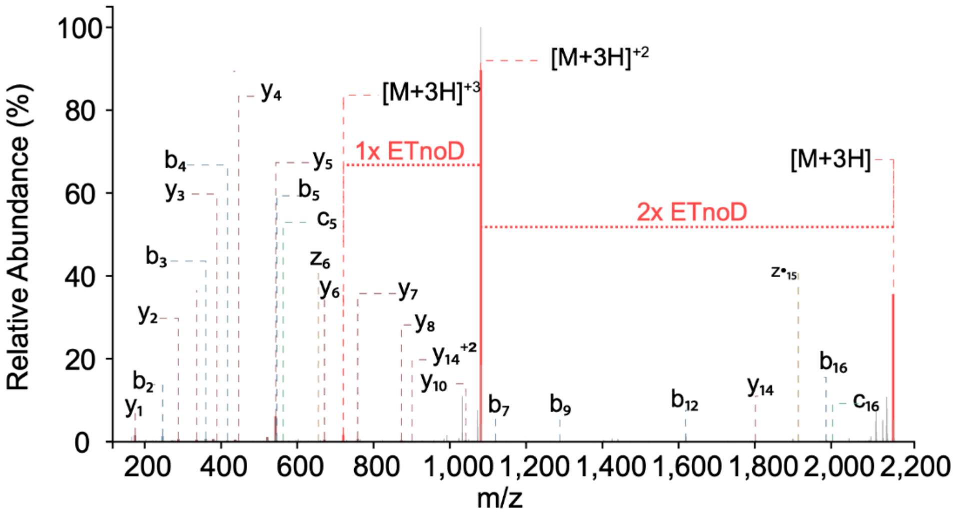

Supplementary Figure 9. Electron transfer no dissociation (ETnoD) event observed for the tryptic peptide containing pppS94. The measurement was performed on an Orbitrap Fusion instrument.

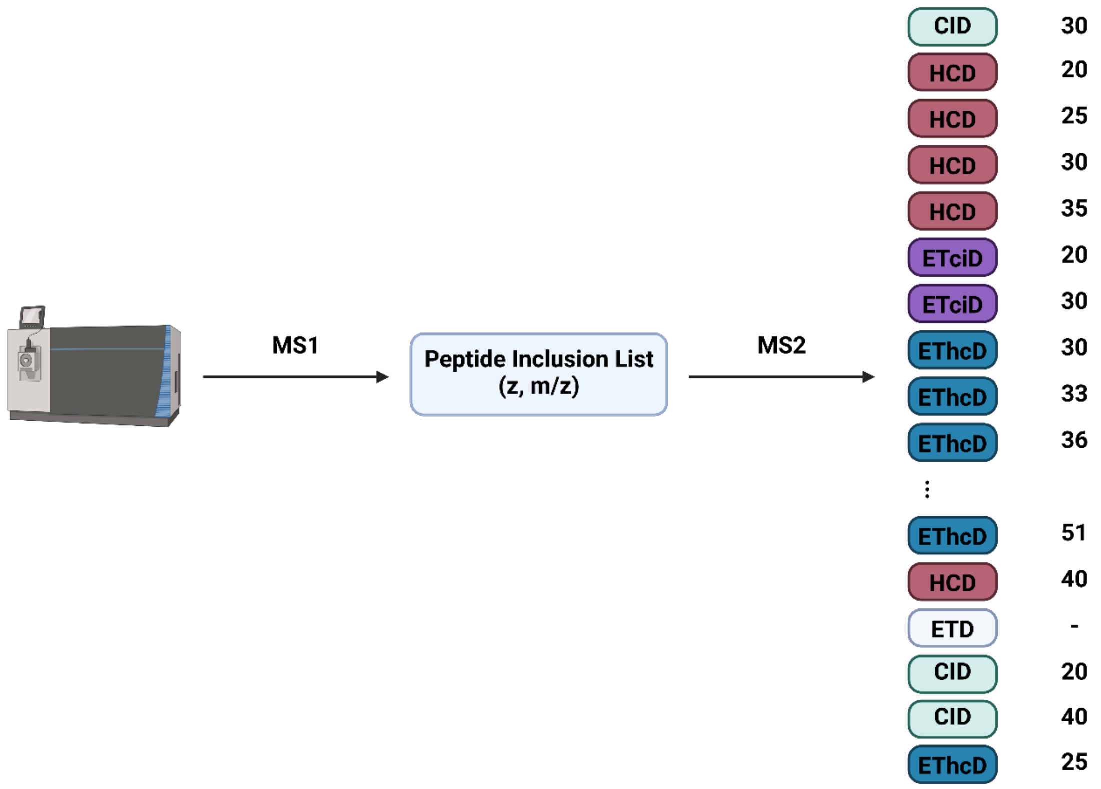

Supplementary Figure 10. Screening different fragmentation techniques on oligophosphorylated NME1 peptide.

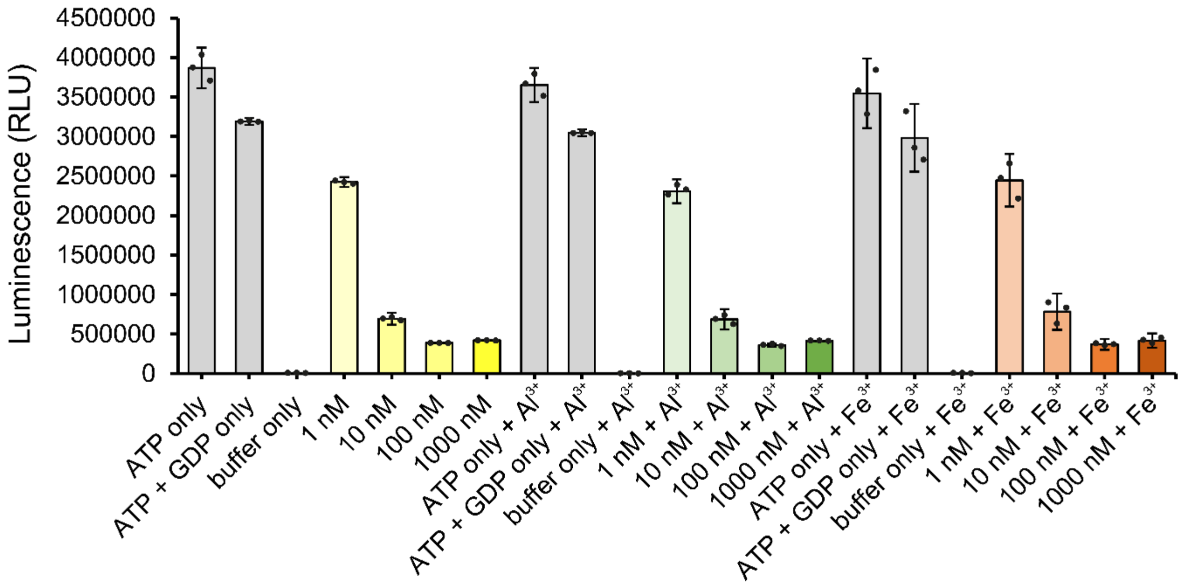

Supplementary Figure 11. NDPK activity assay of wt-NME1 in the absence (yellow) and presence of aluminum (Al, green) or iron (Fe, brown). Kinase activity was measured utilizing GDP as a substrate, at 37 °C for 1 h in 50 mM Tris-HCl (pH 8.0), 150 mM NaCl, 10 mM $MgCl_2$, 900 µM GDP, 100 µM ATP, and 100 µM $Al_2(SO_4)_3$ or $Fe_2(SO_4)_3$. Data is presented as mean ± SE of three technical replicates.

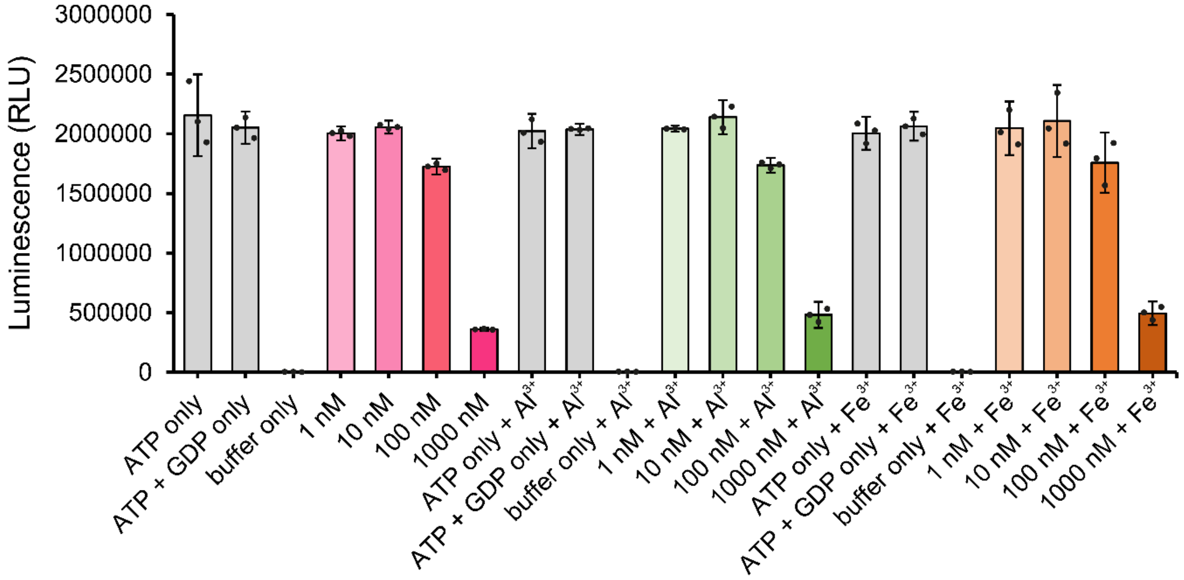

Supplementary Figure 12. NDPK activity assay of pS94-NME1 in the absence (pink) and presence of aluminum (Al, green) or iron (Fe, brown). Kinase activity was measured utilizing GDP as a substrate, at 37 °C for 1 h in 50 mM Tris-HCl (pH 8.0), 150 mM NaCl, 10 mM $MgCl_2$, 900 μM GDP, 100 μM ATP, and 100 μM $Al_2(SO_4)_3$ or $Fe_2(SO_4)_3$. Data is presented as mean ± SE of three technical replicates.

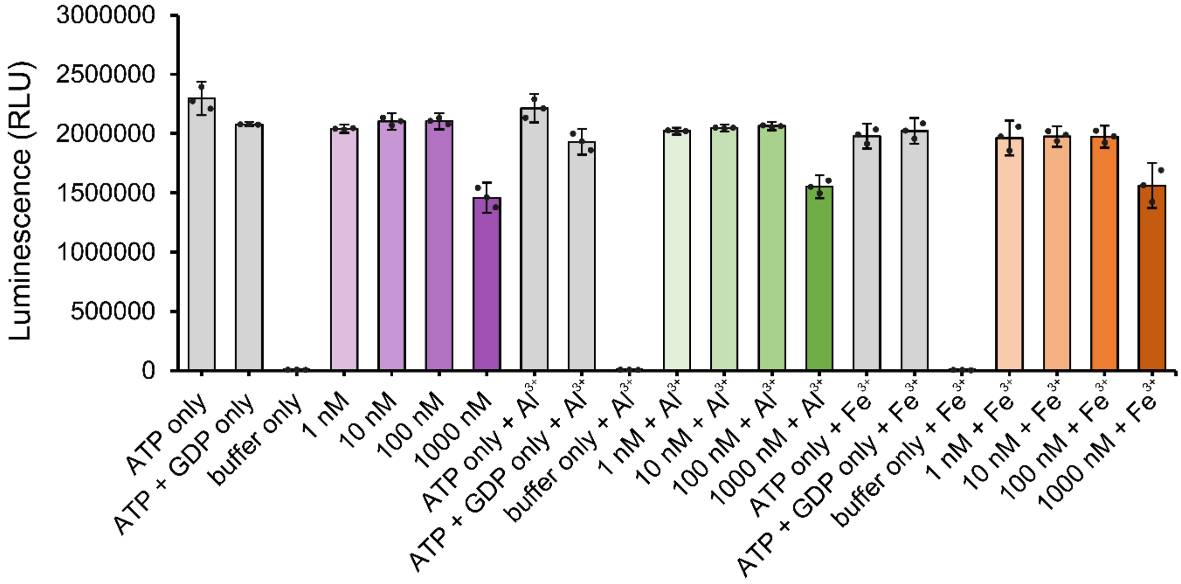

Supplementary Figure 13. NDPK activity assay of ppS94-NME1 in the absence (violet) and presence of aluminum (Al, green) or iron (Fe, brown). Kinase activity was measured utilizing GDP as a substrate, at 37 °C for 1 h in 50 mM Tris-HCl (pH 8.0), 150 mM NaCl, 10 mM $MgCl_2$, 900 μM GDP, 100 μM ATP, and 100 μM $Al_2(SO_4)_3$ or $Fe_2(SO_4)_3$. Data is presented as mean ± SE of three technical replicates.

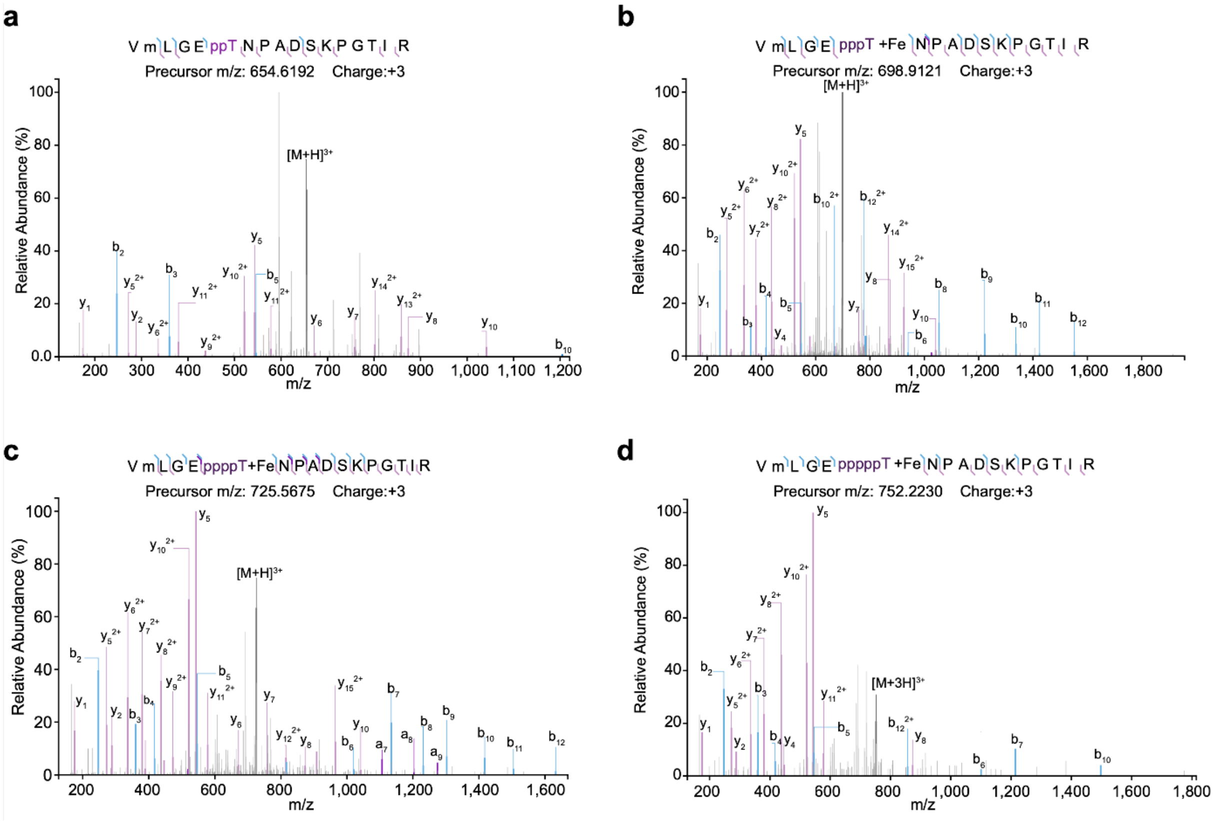

Supplementary Figure 14. Analysis of expressed pT94-NME1 without further treatment by LC-MS/MS. Stepped-HCD MS/MS spectra of pyrophosphorylated and oligophosphorylated NME1 (expressed as pT94NME1) localizing **a)** pyrophosphorylation, **b)** triphosphorylation, **c)** tetraphosphorylation, and **d)** pentaphosphorylation on T94.

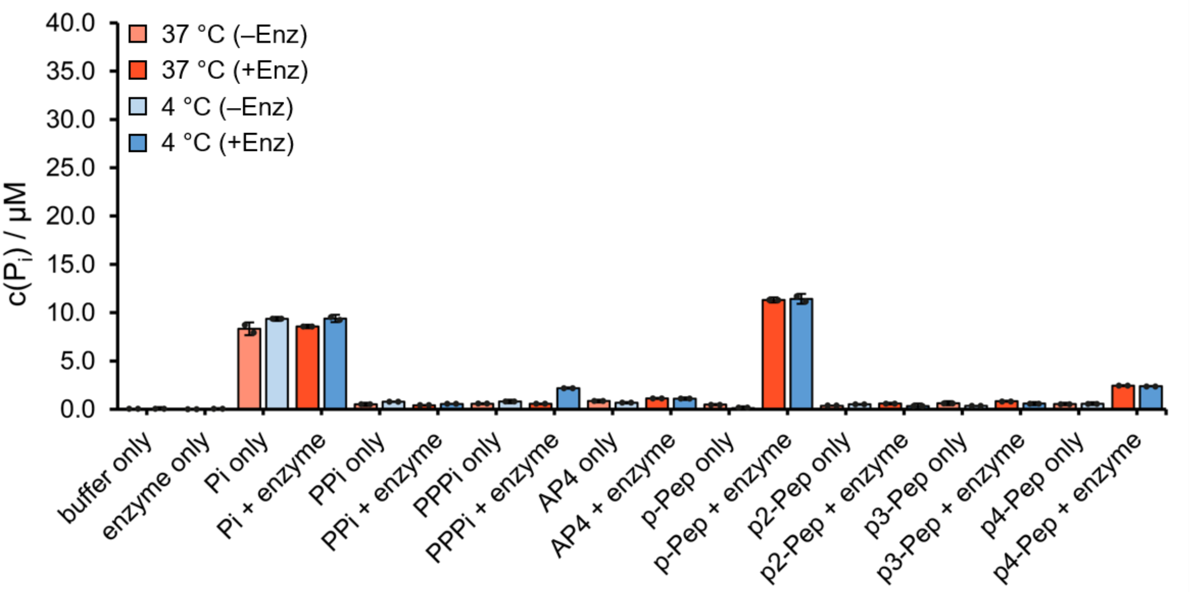

Supplementary Figure 15. Malachite Green phosphate detection assay with λ-phosphatase. Phosphatase activity was measured after incubation at 37 °C for 1 h (red) or at 4 °C for 3 h (blue) in PMP buffer (50 mM HEPES (pH 7.5), 100 mM NaCl, 2 mM DTT, 1 mM $MnCl_2$, 0.01% Brij 35), 100 U λ-phosphatase, and 10 µM phosphate containing species. P_i = HPO_4^{2-}, PP_i = $Na_4P_2O_7$, PPP_i = $Na_5P_3O_{10}$, AP_4 = adenosine 5′-tetraphosphate, p-Pep = VMLGE[pT]NPADSKPATIR, p_2-Pep = VMLGE[ppT]NPADSKPATIR, p_3-Pep = VMLGE[pppT]NPADSKPATIR, p_4-Pep = VMLGE[ppppT]NPADSKPATIR. Data is presented as mean ± SE of two technical replicates.

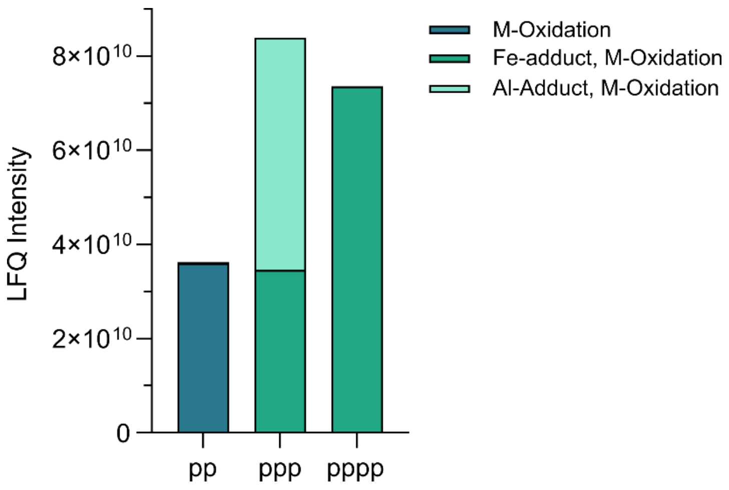

Supplementary Figure 16. LFQ analysis of endogenous T94-NME1 phosphorylation states. The dataset of the enriched HEK293T lysate fractions, which was also used for endogenous oligophosphorylation identification (Fig. 3e-f), was analyzed. The MaxLFQ intensities of different adducts of a phospho-species, as well as the intensities in the respective fractions due to the occurrence of a species in several fractions, were summed. Although the LFQ comparison of different phospho-species is not optimal due to their different MS response (caused by ionization and chromatographic behavior, etc.), the results indicate a considerable endogenous relevance of the oligophosphorylation of NME1. Validation experiments showed, as expected, a lower MS response for the oligophosphorylated peptides (**Supplementary Fig. 20, Extended Data Fig. 4d**).

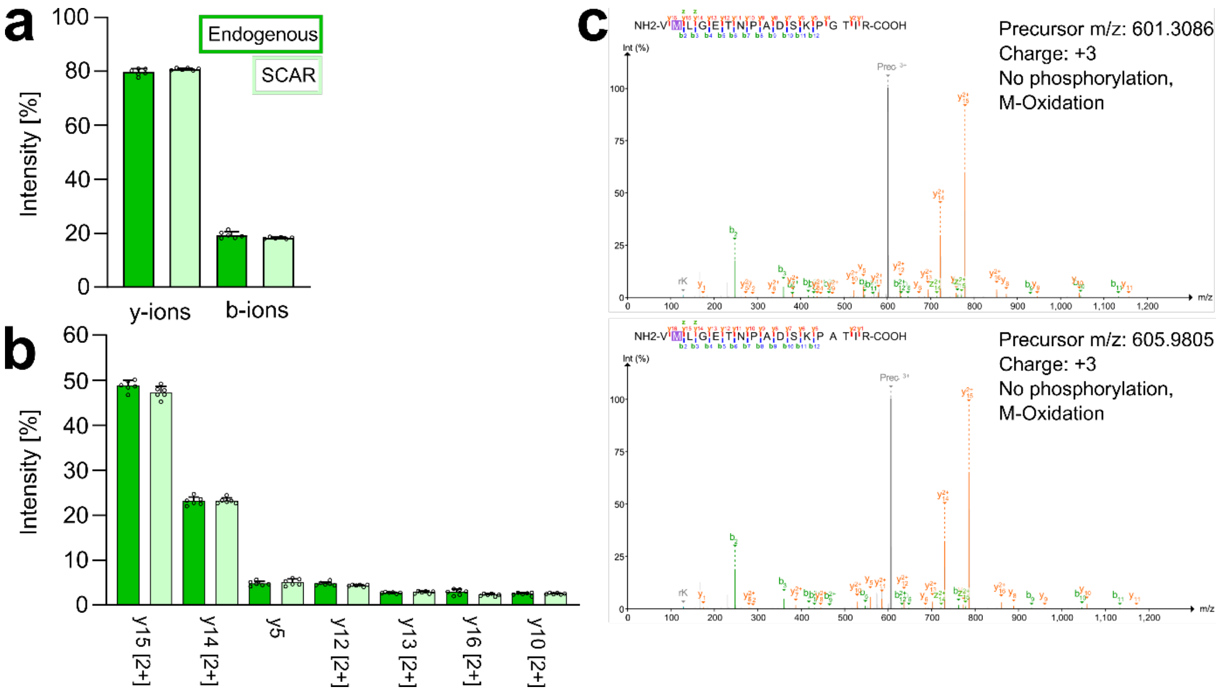

Supplementary Figure 17. Fragmentation pattern evaluation of V[ox-M]LGETNPADSKPGTIR and V[ox-M]LGETNPADSKPATIR. A standard peptide mixture containing 2 pmol of standard peptides of the endogenous and the SCAR sequences respectively was measured on Orbitrap Lumos using stepped HCD (20–23–26). **a)** C– and N–terminal charge retention using y- and b-ion signals **b)** Relative intensities of the most abundant y-ion transitions were calculated as the percentage of the total y-ion signal (mean ± SD, n = 6 spectra, t-test indicates no significant differences between endogenous and SCAR signals – P < 0.05 is considered significant). **c)** MS/MS spectra of the standard peptides.

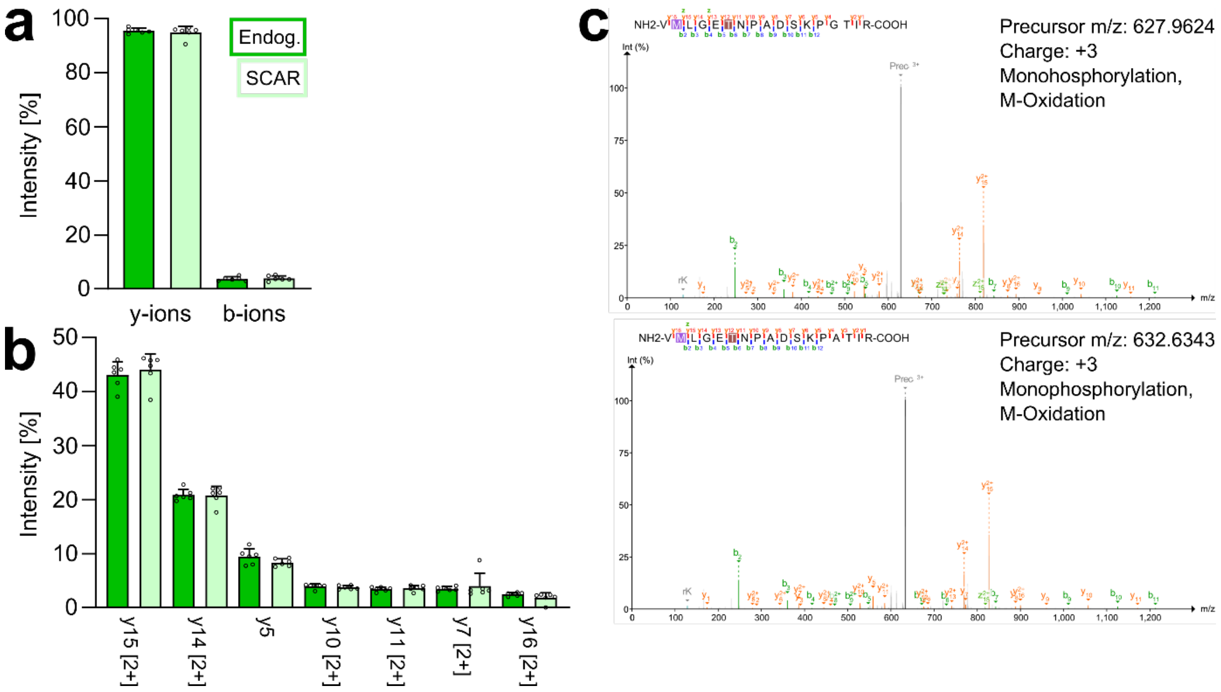

Supplementary Figure 18. Fragmentation pattern evaluation of V[ox-M]LGE[pT]NPADSKPGTIR and V[ox-M]LGE[pT]NPADSKPATIR. A standard peptide mixture containing 2 pmol of standard peptides of the endogenous and the SCAR sequences respectively was measured on Orbitrap Lumos using stepped HCD (20–23–26). **a)** C– and N–terminal charge retention using y- and b-ion signals. **b)** Relative intensities of the most abundant y-ion transitions were calculated as the percentage of the total y-ion signal (mean ± SD, n = 6 spectra, t-test indicates no significant differences between endogenous and SCAR signals – $P < 0.05$ is considered significant). **c)** MS/MS spectra of the standard peptides.

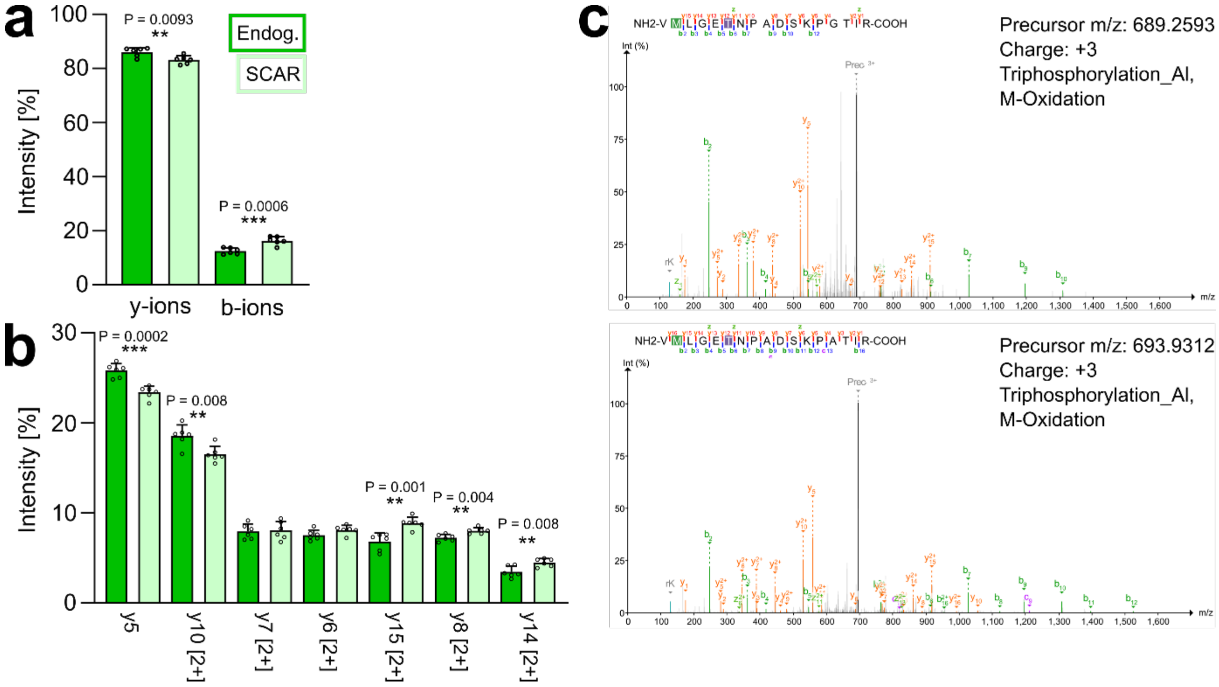

Supplementary Figure 19. Fragmentation pattern evaluation of V[oxM]LGE[**pppT+Al^{3+}**]NPADSKPGTIR and V[oxM]LGE[**pppT+Al^{3+}**]NPADSKPATIR. A standard peptide mixture containing 2 pmol of standard peptides of the endogenous and the SCAR sequences respectively was measured on Orbitrap Lumos using stepped HCD (20–23–26). **a)** C– and N–terminal charge retention using y- and b-ion signals. **b)** Relative intensities of the most abundant y-ion transitions were calculated as the percentage of the total y-ion signal (mean ± SD, n = 6 spectra, for several transitions a t-test indicates significant differences between endogenous and SCAR signals – P < 0.05 is considered significant). **c)** MS/MS spectra of the standard peptides.

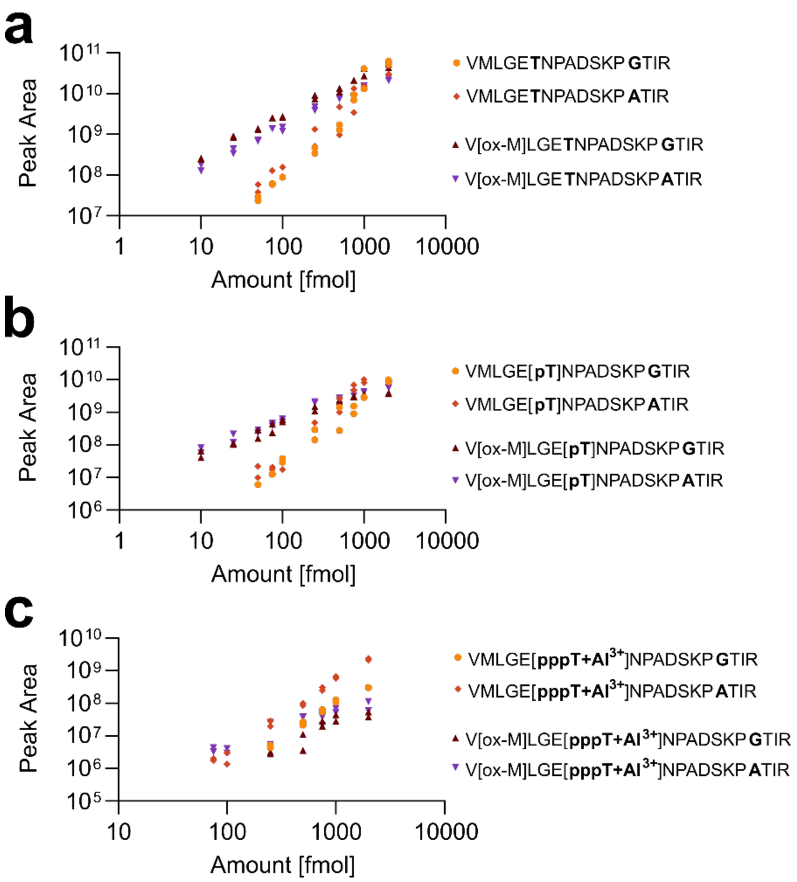

Supplementary Figure 20. LC-MS/MS signals of a dilution series of a standard peptide mixture. Injection of 10 fmol, 25 fmol, 50 fmol, 75 fmol, 100 fmol, 250 fmol, 500 fmol, 750 fmol, 1 pmol, and 2 pmol. After synthesis, the peptides were partly present with M-oxidation and partly without. The peptide species/adducts that were detected and used for absolute quantification after corresponding enrichment are plotted (**Extended Data Fig. 4e**). The samples were measured with stepped-HCD (20–23–26) on an Orbitrap Lumos instrument using PRM, targeting all possible peptide species, including iron/aluminum adducts with and without oxidation of the methionine. Data is presented as mean ± SD of technical duplicates.

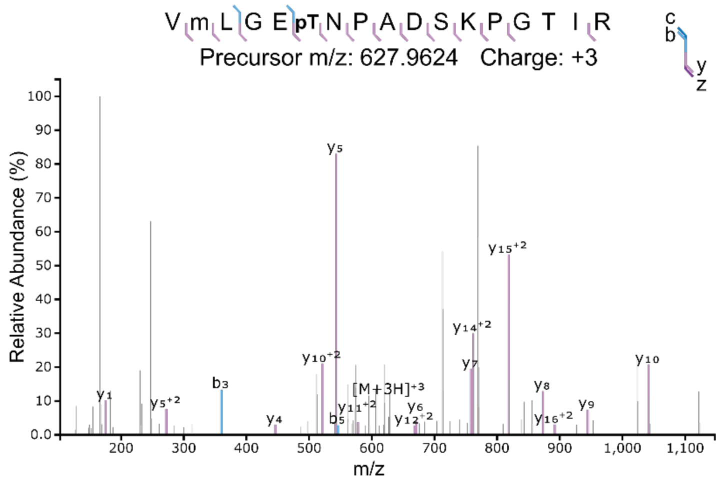

Supplementary Figure 21. HCD MS/MS spectra of pT94 NME1 peptide after CDK1 treatment. Measured on an Orbitrap Fusion using HCD fragmentation.

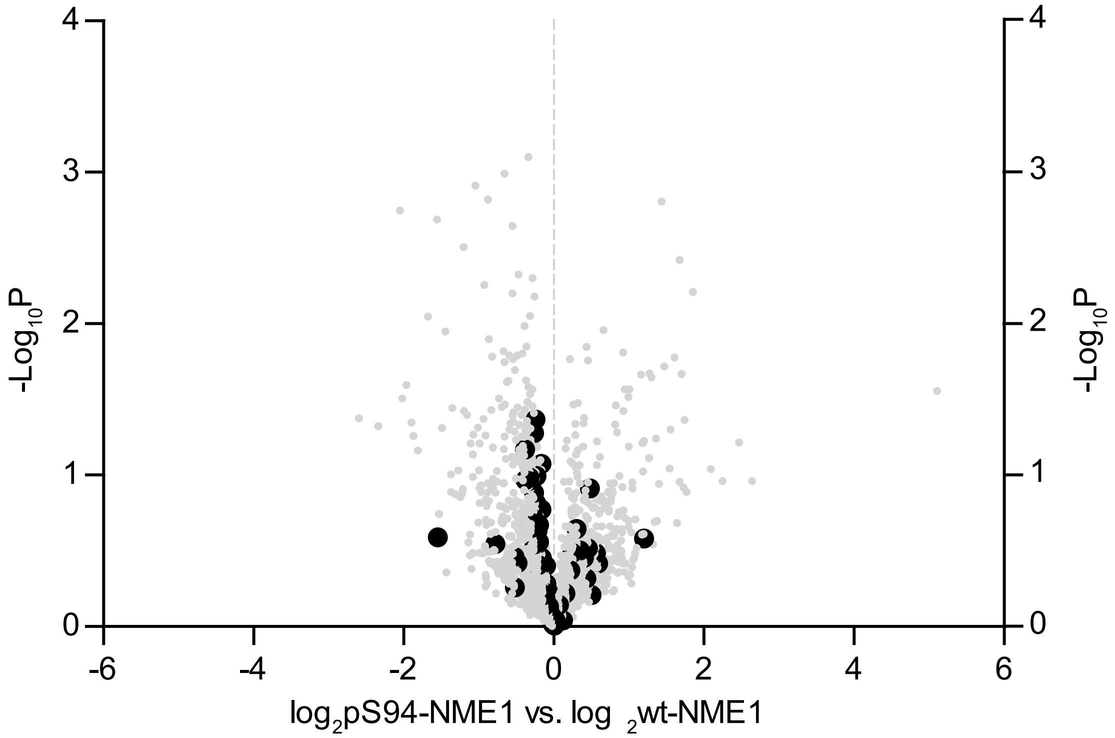

Supplementary Figure 22. Volcano plot depicting LFQ values of pS94-NME1 *versus* wt-NME1 after a t-test. The x-axis displays the difference of LFQ values on a $\log_2$ scale and the y-axis shows the $-\log_{10}P$ value. Labeled hits (large black dots) represent known interactors (BioGRID database) preferentially enriched with wt-NME1 or pS94-NME1.

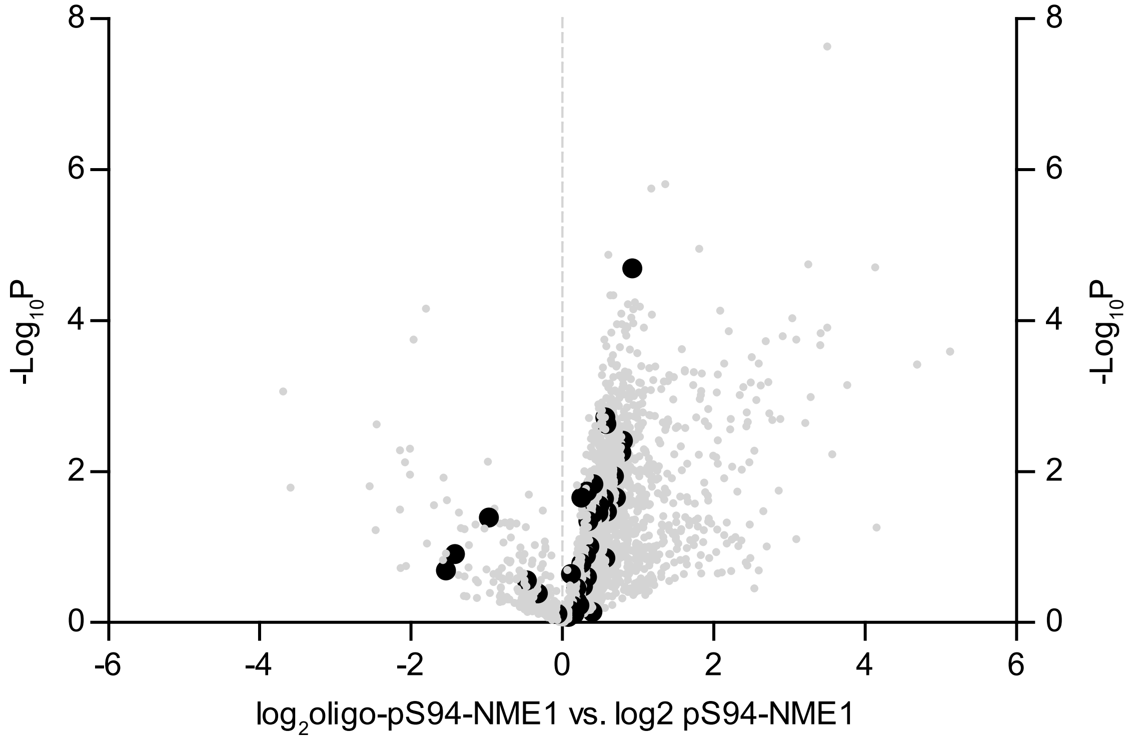

Supplementary Figure 23. Volcano plot depicting LFQ values of oligo-pS94-NME1 *versus* pS94-NME1 after a t-test. The x-axis displays the difference of LFQ values on a log_2 scale and the y-axis shows the $-log_{10}P$ value. Labeled hits (large black dots) represent known interactors (BioGRID database) preferentially enriched with pS94-NME1 or oligo-pS94-NME1.

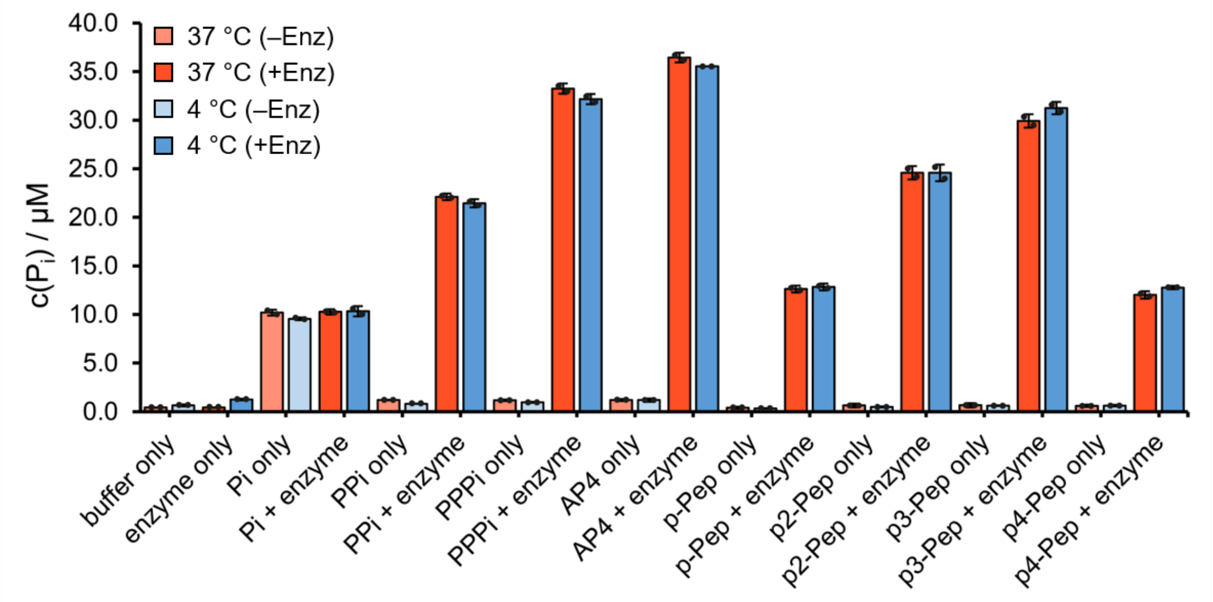

Supplementary Figure 24. Malachite Green phosphate detection assay with alkaline phosphatase (FastAP™). Phosphatase activity was measured after incubation at 37 °C for 1 h (red) or at 4 °C for 3 h (blue) in FastAP™ buffer (10 mM Tris-HCl (pH 8.0), 100 mM KCl, 5 mM $MgCl_2$, 0.02% Triton X-100, 0.1 mg/mL BSA), 1 U FastAP™, and 10 µM phosphate containing species. P_i = HPO_4^{2-}, PP_i = $Na_4P_2O_7$, PPP_i = $Na_5P_3O_{10}$, AP_4 = adenosine 5′-tetraphosphate, p-Pep = VMLGE[pT]NPADSKPATIR, p_2-Pep = VMLGE[ppT]NPADSKPATIR, p_3-Pep = VMLGE[pppT]NPADSKPATIR, p_4-Pep = VMLGE[ppppT]NPADSKPATIR. Data is presented as mean ± SE of two technical replicates.

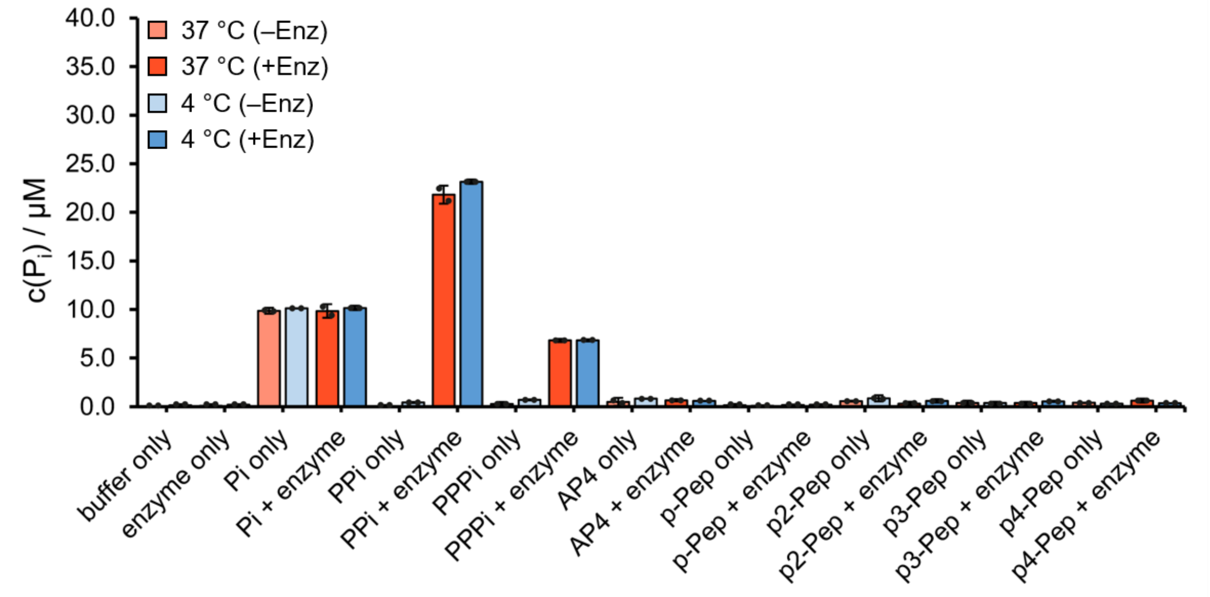

Supplementary Figure 25. Malachite Green phosphate detection assay with human inorganic pyrophosphatase (rhPPA1). Phosphatase activity was measured after incubation at 37 °C for 1 h (red) or at 4 °C for 3 h (blue) in 50 mM Tris-HCl (pH 8.0), 150 mM NaCl, 10 mM $MgCl_2$, 5.4 nM hPPA1, and 10 µM phosphate containing species. P_i = HPO_4^{2-}, PP_i = $Na_4P_2O_7$, PPP_i = $Na_5P_3O_{10}$, AP_4 = adenosine 5′-tetraphosphate, p-Pep = VMLGE[pT]NPADSKPATIR, p_2-Pep = VMLGE[ppT]NPADSKPATIR, p_3-Pep = VMLGE[pppT]NPADSKPATIR, p_4-Pep = VMLGE[ppppT]NPADSKPATIR. Data is presented as mean ± SE of two technical replicates.

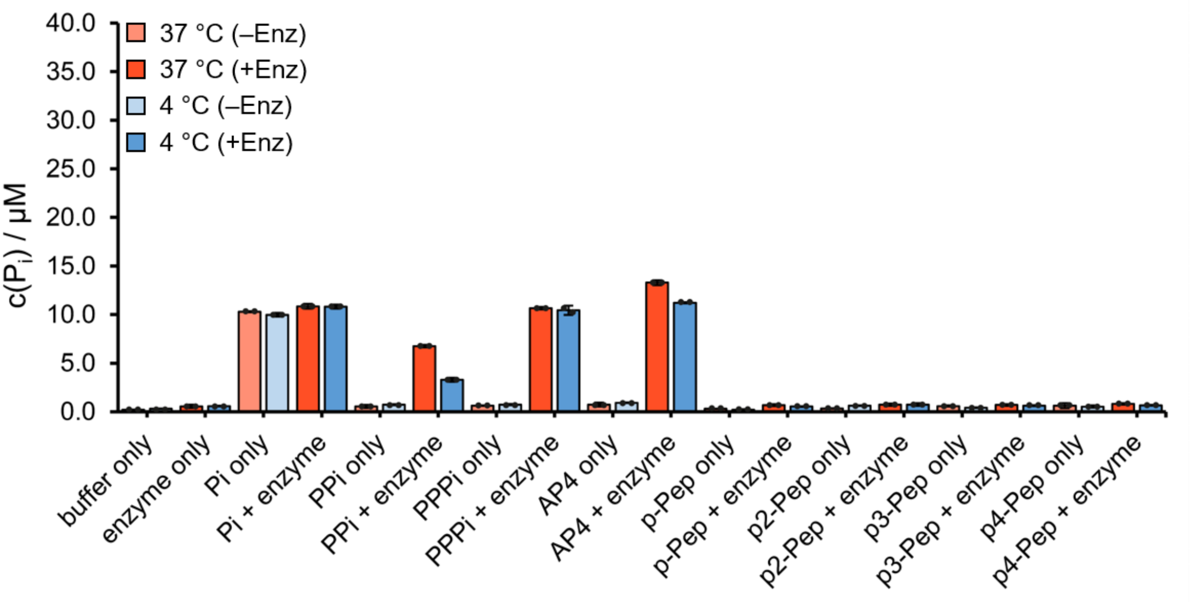

Supplementary Figure 26. Malachite Green phosphate detection assay with hPRUNE1. Phosphatase activity was measured after incubation at 37 °C for 1 h (red) or at 4 °C for 3 h (blue) in 50 mM Tris-HCl (pH 8.0), 150 mM NaCl, 10 mM $MgCl_2$, 200 nM hPRUNE, and 10 µM phosphate containing species. P_i = HPO_4^{2-}, PP_i = $Na_4P_2O_7$, PPP_i = $Na_5P_3O_{10}$, AP_4 = adenosine 5′-tetraphosphate, p-Pep = VMLGE[pT]NPADSKPATIR, p_2-Pep = VMLGE[ppT]NPADSKPATIR, p_3-Pep = VMLGE[pppT]NPADSKPATIR, p_4-Pep = VMLGE[ppppT]NPADSKPATIR. Data is presented as mean ± SE of two technical replicates.

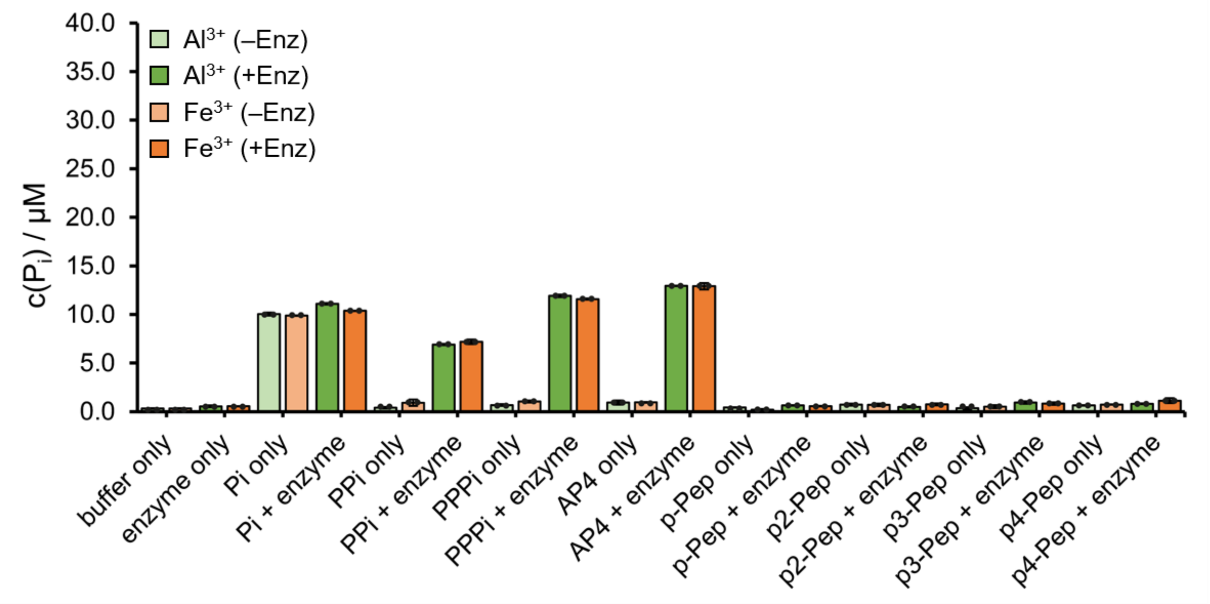

Supplementary Figure 27. Malachite Green phosphate detection assay with hPRUNE1 in the presence of aluminum (green) or iron (brown). Phosphatase activity was measured after incubation at 37 °C for 1 h in 50 mM Tris-HCl (pH 8.0), 150 mM NaCl, 10 mM $MgCl_2$, 10 µM $Al_2(SO_4)_3$ or $Fe_2(SO_4)_3$, 200 nM hPRUNE, and 10 µM phosphate containing species. P_i = HPO_4^{2-}, PP_i = $Na_4P_2O_7$, PPP_i = $Na_5P_3O_{10}$, AP_4 = adenosine 5′-tetraphosphate, p-Pep = VMLGE[pT]NPADSKPATIR, p_2-Pep = VMLGE[ppT]NPADSKPATIR, p_3-Pep = VMLGE[pppT]NPADSKPATIR, p_4-Pep = VMLGE[ppppT]NPADSKPATIR. Data is presented as mean ± SE of two technical replicates.

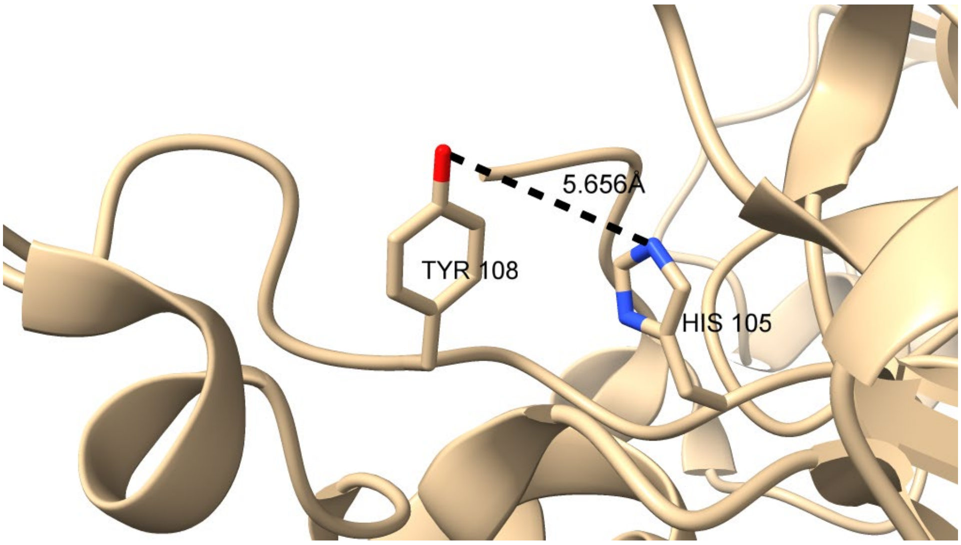

Supplementary Figure 28. Crystal structure of PGAM5 highlighting the location of H105 and Tyr108 (PDB code: 3MXO).

Supplementary Tables

Supplementary Table 1: Cryo-EM data collection, refinement, and model validation statistics.

**Data collection and processing**	**pS94-NME1**<br>**PDB 9GD6**	**ppS94-NME1**<br>**PDB 9GD8**	**oligo-pS94-NME1**<br>**PDB 9GD9**
Magnification	105,000		
Voltage (kV)	300		
Camera	Gatan K3 with energy filter		
Electron exposure (e–/Å^2)	47.7	44.6	44.1
Defocus range (μm)	-1.1 – 2.6	-1.2 – 2.4	-1.4 – 2.4
Pixel size (Å)	0.83		
Micrographs used	3190	4639	4856
Total extracted particle images	3,559,391	1,546,555	2,121,197
Refined particle images	1,038,113	780,780	507,420
Final particle images	532,096	486,617	201,312
Map resolution (Å)	2.8	3.3	3.8
FSC threshold	0.143	0.143	0.143
Map resolution range (Å)	2.5 – 3.5	2.5 – 3.5	3.0 - 4.6
**Refinement**			
Refinement package	Phenix 1.20.1-4487	Phenix 1.21-5207	Phenix 1.21-5207
Model resolution (Å)	3.0	3.9	4.5
FSC threshold	0.5	0.5	0.5
Map sharpening *B* fac (Å^2)	-182.2	-239.4	-258.1
Model composition			
Non-hydrogen atoms	7116	6648	6744
Protein residues	894	834	840
Ligands	0	6	6
B factors (Å^2)			
Protein	62.92	187.03	240.54
Ligand		206.27	257.59
R.m.s. deviations			
Bond lengths (Å)	0.003	0.002	0.002
Bond angles (°)	0.658	0.514	0.521
Validation			
MolProbity score	1.84	2.69	2.54
Clashscore	10.82	14.22	12.94
Poor rotamers (%)	2.38	7.69	4.24
Ramachandran plot			
Favored (%)	98.15	94.81	93.14
Allowed (%)	1.16	5.19	6.86
Disallowed (%)	0.69	0	0

Supplementary Methods

Q-TOF-MS

High-resolution ESI-MS spectra were recorded on two different instruments: 1) Agilent 6220 TOF Accurate Mass coupled to an Agilent 1200 LC (Agilent Technologies, USA) and were measured at 35 ˚C between 100–2000 m/z. The used column was an Accucore RP-MS (30 x 2.1 mm; 2.6 µm particle size) eluted with a flow of 0.8 mL/min and the following gradient (A = H_2O + 0.1% TFA, B = MeCN + 0.1% TFA), gradient: 5% B 0–0.2 min, 5–99% B 0.2–1.1 min, 99% B 1.1–2.5 min. 2) Agilent Technologies 6230 Accurate Mass TOF LC/MS linked to Agilent Technologies HPLC 1260 Series; Column: Thermo Accucore RP-MS; Particle Size: 2.6 µM; Dimension: 30 x 2.1 mm. The following gradient was used: A = H_2O + 0.1 % formic acid, B = MeCN + 0.1 % formic acid, 5% B 0.0–0.2 min, 5-99% B 0.2–1.1 min, 99% B 1.1–3.6 min, 5% B 3.6–4.9 min. Flow rate: 0.8 mL/min; UV-detection: 220 nm, 254 nm, 300 nm.

Intact protein MS

Intact proteins were analyzed using a *Waters* H-class instrument equipped with a quaternary solvent manager, a Waters sample manager-FTN, a Waters PDA detector, and a Waters column manager with an Acquity UPLC protein BEH C4 column (300 Å, 1.7 µm, 2.1 mm x 50 mm). Proteins were eluted at a column temperature of 80 ˚C with a flow rate of 0.3 mL/min. The following gradient was used: A = H_2O + 0.01% formic acid, B = MeCN + 0.01% formic acid. 5–95% B 0–6 min at 40 ˚C. Mass analysis was conducted with a Waters XEVO G2-XS Q-TOF analyzer. Proteins were ionized in positive ion mode, applying a cone voltage of 40 kV.

Phosphatase activity measurements

To measure the release of free inorganic phosphate after phosphatase treatment, the Phosphate Assay Kit (Sigma-Aldrich® Catalog # MAK308) was used as described in the manufacturer's protocol. Recombinant protein (100 U λ-phosphatase, 1 U FastAP, 5.4 nM rhPPA1, or 200 nM hPRUNE) was added to a reaction mixture (150 µL) containing 50 mM Tris-HCl (pH 8.0), 150 mM NaCl, 10 mM $MgCl_2$, and 10 µM phosphate-containing species. Additionally, 10 µM $Fe_2(SO_4)_3$ or $Al_2(SO_4)_3$ were added if indicated. Reactions were carried out at 37 °C for 1 h or at 4 °C for 3 h. Subsequently, the reaction mixtures (50 µL) were combined with Malachite Green reagent (100 µL) in a transparent 96-well plate, and after 30 min of equilibration at room temperature, the absorbance values (620 nm) were read out with a Tecan Infinite M Plex® plate reader.

Analytical HPLC characterization of peptides

For the characterization of isolated peptides, HPLC-UV traces were obtained on an Agilent Infinity 1260 LC system running on Agilent OpenLab CDS ChemStation Edition software.

Method A: Collected on an instrument fitted with a quaternary solvent delivery system (module no. G1311B), an autosampler (module no. G1329B), a column oven (module no. G1316C), a diode-array detector (module no. G4212B), and a fraction collector (module no. G1364C), using a YMC-Triart 5 µm C18 column (150 x 3.0 mm). Solvents: A = Milli-Q water + 0.1% TFA, B = acetonitrile + 0.1% TFA; Gradient: 5% B for 1 min, 5–40% B for 15 min, 40–90% B for 1 min, 90% B for 3 min; Flow rate: 1.0 mL/min; Temperature: 25 °C; Detection: 214 nm.

Enrichment workflow for endogenous identification of oligophosphorylation

The enrichment sample preparation workflow was with minor adaptations performed as described in Morgan *et al.*[1] (**Fig. 3e-f, Supplementary Fig. 16**). Briefly, HEK293T cells were cultured by seeding 1.2 x 10^6 into seven 15 cm culture dishes in DMEM complemented with 10% FBS, Penicillin-Streptomycin (100 U/mL), and Glutamine (2 mM). Cells were cultured for 4 d. To lyse, cells were washed twice with 5 mL 0.9% NaCl, and subsequently 2 mL lysis buffer (8 M urea, 75 mM NaCl, 50 mM Tris (pH 8.2), 1 mM NaF, 1 mM β-glycerolphosphate, 1 mM sodium orthovanadate, 10 mM sodium pyrophosphate, 1 mM PMSF, 1 cOmplete EDTA-free protease inhibitor tablet (Roche) per 10 mL) was added. Cells were scraped from the dishes and subjected to sonication at 4 °C. To reduce and alkylate proteins, DTT was added to a final concentration of 5 mM, and the sample was incubated at 37 °C for 1 h. Iodoacetamide was added to a concentration of 14 mM, and the sample was incubated for 30 min at room temperature in the dark. The remaining iodoacetamide was quenched with DTT. An in-solution tryptic digest was performed by diluting the lysate approx.1:5 with 25 mM Tris (pH 8.0), adding $CaCl_2$ to a concentration of 1 mM, adding sequencing-grade modified trypsin (1:50 protein-trypsin ratio) and incubating for 16 h at 37 °C and 500 rpm. After stopping the digestion and acidifying the sample by adding approx. 0.4% neat TFA (approx. pH 2), lysate desalting was performed using four SepPak tC18 3-cc 500 mg cartridges (Waters, WAT043425), loading approx. 20 mg per cartridge. The eluent was lyophilized and subsequently subjected to λ-phosphatase treatment. Therefore, 50 mg tryptic digest was dissolved in 10 mL of phosphatase reaction buffer (50 mM HEPES, 100 mM NaCl, 1 mM $MnCl_2$, 2 mM DTT, 0.01% Brij 35). The λ-phosphatase (50,000 units) was added and the solution was incubated at 37 °C and 300 rpm for 16 h. Approx. 0.4% neat TFA was added (approx. pH 2), and the sample was desalted as described in the previous step. A high-select Fe-NTA phosphopeptide enrichment Kit (Thermo Fisher Scientific) was used for subsequent sequential iron metal affinity chromatography (SIMAC).[1,2] For that, the tryptic digest (40 mg) treated with λ-phosphatase was dissolved in 800 µL of SIMAC loading buffer (0.1% TFA, 50% MeCN). Four columns were washed twice with 200 µL of SIMAC loading buffer and then closed with a plug supplied with the kit and loaded with 200 µL digest solution (10 mg per column). The loaded columns were incubated for 30 min at room temperature while resuspending the Fe-NTA by gently tapping

the columns for 10 s every 10 min in the peptide solution. Columns were washed several times, including two washes with SIMAC buffer A (1% TFA, 20% MeCN, to elute monophosphorylated peptides). The final elution was performed twice with 100 µL SIMAC buffer B (0.5% NH_4OH in water). The combined peptide eluent (800 µL) was directly frozen and lyophilized overnight. For fractionation, the material was dissolved in 100 µL HPLC buffer A (20% MeCN, 1% FA, pH 9.0 set with NH_4OH). The fractionation was performed on an Agilent 1260 Infinity HPLC, equipped with an IonPac AS24 2 mm analytical ultra-hydrophilic SAX column (Thermo Fisher Scientific). HPLC buffer A (see above). HPLC buffer B (1% MeCN, 1% NH_4OH, pH 2.8 set with FA). Flow rate: 0.2 mL/min. Gradient: 0–5 min, 100% A, 5–50 min, gradient increase to 100% B, 50-60 min, 100%B. The fractions were collected for 5 min each, for a total of twelve 1 mL fractions. The fractions were transferred to suitable MS vials, dried, and stored at -20 °C until LC-MS/MS. They were resuspended in a 50 mM medronic acid solution in Milli-Q water/MeCN = 97:3 (+0.1% FA) and measured using stepped-HCD (20%–23%–26%).

Label-free quantification analysis of oligophosphorylated peptides from enriched HEK293T lysate

The obtained raw data was analyzed using FragPipe (v22) by adapting the built-in LFQ–phospho workflow and the Protein IDs (obtained from a FragPipe analysis using the same MSFragger and validation settings) of the data as search space (**Supplementary Fig. 16**). The following MSFragger settings were applied: Precursor mass tolerance: -20 to +20 Da; Fragment mass tolerance: +/- 20 ppm; Mass calibration & parameter optimization enabled; Isotope error: 0/1/2/3; Enzyme: Trypsin (cuts after K & R, no cut before P) with 2 missed cleavages, Peptide length: 7-50 AA; Peptide mass range: 500-5000 Da; Variable modifications included all identified modifications of the dataset (see main manuscript Methods: “Identification of oligophosphorylated peptides from enriched HEK293T lysate”: Oxidation (M, +15.9949 Da, up to 3x), Phosphorylation (STY, +79.96633, up to 3x), Pyrophosphorylation (ST, +159.93266, up to 3x), Pyrophosphorylation_Fe (ST, +212.8435, up to 3x), Triphosphorylation_Fe (ST, +292.8099, up to 3x), Triphosphorylation_Al (ST, +26.8571, up to 3x), Tetraphosphorylation_Fe (ST, +372.7762, up to 3x); Carbamidomethylation of C was set as fixed modification (+57.02146 Da). Validation was performed using Percolator (using default settings) and ProteinProphet. A protein-level FDR of 1% was applied. PTM-Prophet was enabled to load the modifications from MSFragger settings. The MS1 quantification default settings were used.

Enrichment workflows for endogenous absolute quantification of oligophosphorylation

The experiment covers the wt, monophosphorylated, pyrophosphorylated, and triphosphorylated state of NME1 (**Extended Data Fig. 4, Supplementary Fig. 17-20**). The enrichment sample preparation workflow was adapted from Morgan *et. al*[1], depending on the phosphorylation state, several steps were excluded, and the off-line fractionation before the LC-MS/MS measurement was performed on an Atlantis Premier BEH C18 AX column (Waters). The experiment was performed in biological triplicate for every phosphorylation state. Briefly, HEK293T cells were cultured by seeding 1.2 x 10^6 into seven (for each phosphorylation, pyrophosphorylation, and triphosphorylation batch) or one (for each wt batch) 15 cm culture dish in DMEM complemented with 10% FBS, Penicillin-Streptomycin (100 U/mL), and Glutamine (2 mM). Cell harvest, lysis, and digestion were equally performed for all samples as described above in the "Enrichment workflow for endogenous identification of oligophosphorylation" section. Protein amount determination was performed *via* BCA. Before digestion, 2 pmol of a respective SCAR standard peptide was spiked into the "phosphorylation", "pyrophosphorylation", and "triphosphorylation" batches, and 1 pmol of SCAR standard peptide was spiked into 40 µg lysate of the wt batches.

The following further sample preparation steps were performed for the "wt" batches: Desalting using oneSepPak tC18 3-cc 500 mg cartridge, and the offline-fractionation.

The following further sample preparation steps were performed for the "phosphorylation" batches: Desalting using oneSepPak tC18 3-cc 500 mg cartridges, SIMAC enrichment, and the offline-fractionation.The SIMAC enrichment was adapted by collecting the elution with SIMAC buffer A (1% TFA, 20% ACN) as well as the elution with SIMAC buffer B (0.5% NH_4OH in water).

The "pyrophosphorylation" and "triphosphorylation" batches were treated as described above in "Enrichment workflow for endogenous identification of oligophosphorylation".

For fractionation, each sample was dissolved in 100 µL of 94.4% HPLC buffer A (MQ, 0.1% formic acid) and 5.6% HPLC buffer B (90% ACN, 5 mM ammonium formate, set to pH 3 with formic acid. The fractionation was performed on an Agilent 1260 Infinity HPLC, equipped with an Atlantis Premier BEH C18 AX column (Waters). HPLC buffer A (see above). Flow rate: 0.2 mL/min. Gradient: 0–56 min, gradient increase from 5.6% B to 21.1% B; 56-56.5 min, gradient increase to 55.6% B; 56.5–60 min, 55.6%B. The column oven temperature was set to 25 °C. The fractions were collected for 5 min each, for a total of twelve 1 mL fractions.

The fractions were transferred to suitable MS vials, dried, and stored at -20 °C until LC-MS/MS. They were resuspended in 50 mM medronic acid buffer and measured using a targeted PRM method with stepped-HCD (20%–23%–26%) fragmentation on an Orbitrap Lumos instrument. The inclusion list was specific for the respective phosphorylation state of interest. The fractions containing the peptide of interest were combined for the final measurement using the same method.

Absolute quantification analysis of oligophosphorylated peptides from enriched HEK293T lysate

For peptide identification, the obtained raw data was analyzed using FragPipe (v22) with NME1 and SCAR_NME1 as search space. The following MSFragger settings were applied: Precursor mass tolerance: -20 to +20 Da; Fragment mass tolerance: +/- 20 ppm; Mass calibration & parameter optimization enabled; Isotope error: 0/1/2/3; Enzyme: Trypsin (cuts after K & R, no cut before P) with 2 missed cleavages, Peptide length: 7-50 AA; Peptide mass range: 500-5000 Da; Variable modifications included all modifications of the respective targeted (phospho)species up to 3x and Oxidation (M, +15.9949 Da, up to 3x); Carbamidomethylation of C was set as fixed modification (+57.02146 Da). Validation was performed using Percolator (using default settings) and ProteinProphet. A protein-level FDR of 1% was applied. PTM-Prophet was enabled to load the modifications from MSFragger settings. The peak area ratio (endogenous *versus* SCAR) of the identified species was determined using Skyline 24.1.0.[3] The transitions determined in validation experiments (**Extended Data Fig. 4, Supplementary Fig. 17-20**) were used (transitions showing interferences were excluded), and the resulting total peak area for each peptide was extracted. The peak area ratio was used to calculate the endogenous peptide concentrations normalized to the protein amount of the HEK lysate in fmol/µg.

Label-free quantification analysis of pT94- (and pS94-)NME1

The obtained raw data was analyzed using FragPipe (v22) by adapting the built-in LFQ–phospho workflow[4] and NME1_T94S plus contaminants and/or the human proteome as search space. The following MSFragger settings were applied: Precursor mass tolerance: -20 to +20 Da; Fragment mass tolerance: +/- 20 ppm; Mass calibration & parameter optimization enabled; Isotope error: 0/1/2/3; Enzyme: Trypsin (cuts after K & R) with 2 missed cleavages, Peptide length: 7-50 AA; Peptide mass range: 500-5000 Da; Variable modifications: Oxidation (M, +15.9949 Da, up to 3x), Acetylation (N-term, +42.0106 Da), Phosphorylation (STY, +79.96633, up to 3x); Carbamidomethylation of C was set as fixed modification (+57.02146 Da). Validation was performed using Percolator (using default settings) and ProteinProphet (Philosopher v5.1.1). A protein-level FDR of 1% was applied. PTM-Prophet was enabled to load the modifications from MSFragger settings. The MS1 quantification default settings were used.

Cryo-EM

Cryo-EM sample preparation and data acquisition.

For pS94-NME1 and ppS94-NME1, purified protein was vitrified on Quantifoil 1.2/1.3 Cu 300 mesh grids at 0.9 mg/ml using a Vitrobot Mark IV set to a blot force of 0, blotting time of 3.0 For oligo-pS94-NME1, ppS94-NME1 was incubated at 1 mM concentration with ATP for 3 h at 37 ˚C before vitrification as above. Micrographs for all three data sets were acquired using a FEI Titan Krios G3i microscope (Thermo Fisher Scientific) operated at 300 kV equipped with a Bioquantum K3 direct electron detector and energy filter (Gatan) running in CDS counting mode at a slit width of 20 eV and a nominal magnification of 105,000×, giving a calibrated physical pixel size of 0.83 Å/px on the specimen level. EPU 2.12 was utilized for automated data acquisition with AFIS enabled. For pS94-NME1, movies were recorded for 2.0 s, accumulating a total electron dose of 47.7 $e^{-}/Å^{2}$ fractionated into 50 frames. Nominal defocus values were between -1.1 and -2.6 µm. For ppS94-NME1, movies were recorded for 2.0 s, resulting in a total electron dose of 44.6 $e^{-}/Å^{2}$ fractionated into 50 frames with nominal defocus values between -1.2 and -2.4. For oligo-pS94-NME1, movies were recorded for 2.0 s, accumulating a total electron dose of 44.1 $e^{-}/Å^{2}$ distributed over 50 frames. Here, nominal defocus values were between -1.4 and -2.4.

Data processing of pS94-NME1

All data processing steps were carried out using CryoSPARC and are outlined in **Extended Data Fig. 5f**.[5] 3,555 movies were aligned using Patch Motion correction, and CTF was determined using patch CTF estimation. After sorting out bad images, 3190 micrographs were selected for further processing. 90,493 particles were selected from 50 micrographs using the blob picker and extracted for initial 2D classification and generation of autopicking templates. Subsequent template-based autopicking using 14 selected 2D classes as templates and particle curation identified 3,721,838 particles. Of these, 3,559,391 were extracted (2x binning) for 2D classification. After three rounds of 2D classification (70 online-EM interactions), an initial 3D hetero refinement (3 classes), and duplicate removal, 1,038,254 particles were re-extracted without binning and subjected to non-uniform refinement using D3 symmetry and a molecular map of PDB 5UI4 (lowpass filtered to 12 Å) as an initial model.[6] After iterative rounds of global and local CTF refinement and local motion correction, 1,038,113 particles were sorted in two successive rounds of 3D hetero refinement (4 classes each), resulting in a final class of 533,016 particles.[7] These were then further processed using non-uniform refinement, followed by reference-based motion correction, yielding 532,096 particles. A final non-uniform refinement resulted in a resolution of 2.8 Å according to the gold-standard Fourier Shell Correlation (FSC) criterion (**Extended Data Fig. 5c,e**). DeepEMhancer was applied for map sharpening.[8]

Data processing of ppS94-NME1.

All data processing steps were performed using CryoSPARC and are outlined in Figure **Extended Data Fig. 6f**.[5] 6,346 movies were aligned using patch motion correction, and patch CTF estimation was used to determine the CTF. After rejecting bad images, 4639 micrographs were selected for further processing and subjected to particle picking using the template picker, which identified 1,629,454 particles. Particles were extracted with 2x binning, resulting in 1,546,555 particles that were sorted using two successive 2D classifications (70 online-EM iterations). 2D classes were selected and 782,199 corresponding particles were extracted (no binning), resulting in 780,780 particles that were subjected to non-uniform refinement using 3D symmetry and the pS94-NME1 map as the initial model.[6] After iterative rounds of global and local CTF refinement and local motion correction, 780,686 particles were sorted using heterogeneous refinement.[7] One class with 486,698 particles was selected and further refined using non-uniform refinement, which was followed by a local CTF refinement and reference-based motion correction. The resulting 486,617 particles were refined with non-uniform refinement, resulting in a resolution of 3.31 Å according to the gold-standard Fourier Shell Correlation (FSC) criterion (**Extended Data Fig. 6c,e**). DeepEMhancer was applied for map sharpening.[8]

Data processing of oligo-pS94-NME1

All data processing steps were carried out using CryoSPARC and are outlined in **Extended Data Fig. 7f, i**.[5] 4,952 movies were aligned using Patch Motion correction, and the CTF was determined using patch CTF estimation. After rejecting bad images, 4856 micrographs were selected for further processing. Particles were picked using the template picker, identifying 2,650,681 particles, which were extracted using 2x binning, retaining 2,121,197 particles. Sorting was performed using three consecutive rounds of 2D classification (70 online-EM iterations), resulting in a total of 508,065 total particles. After extraction (no binning) 507,420 particles were retained, subjected to non-uniform refinement with D3 symmetry using the ppS94-NME1 map as an initial model, and further sorted using heterogeneous refinement.[6] The class containing 201,312 particles was selected and refined using non-uniform refinement, resulting in a resolution of 3.79 Å according to the gold-standard Fourier Shell Correlation (FSC) criterion (**Extended Data Fig. 7c,e**). DeepEMhancer[8] was used for map sharpening. To investigate the heterogeneity of S94 phosphorylation, the particles were symmetry expanded, resulting in 1,207,872 particles that were subjected to four consecutive 3D classifications using a mask focused on one NME1 monomer and forced hard classification. Three classes representing 283,953 particles were each subjected to local refinement using a focus mask on one NME1 monomer, resulting in three maps of 4.53 Å (105,753 particles), 4.40 Å (89,700 particles), and 4.60 Å (88,500 particles) according to the gold-standard Fourier Shell Correlation (FSC) criterion (**Extended Data Fig. 7i**).

Atomic modeling of pS94-NME1

The atomic model of human NME1 (PDB 5UI4) was used as a starting model, and the attached imidazole fluorosulfate group and water molecules were removed. The model was rigid-body fitted into the sharpened density map using UCSF ChimeraX,[9] manually adjusted in Coot,[10] where Thr94 was mutated to phosphoserine and ISOLDE,[11] and then refined using real-space refinement in Phenix (**Extended Data Fig. 5g,h**).[12] Cryo-EM data processing and model refinement statistics are summarized in **Supplementary Table 1**.

Atomic modeling of ppS94-NME1 and pppS94-NME1

The atomic model of pS94-NME1 (see above) was used as a starting point. The model was first rigid-body fitted into the obtained sharpened density maps for ppS94-NME1 and oligo-pS94-NME1, respectively, using UCSF ChimeraX[9] and then manually adjusted in Coot. Geometry restraints for ppS and pppS were generated using phenix.elbow,[13] and the resulting cif files were manually changed from a ligand to an amino acid. The changed amino acids were incorporated in the models, manually adjusted in Coot, and then refined using real-space refinement in Phenix (**Extended Data Fig. 6g,h** and **Extended Data Fig. 7g,h**).[12] Cryo-EM data processing and model refinement statistics are summarized in **Supplementary Table 1**.

Chemical Synthesis and Characterization

Biotin-PEG$_6$-triazole-NPE-(1*H*-imidazolide-1-yl)phosphonate

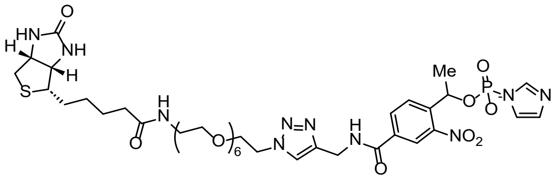

Biotin-PEG$_6$-triazole-NPE-p-imidazolide was synthesized according to previously published protocols.[14] Spectral data matches previously reported values.

Lithium 1-(2-nitrophenyl)ethyl-1*H*-imidazol-1-ylphosphonate

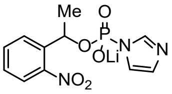

NPE-p-imidazolide was synthesized according to previously published protocols.[14] Spectral data matches previously reported values.

NPE-trioligophospho-imidazolide

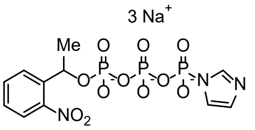

NPE-p$_3$-imidazolide was synthesized according to previously published protocols.[15] Spectral data matches previously reported values.

Pep-endo

H_2N-Val-Met-Leu-Gly-Glu-Thr-Asn-Pro-Ala-Asp-Ser-Lys-Pro-Gly-Thr-Ile-Arg-COOH

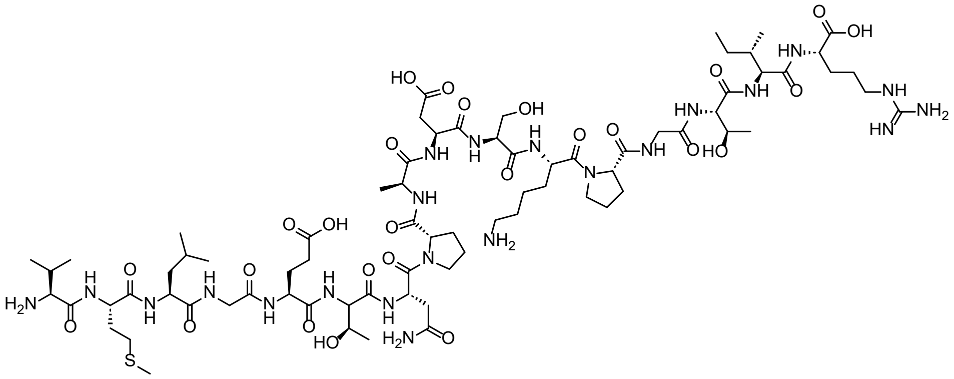

Pep-endo was synthesized by solid-phase peptide synthesis (SPPS) according to literature-known protocols.[15]

HRMS (ESI): m/z calc'd. for $C_{75}H_{130}N_{22}O_{26}S^{2+}$ $[M+2H]^{2+}$ 893.4618, found 893.4608.
T_R (Method A) = 10.048 min.

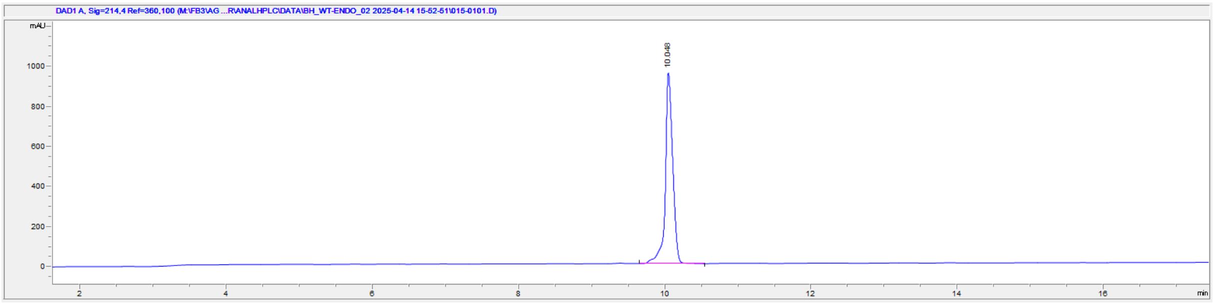

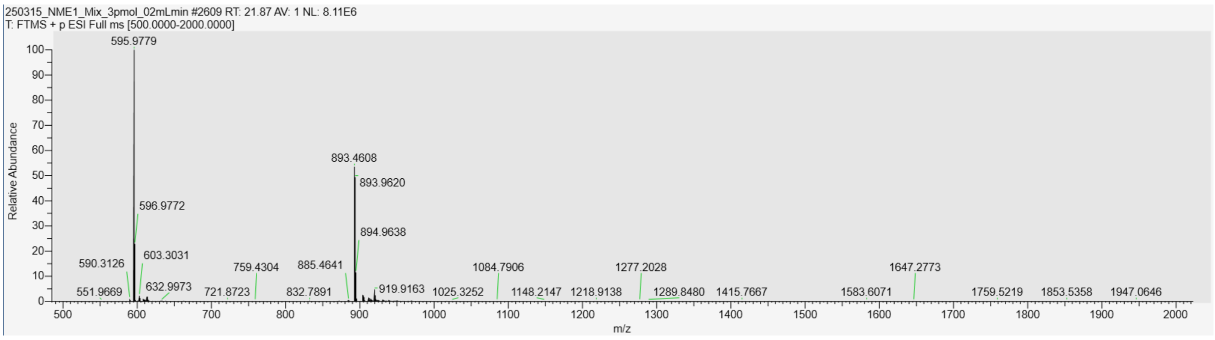

p-Pep-endo

H_2N-Val-Met-Leu-Gly-Glu-[pThr]-Asn-Pro-Ala-Asp-Ser-Lys-Pro-Gly-Thr-Ile-Arg-COOH

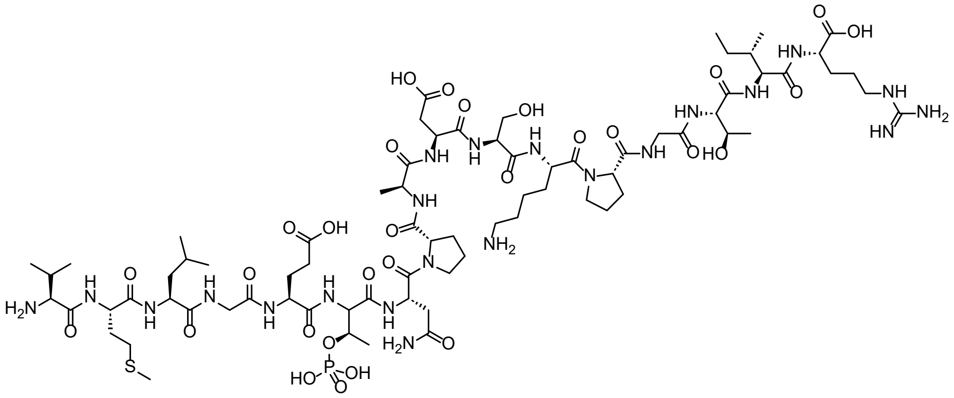

p-Pep-endo was synthesized by SPPS according to previously published protocols.[15] Spectral data matches previously reported values.

p$_2$-Pep-endo

H_2N-Val-Met-Leu-Gly-Glu-[ppThr]-Asn-Pro-Ala-Asp-Ser-Lys-Pro-Gly-Thr-Ile-Arg-COOH

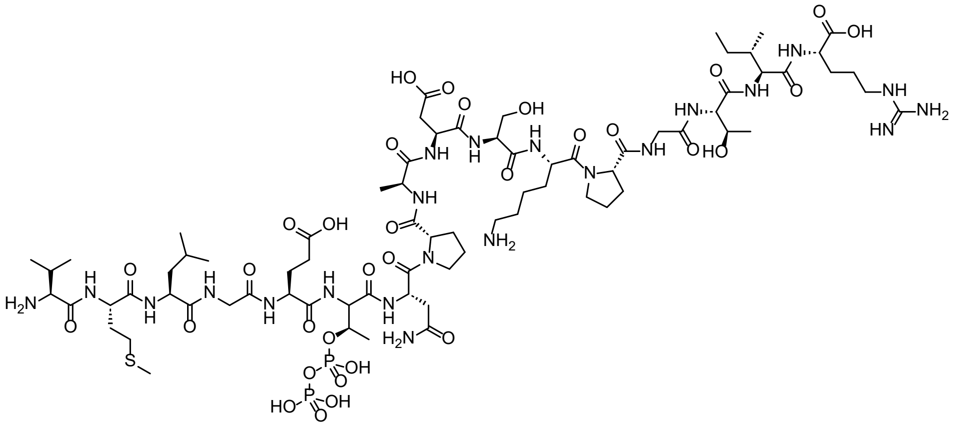

p$_2$-Pep-endo was synthesized by subjecting p-Pep-endo to the reaction with NPE-p-imidazolide followed by UV-deprotection according to previously published protocols.[15] Spectral data matches previously reported values.

p_3-Pep-endo

H_2N-Val-Met-Leu-Gly-Glu-[pppThr]-Asn-Pro-Ala-Asp-Ser-Lys-Pro-Gly-Thr-Ile-Arg-COOH

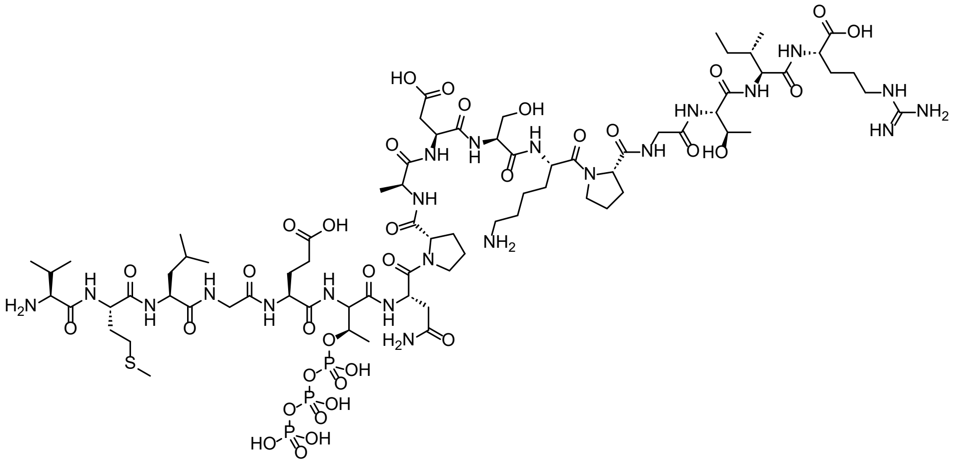

p_3-Pep-endo was synthesized by subjecting p_2-Pep-endo to the reaction with NPE-p-imidazolide followed by UV-deprotection according to previously published protocols.[15] Spectral data matches previously reported values.

p_4-Pep-endo

H_2N-Val-Met-Leu-Gly-Glu-[ppppThr]-Asn-Pro-Ala-Asp-Ser-Lys-Pro-Gly-Thr-Ile-Arg-COOH

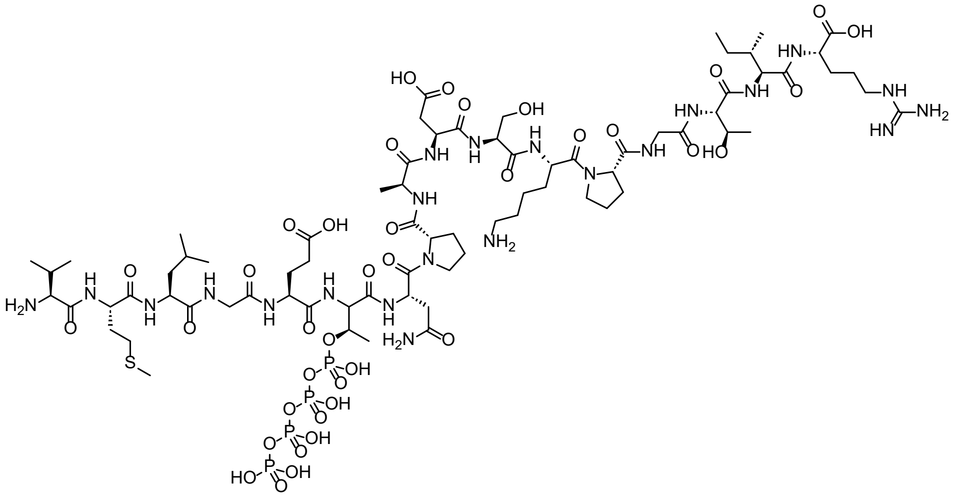

p_4-Pep-endo was synthesized by subjecting p-Pep-endo to the reaction with NPE-p_3-imidazolide followed by UV-deprotection according to previously published protocols.[15] Spectral data matches previously reported values.

Pep-SCAR

H_2N-Val-Met-Leu-Gly-Glu-Thr-Asn-Pro-Ala-Asp-Ser-Lys-Pro-Ala-Thr-Ile-Arg-COOH

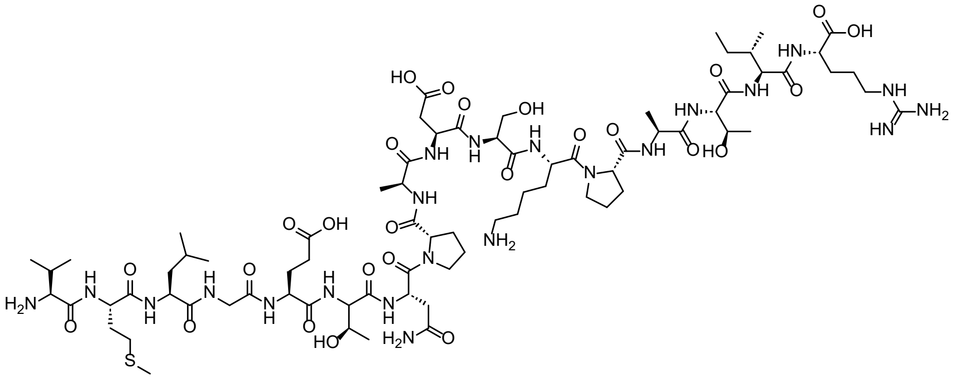

Pep-SCAR was synthesized in the same manner as Pep-endo by SPPS according to literature-known protocols.[15]

HRMS (ESI): m/z calc'd. for $C_{76}H_{132}N_{22}O_{26}S^{2+}$ $[M+2H]^{2+}$ 900.4697, found 900.4685.

T_R (Method A) = 10.114 min.

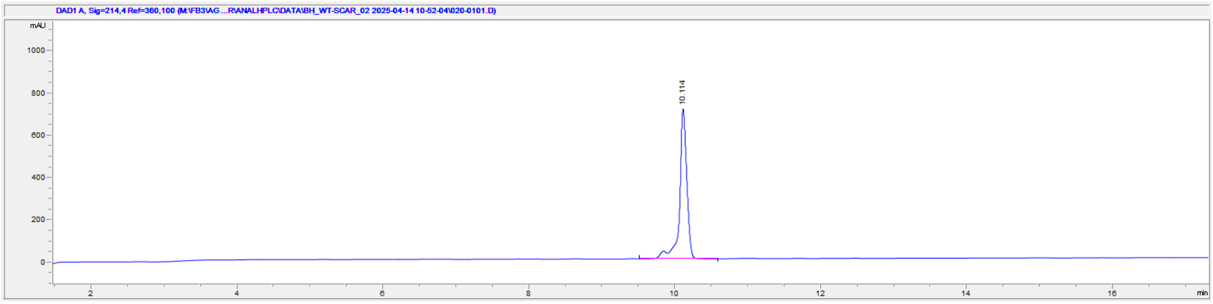

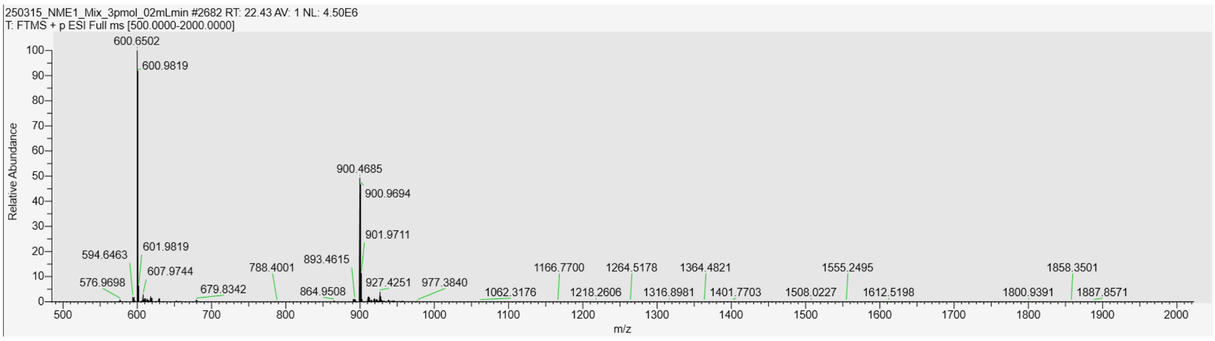

p-Pep-SCAR

H_2N-Val-Met-Leu-Gly-Glu-[pThr]-Asn-Pro-Ala-Asp-Ser-Lys-Pro-Ala-Thr-Ile-Arg-COOH

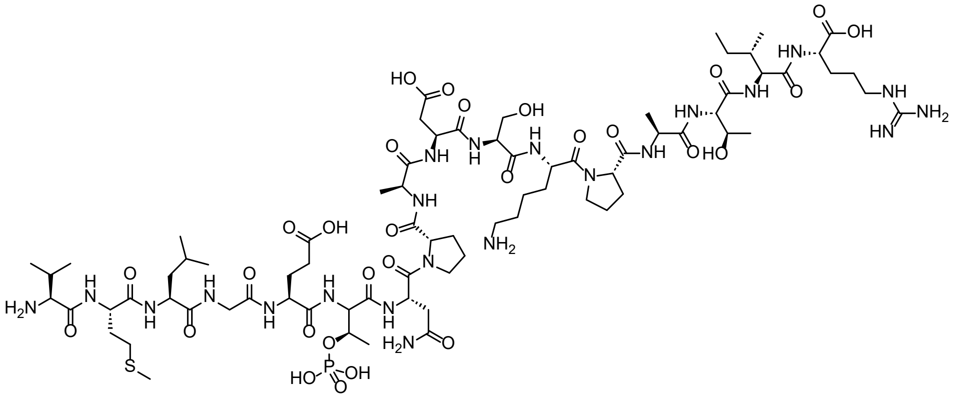

p-Pep-SCAR was synthesized in the same manner as p-Pep-endo by SPPS according to previously published protocols.[15]

HRMS (ESI): m/z calc'd. for $C_{76}H_{133}N_{22}O_{29}PS^{2+}$ $[M+2H]^{2+}$ 940.4528, found 940.4530.

T_R (Method A) = 9.802 min.

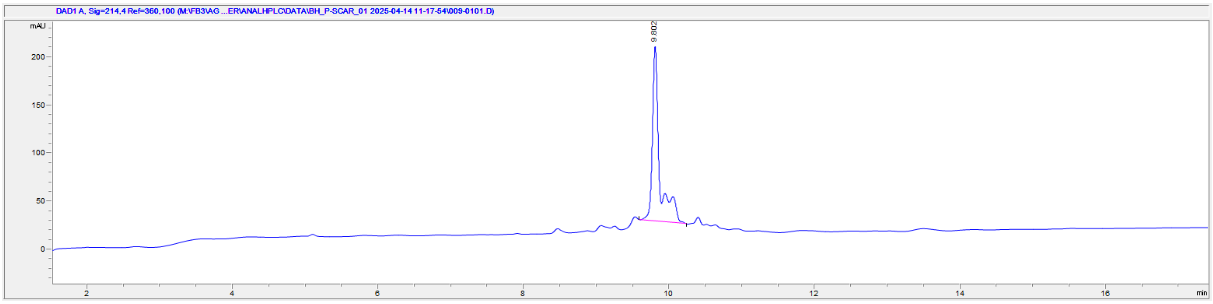

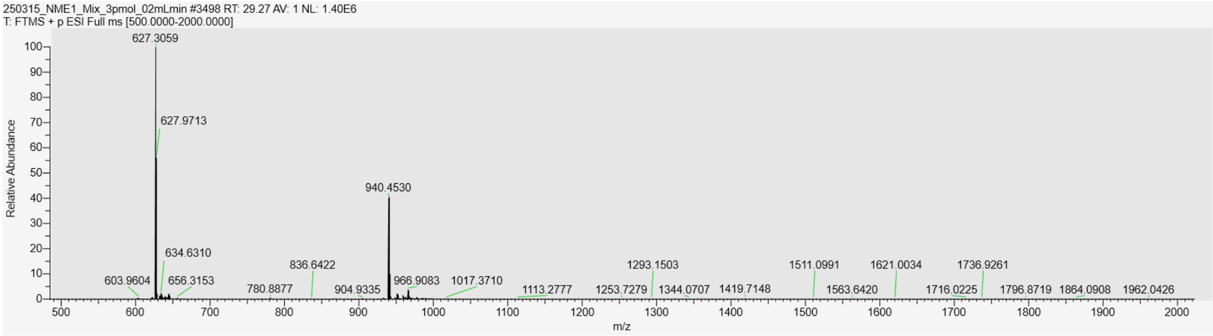

p_2-Pep-SCAR

H_2N-Val-Met-Leu-Gly-Glu-[ppThr]-Asn-Pro-Ala-Asp-Ser-Lys-Pro-Ala-Thr-Ile-Arg-COOH

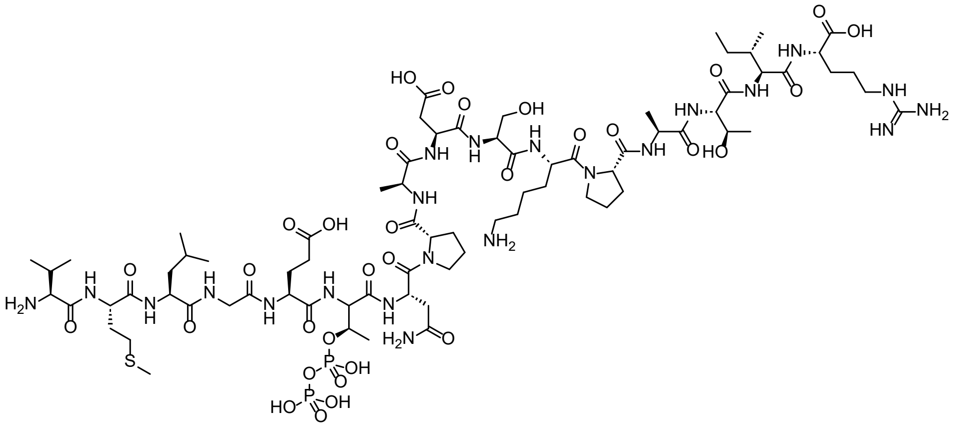

p_2-Pep-SCAR was synthesized in the same manner as p_2-Pep-endo by subjecting p-Pep-SCAR to the reaction with NPE-p-imidazolide followed by UV-deprotection according to previously published protocols.[15]

HRMS (ESI): m/z calc'd. for $C_{76}H_{134}N_{22}O_{32}P_2S^{2+}$ $[M+2H]^{2+}$ 980.4360, found 980.4371.
T_R (Method A) = 9.653 min.

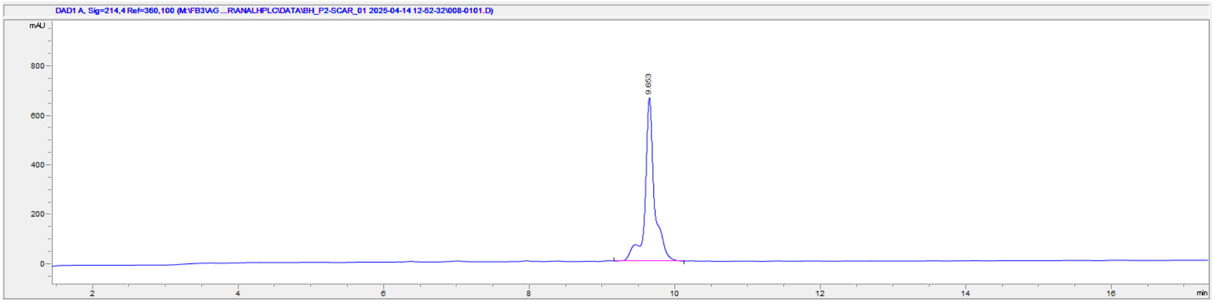

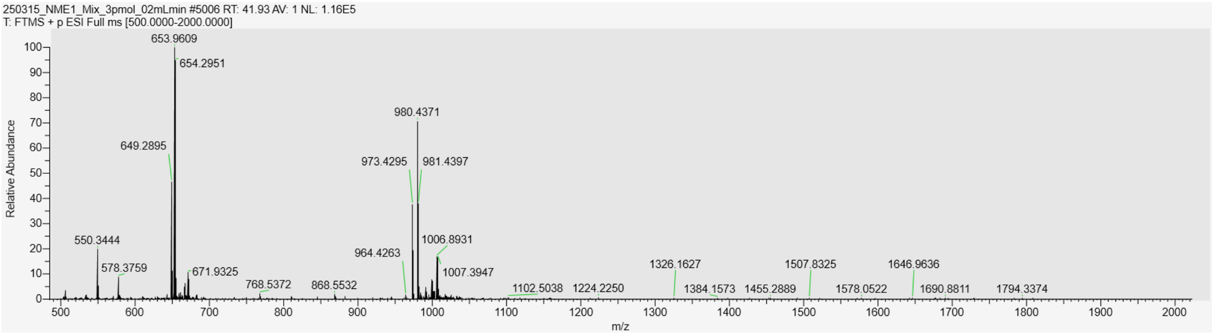

p_3-Pep-SCAR

H_2N-Val-Met-Leu-Gly-Glu-[pppThr]-Asn-Pro-Ala-Asp-Ser-Lys-Pro-Ala-Thr-Ile-Arg-COOH

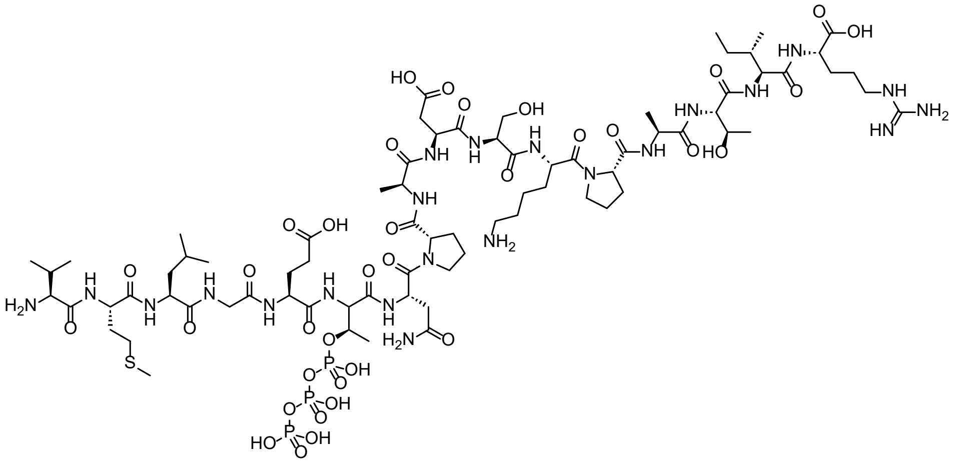

p_3-Pep-SCAR was synthesized in the same manner as p_3-Pep-endo by subjecting p_2-Pep-SCAR to the reaction with NPE-p-imidazolide followed by UV-deprotection according to previously published protocols.[15]

HRMS (ESI): m/z calc'd. for $C_{76}H_{132}AlN_{22}O_{35}P_3S^{2+}$ $[M-H+Al]^{2+}$ 1032.3982, found 1032.3993. T_R (Method A) = 9.477 min (Al^{3+} adduct), 9.672 min (Fe^{3+} adduct).

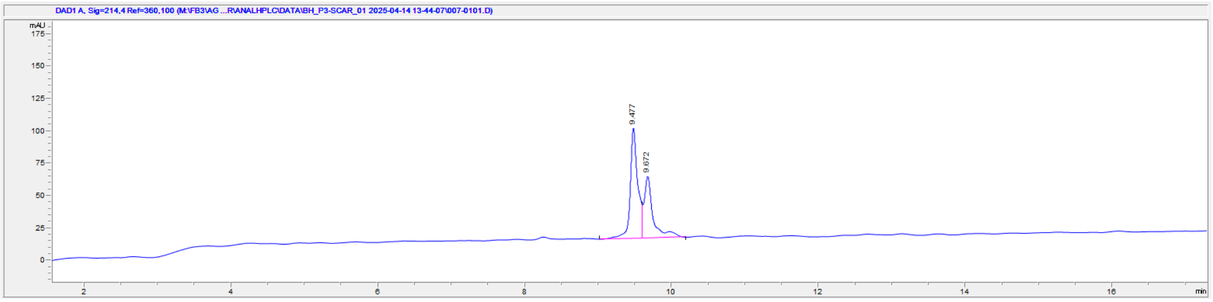

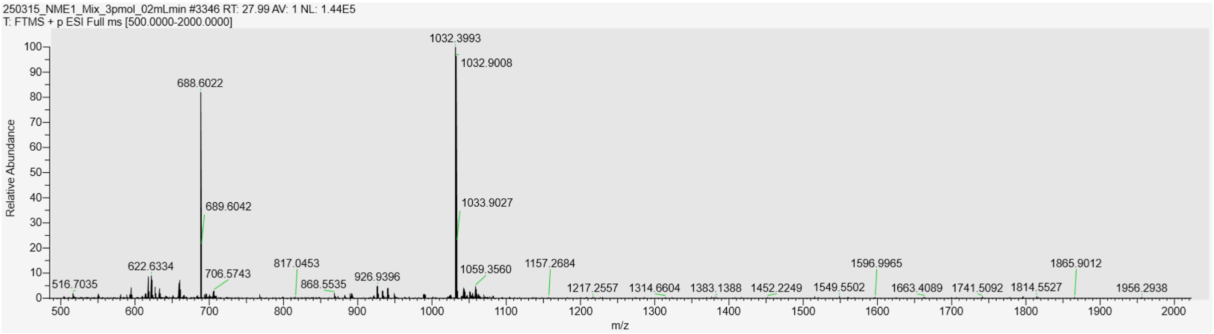

p_4-Pep-SCAR

H_2N-Val-Met-Leu-Gly-Glu-[ppppThr]-Asn-Pro-Ala-Asp-Ser-Lys-Pro-Ala-Thr-Ile-Arg-COOH

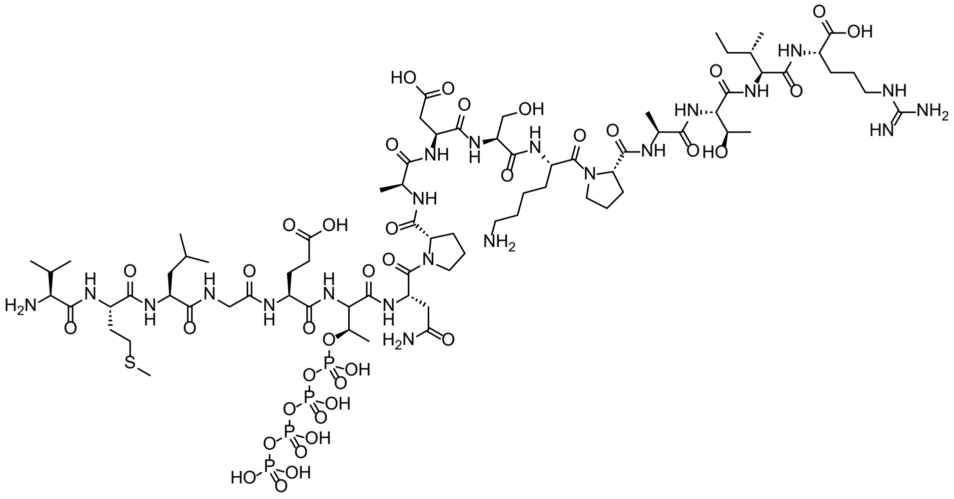

p_4-Pep-SCAR was synthesized in the same manner as p_4-Pep-endo by subjecting p-Pep-SCAR to the reaction with NPE-p_3-imidazolide followed by UV-deprotection according to previously published protocols.[15]

HRMS (ESI): m/z calc'd. for $C_{76}H_{133}AlN_{22}O_{38}P_4S^{2+}$ $[M-H+Al]^{2+}$ 1072.3814, found 1072.3826. T_R (Method A) = 9.467 min.

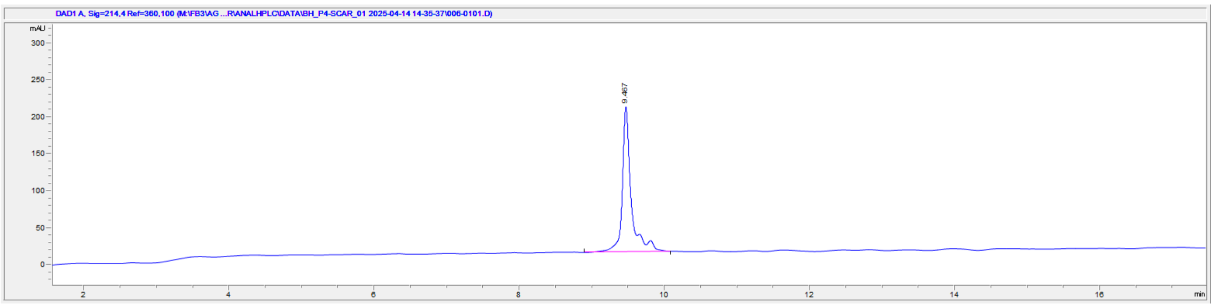

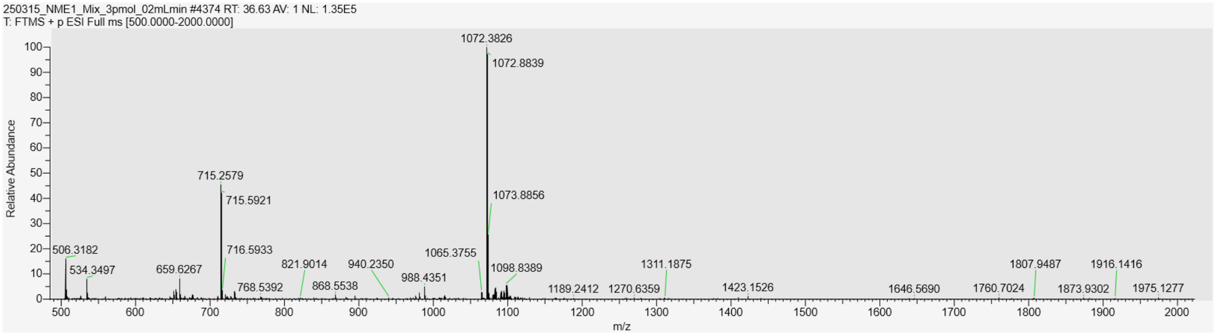

13

Protein Q-TOF-MS Spectra

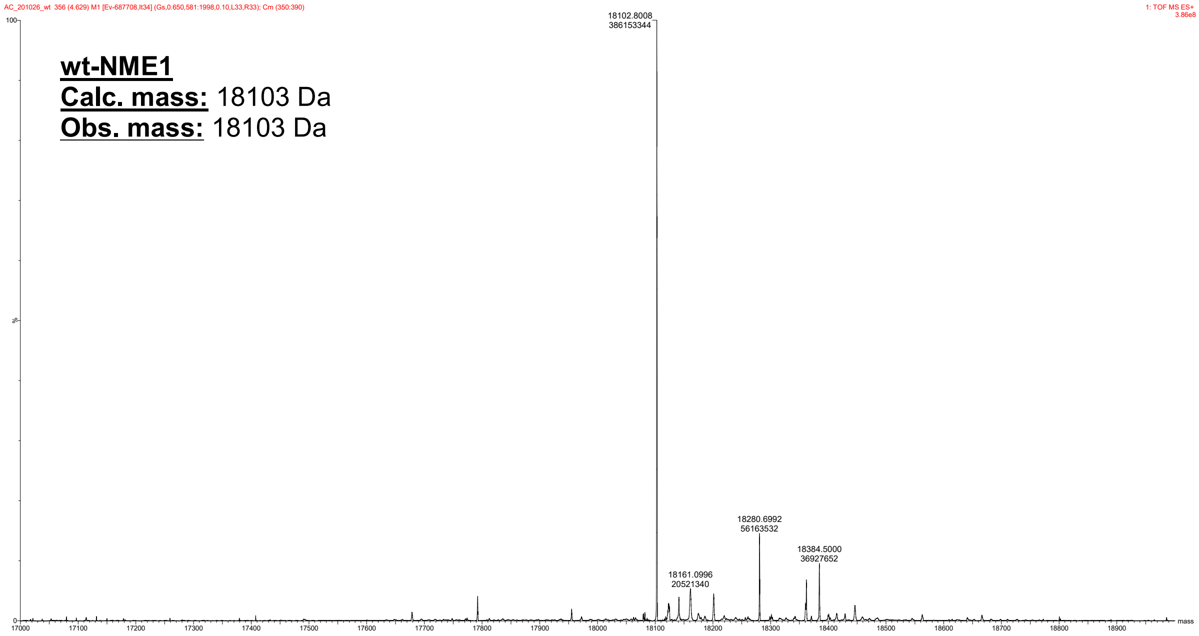

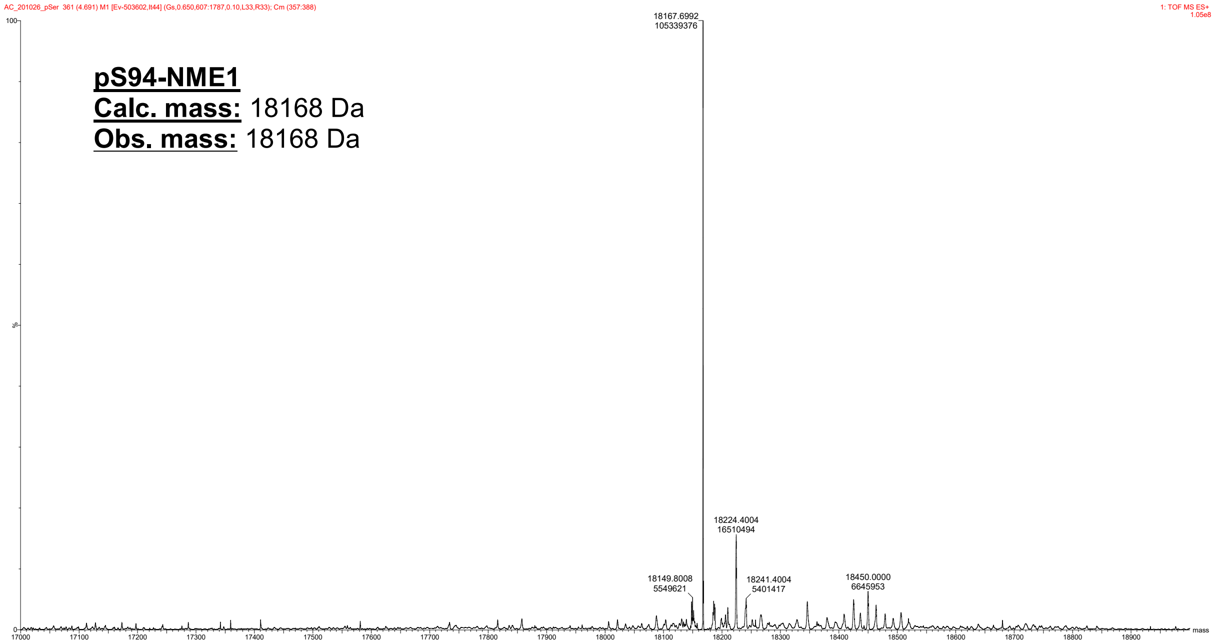

Sequence of pS94-NME1:

MHHHHHHMANCERTFIAIKPDGVQRGLVGEIIKRFEQKGFRLVGLKFMQASEDLLKEHYVDL
KDRPFFAGLVKYMHSGPVVAMVWEGLNVVKTGRVMLGE[pS]NPADSKPGTIRGDFCIQVG
RNIIHGSDSVESAEKEIGLWFHPEELVDYTSCAQNWIYE

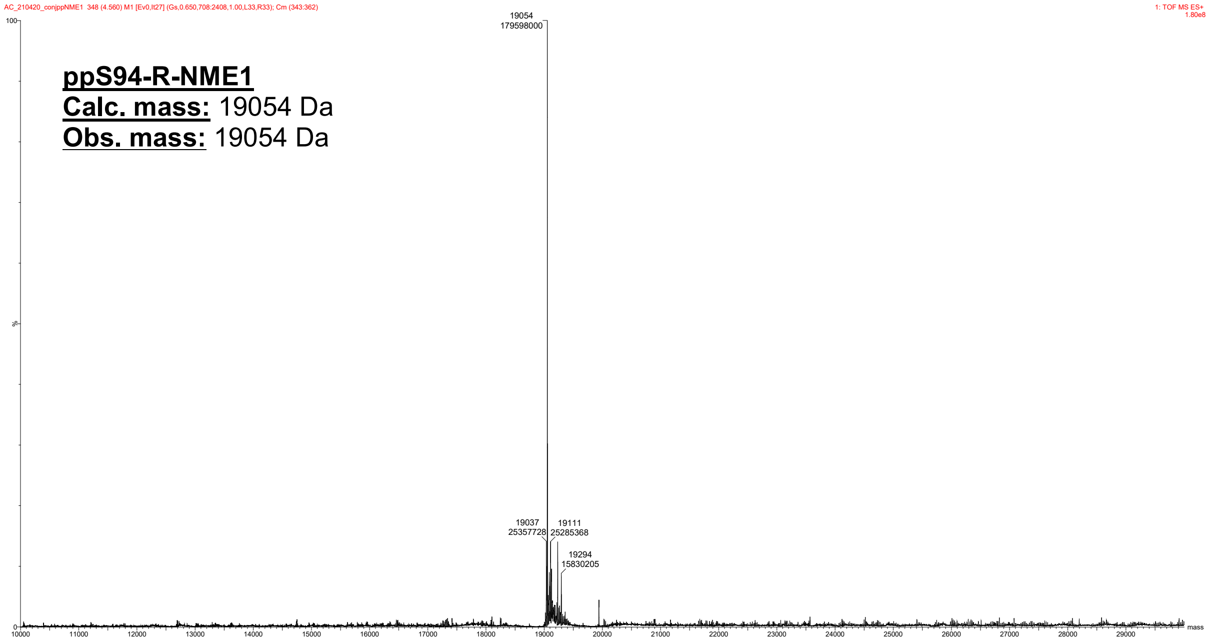
AC_210420_conjppNME1 348 (4.560) M1 [Ev0,It27] (Gs,0.650,708:2408,1.00,L33,R33); Cm (343:362)
1: TOF MS ES+
1.80e8
ppS94-R-NME1
Calc. mass: 19054 Da
Obs. mass: 19054 Da
19054
179598000
19037
25357728
19111
25285368
19294
15830205
mass
%

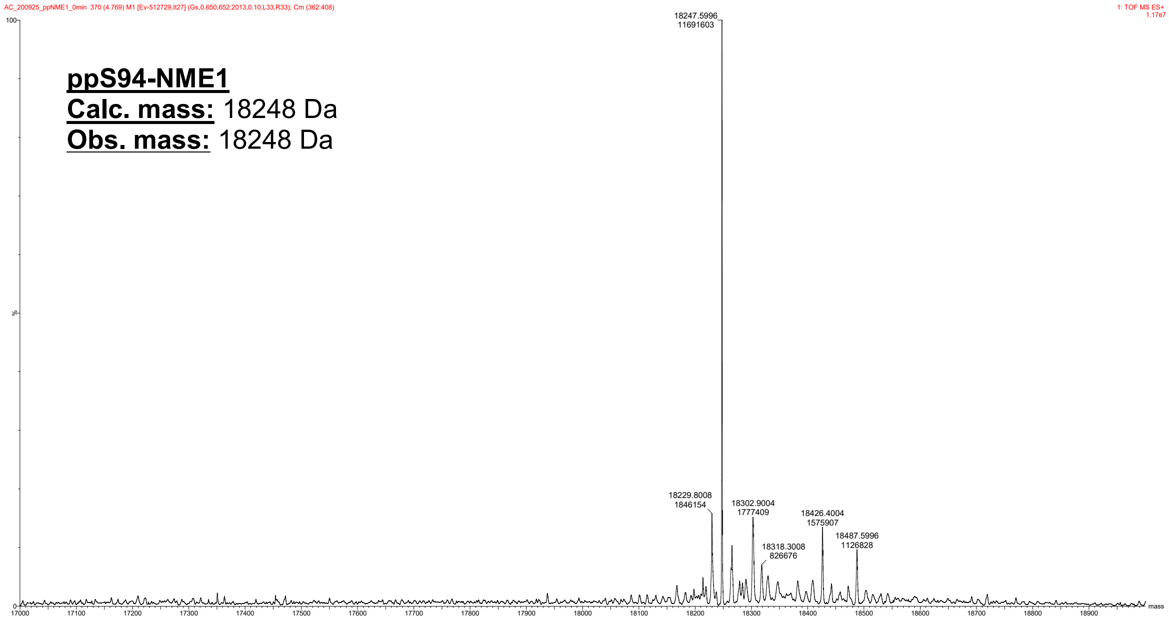
AC_200925_ppNME1_0min 370 (4.769) M1 [Ev-512729,It27] (Gs,0.650,652:2013,0.10,L33,R33); Cm (362:408)
1: TOF MS ES+
1.17e7
ppS94-NME1
Calc. mass: 18248 Da
Obs. mass: 18248 Da
18247.5996
11691603
18229.8008
1846154
18302.9004
1777409
18318.3008
826676
18426.4004
1575907
18487.5996
1126828
%
mass

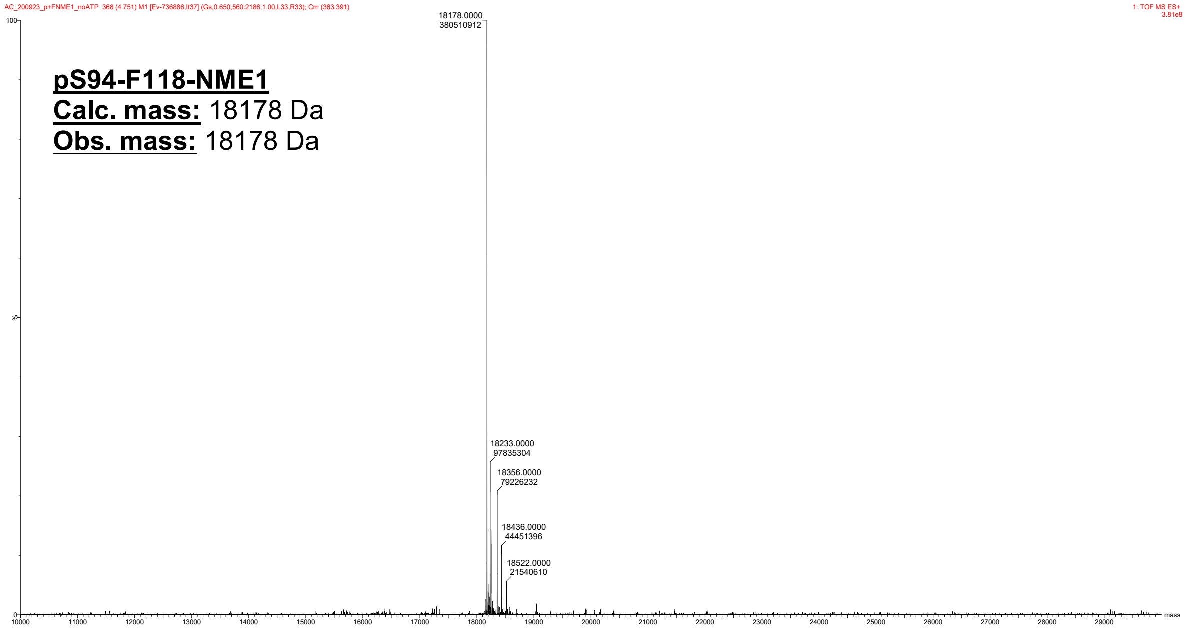

AC_200923_p+FNME1_noATP 368 (4.751) M1 [Ev-736886,It37] (Gs,0.650,560:2186,1.00,L33,R33); Cm (363:391)
1: TOF MS ES+
3.81e8
pS94-F118-NME1
Calc. mass: 18178 Da
Obs. mass: 18178 Da
18178.0000
380510912
18233.0000
97835304
18356.0000
79226232
18436.0000
44451396
18522.0000
21540610
mass

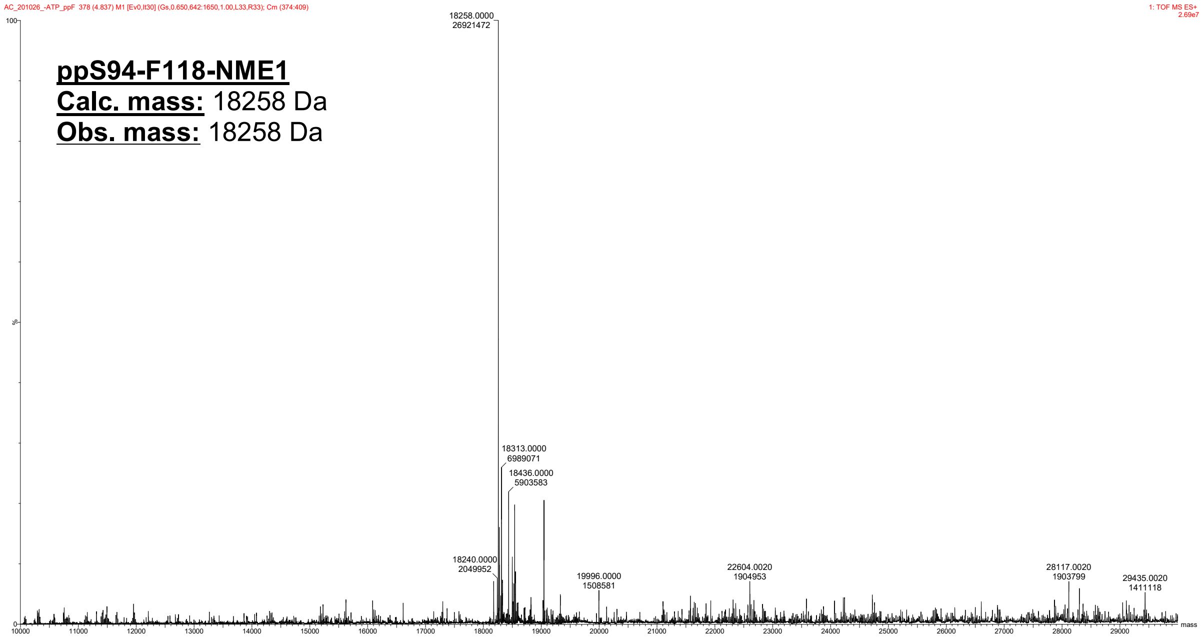

AC_201026_-ATP_ppF 378 (4.837) M1 [Ev0,It30] (Gs,0.650,642:1650,1.00,L33,R33); Cm (374:409)
1: TOF MS ES+
2.69e7
ppS94-F118-NME1
Calc. mass: 18258 Da
Obs. mass: 18258 Da
18258.0000
26921472
18313.0000
6989071
18436.0000
5903583
18240.0000
2049952
19996.0000
1508581
22604.0020
1904953
28117.0020
1903799
29435.0020
1411118
mass

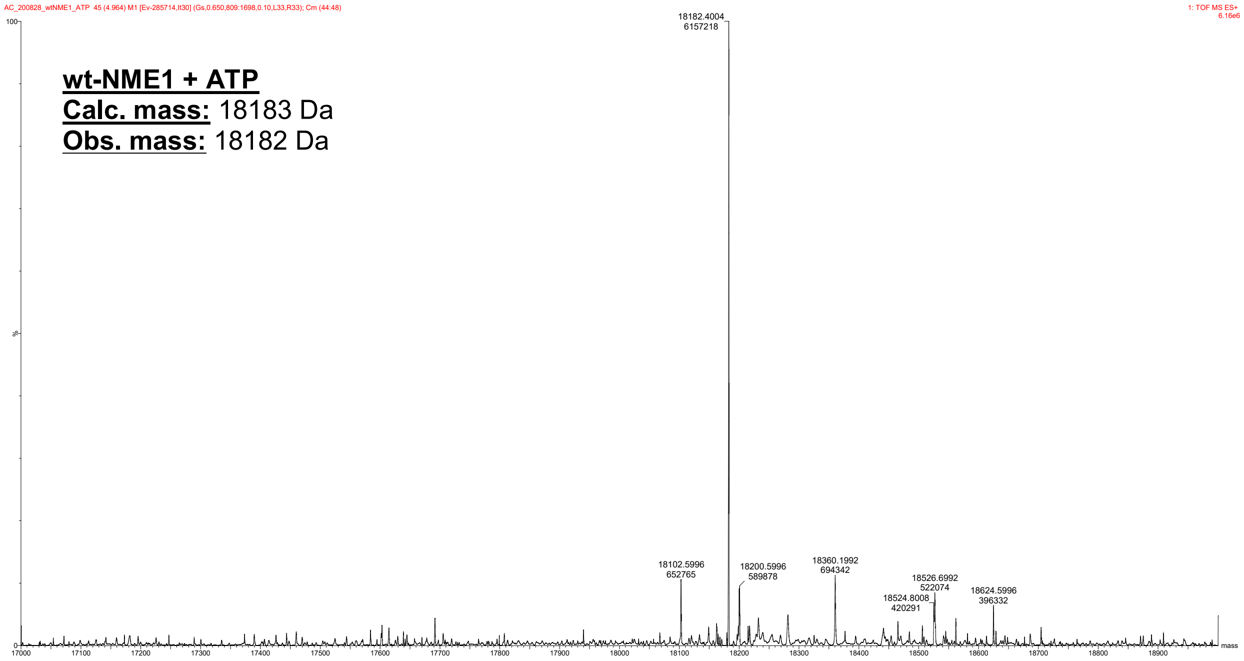

AC_200828_wtNME1_ATP 45 (4.964) M1 [Ev-285714,It30] (Gs,0.650,809:1698,0.10,L33,R33); Cm (44:48)
1: TOF MS ES+
6.16e6
wt-NME1 + ATP
Calc. mass: 18183 Da
Obs. mass: 18182 Da
18182.4004
6157218
18102.5996
652765
18200.5996
589878
18360.1992
694342
18526.6992
522074
18524.8008
420291
18624.5996
396332
100
%
0
17000
17100
17200
17300
17400
17500
17600
17700
17800
17900
18000
18100
18200
18300
18400
18500
18600
18700
18800
18900
mass

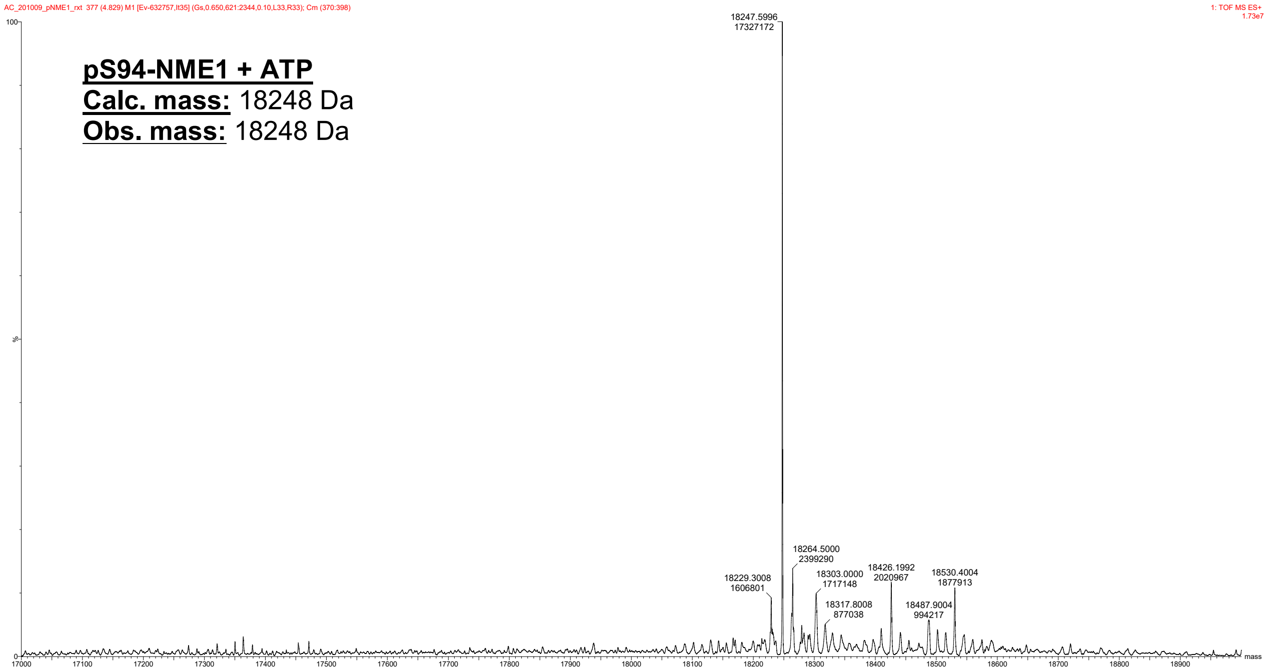
AC_201009_pNME1_rxt 377 (4.829) M1 [Ev-632757,It35] (Gs,0.650,621:2344,0.10,L33,R33); Cm (370:398)
1: TOF MS ES+
1.73e7
pS94-NME1 + ATP
Calc. mass: 18248 Da
Obs. mass: 18248 Da
18247.5996
17327172
18264.5000
2399290
18229.3008
1606801
18303.0000
1717148
18317.8008
877038
18426.1992
2020967
18487.9004
994217
18530.4004
1877913
100
%
0
17000
17100
17200
17300
17400
17500
17600
17700
17800
17900
18000
18100
18200
18300
18400
18500
18600
18700
18800
18900
mass

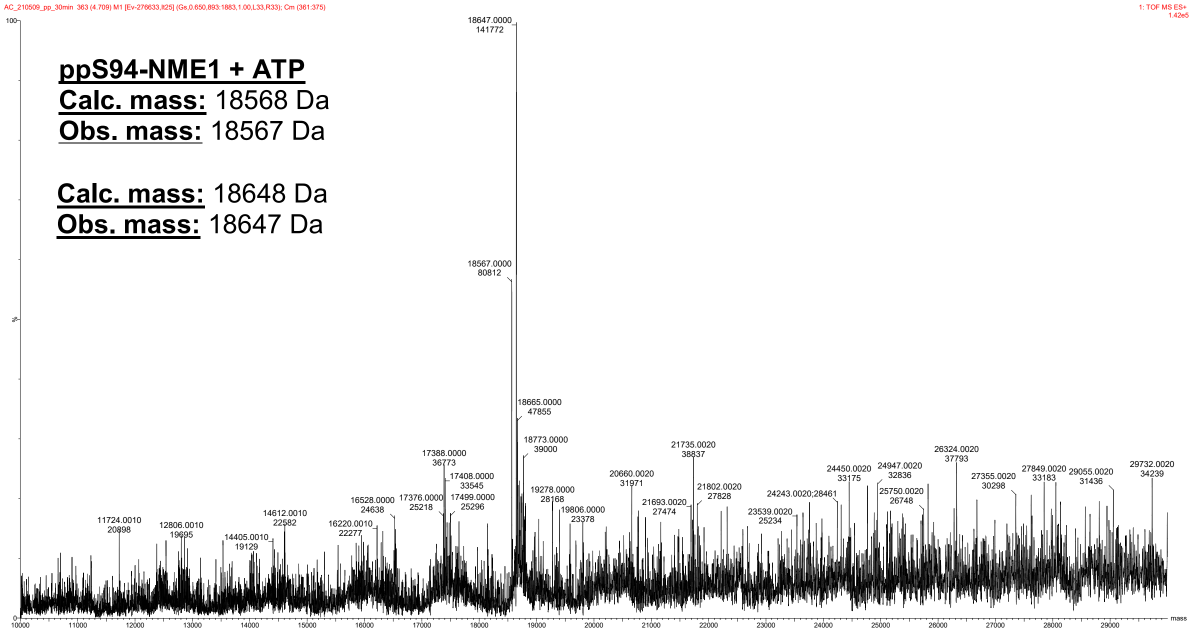

ppS94-NME1 + ATP
Calc. mass: 18568 Da
Obs. mass: 18567 Da
Calc. mass: 18648 Da
Obs. mass: 18647 Da
18647.0000
141772
18567.0000
80812

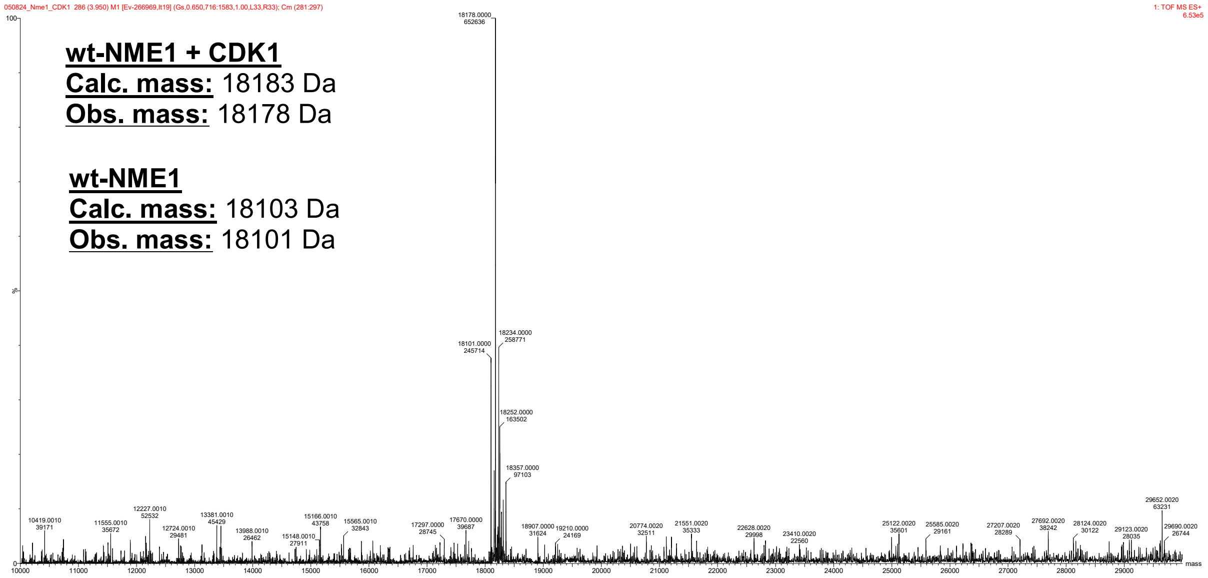
050824_Nme1_CDK1 286 (3.950) M1 [Ev-266969,It19] (Gs,0.650,716:1583,1.00,L33,R33); Cm (281:297)
1: TOF MS ES+
6.53e5
wt-NME1 + CDK1
Calc. mass: 18183 Da
Obs. mass: 18178 Da
wt-NME1
Calc. mass: 18103 Da
Obs. mass: 18101 Da
18178.0000
652636
18101.0000
245714
18234.0000
258771
18252.0000
163502
18357.0000
97103
%
mass

pT94-NME1
Calc.mass: 21012 Da
Obs.mass: 21012 Da

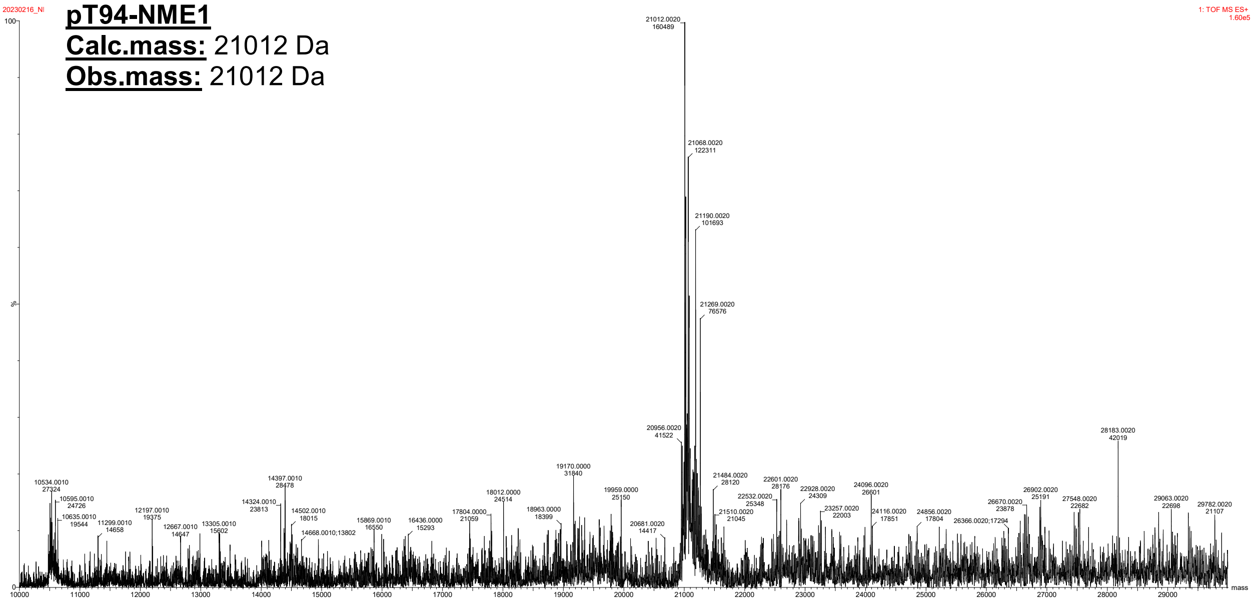

Sequence of pT94-NME1:

MHHHHHHGKPIPNPLLGLDSTENLYFQGIDPFTMANCERTFIAIKPDGVQRGLVGEIIKRFEQ
KGFRLVGLKFMQASEDLLKEHYVDLKDRPFFAGLVKYMHSGPVVAMVWEGLNVVKTGRVM
LGE[pT]NPADSKPGTIRGDFCIQVGRNIIHGSDSVESAEKEIGLWFHPEELVDYTSCAQNWIY
E

Unprocessed blots and gels for Supplementary Figures

SDS-PAGE: Recombinant expression of wt-NME1. Shown in **Supplementary Fig 1a.**

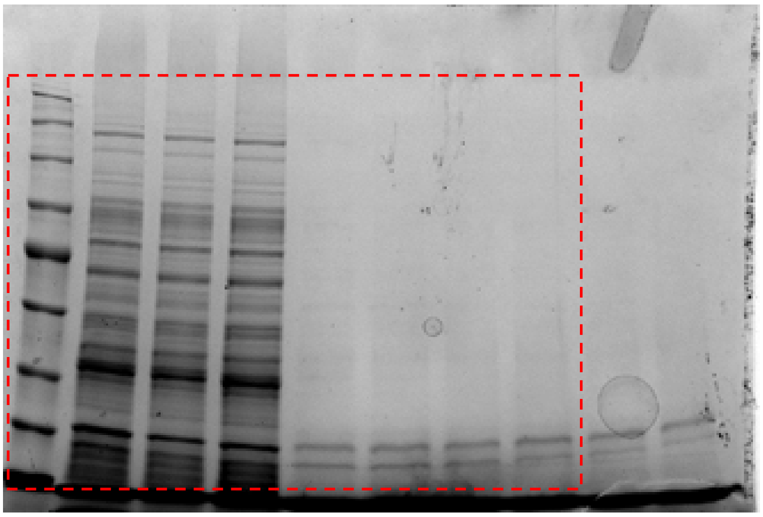

Anti-His_6 Western blot (merged image with ladder): Recombinant expression of pT94-NME1. Shown in **Supplementary Fig 2.**

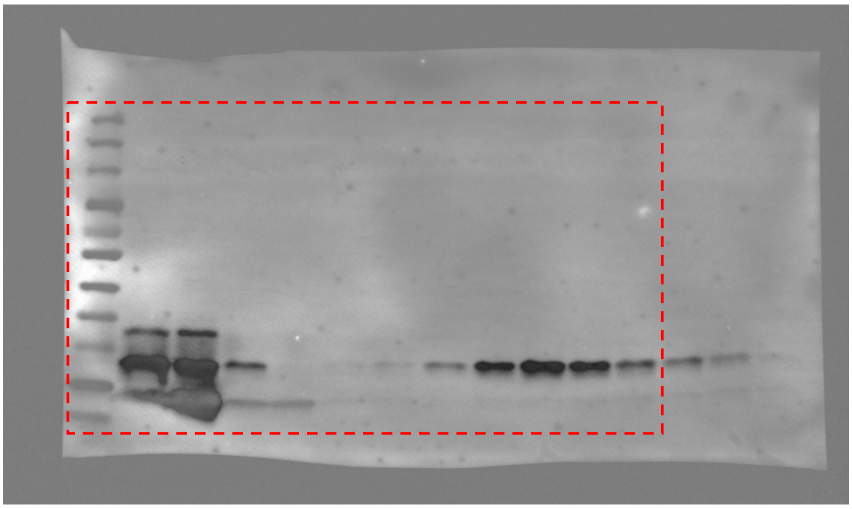

SDS-PAGE: Recombinant expression of pS94-NME1. Shown in **Supplementary Fig 3.**

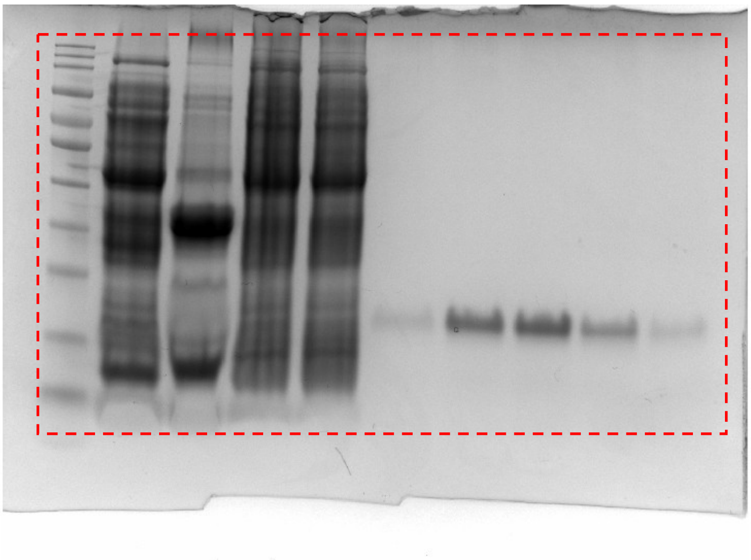

SDS-PAGE: Buffer screening for refolding of wt-NME1. Shown in **Supplementary Fig 4a.**

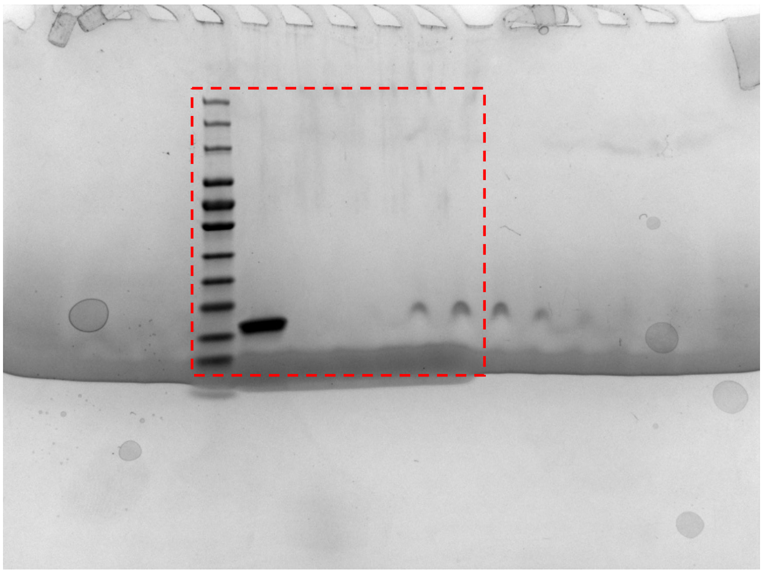

SDS-PAGE: Buffer screening for refolding of wt-NME1. Shown in **Supplementary Fig 4b.**

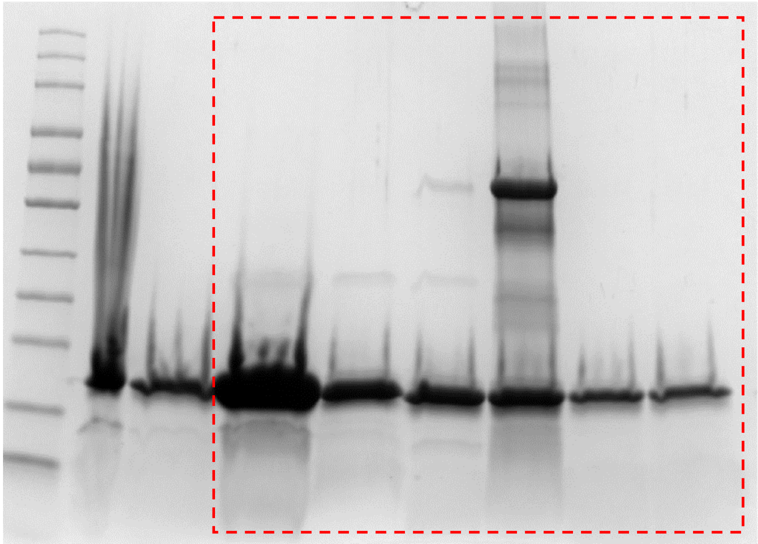

References

1 Morgan JAM, S. A., Kurz L, Nadler-Holly M, Ruwolt M, Ganguli S, Sharma S, Penkert M, Krause E, Liu F, Bhandari R, Fiedler D. Extensive protein pyrophosphorylation revealed in human cell lines. *Nat Chem Biol.* (2024).

2 Thingholm TE, J. O., Robinson PJ, Larsen MR. SIMAC (sequential elution from IMAC), a phosphoproteomics strategy for the rapid separation of monophosphorylated from multiply phosphorylated peptides. *Mol Cell Proteomics* **7**, 661-671 (2008).

3 Brendan MacLean, D. M. T., Nicholas Shulman, Matthew Chambers, Gregory L Finney, Barbara Frewen, Randall Kern, David L Tabb, Daniel C Liebler, Michael J MacCoss. Skyline: an open source document editor for creating and analyzing targeted proteomics experiments. *Bioinformatics* **26**, 966-068 (2010).

4 Kong AT, L. F., Avtonomov DM, Mellacheruvu D, Nesvizhskii AI. MSFragger: ultrafast and comprehensive peptide identification in mass spectrometry-based proteomics. *Nat Methods.* **14**, 513-520 (2017).

5 Punjani A, R. J., Fleet DJ, Brubaker MA. cryoSPARC: algorithms for rapid unsupervised cryo-EM structure determination. . *Nat Methods.* **14**, 290-296 (2017).

6 Punjani A, Z. H., Fleet DJ. Non-uniform refinement: adaptive regularization improves single-particle cryo-EM reconstruction. *Nat Methods.* **17**, 1214-1221 (2020).

7 Rubinstein JL, B. M. Alignment of cryo-EM movies of individual particles by optimization of image translations. *J Struct Biol.* **192**, 188-195 (2015).

8 Sanchez-Garcia, R. *et al.* DeepEMhancer: a deep learning solution for cryo-EM volume post-processing. *Commun. Biol.* **4**, 874 (2021). https://doi.org:10.1038/s42003-021-02399-1

9 Meng EC, G. T., Pettersen EF, Couch GS, Pearson ZJ, Morris JH, Ferrin TE. . UCSF ChimeraX: Tools for structure building and analysis. *Protein Sci.* **32**, e4792 (2023).

10 Emsley, P., Lohkamp, B., Scott, W. G. & Cowtan, K. Features and development of Coot. *Acta Crystallogr. D Biol. Crystallogr.* **66**, 486-501 (2010). https://doi.org:10.1107/s0907444910007493

11 TI., C. ISOLDE: a physically realistic environment for model building into low-resolution electron-density maps. *Acta Crystallogr D Struct Biol.* **74**, 519-530 (2018).

12 Afonine, P. V. *et al.* Real-space refinement in PHENIX for cryo-EM and crystallography. *Acta Crystallogr. D Struct. Biol.* **74**, 531-544 (2018). https://doi.org:10.1107/s2059798318006551

13 Moriarty NW, G.-K. R., Adams PD. electronic Ligand Builder and Optimization Workbench (eLBOW): a tool for ligand coordinate and restraint generation. *Acta Crystallogr D Biol Crystallogr.* **65**, 1074-1080 (2009).

14 Marmelstein AM, M. J., Penkert M, Rogerson DT, Chin JW, Krause E, Fiedler D. Pyrophosphorylation via selective phosphoprotein derivatization. *Chem Sci.* **9**, 5929-5936 (2018).

15 Qian K, H. B., Cummins C, Fiedler D. Monodisperse Chemical Oligophosphorylation of Peptides via Protected Oligophosphorimidazolide Reagents. *Angew Chem Int Ed Engl.* **64**, :e202419147 (2025).
