# Supplementary material for: Nucleoside diphosphate kinase A (NME1) catalyses its own oligophosphorylation
Source: Nat Chem. 2025 Aug 20;17(11):1757–67. doi: 10.1038/s41557-025-01915-8 (PMC12580328; doi:10.1038/s41557-025-01915-8)
Supplement: Supplementary file 2 — Reporting Summary [file 41557_2025_1915_MOESM2_ESM.pdf]

nature portfolio

Corresponding author(s): Dorothea Fiedler

Last updated by author(s): Jul 11, 2025

Reporting Summary

Nature Portfolio wishes to improve the reproducibility of the work that we publish. This form provides structure for consistency and transparency in reporting. For further information on Nature Portfolio policies, see our Editorial Policies and the Editorial Policy Checklist.

Statistics

For all statistical analyses, confirm that the following items are present in the figure legend, table legend, main text, or Methods section.

n/a	Confirmed	
☐	☒	The exact sample size (*n*) for each experimental group/condition, given as a discrete number and unit of measurement
☐	☒	A statement on whether measurements were taken from distinct samples or whether the same sample was measured repeatedly
☐	☒	The statistical test(s) used AND whether they are one- or two-sided<br>*Only common tests should be described solely by name; describe more complex techniques in the Methods section.*
☒	☐	A description of all covariates tested
☒	☐	A description of any assumptions or corrections, such as tests of normality and adjustment for multiple comparisons
☐	☒	A full description of the statistical parameters including central tendency (e.g. means) or other basic estimates (e.g. regression coefficient) AND variation (e.g. standard deviation) or associated estimates of uncertainty (e.g. confidence intervals)
☐	☒	For null hypothesis testing, the test statistic (e.g. *F*, *t*, *r*) with confidence intervals, effect sizes, degrees of freedom and *P* value noted<br>*Give P values as exact values whenever suitable.*
☒	☐	For Bayesian analysis, information on the choice of priors and Markov chain Monte Carlo settings
☒	☐	For hierarchical and complex designs, identification of the appropriate level for tests and full reporting of outcomes
☒	☐	Estimates of effect sizes (e.g. Cohen's *d*, Pearson's *r*), indicating how they were calculated

Our web collection on statistics for biologists contains articles on many of the points above.

Software and code

Policy information about availability of computer code

Data collection	High-resolution ESI-MS spectra were recorded on two different instruments: Agilent 6220 TOF Accurate Mass coupled to an Agilent 1200 LC (Agilent Technologies, USA) was used to obtain HRMS of purified protein. LC-MS/MS analysis were performed using an UltiMate 3000 RSLC nano-LC system coupled on-line to an Orbitrap Fusion mass spectrometer operated using Xcalibur software package version 3.4 or coupled on-line to an Orbitrap Fusion Lumos mass spectrometer operated using Xcalibur software package version 4.1. Cryo-EM data were acquired on a TFS Titan Krios G3i TEM equipped with a Gatan Bioquantum K3 detector with energy filter. EPU version 2.12 was used for data collection. Vitrification of proteins was performed on Quantifoil 1.2/1.3 Cu 300 mesh grids using a Vitrobot Mark IV. Side-directed mutagenesis was performed on a BIO-RAD ClO00 Touch TM Thermal Cycler. BIO-RAD NGCTM chromatography system with a BioFrac Fraction Collector was used to purified all recombinant proteins. NANODROP 2000C spectrophotometer (Thermo Fisher Scientific) was used to determine DNA and protein concentration. Luminescence assays were read out with Tecan Infinite M Plex reader.
Data analysis	Cryo-EM data were processed in CryoSPARC version 4.4.1. Atomic models were refined using Phenix versions 1.20.1-4487 (NME1-1P) and 1.21-5207 (NME1-2P and NME1-3P), respectively. MS raw data was analyzed using Freestyle (Thermo Fisher Scientific) version 1.7.73.12, Molecular Weight Calculator (Matthew Monroe https:// alchemistmatt.com/resume/mwtoverview.html) version 6.50, MaxQuant version 2.0.3.0, Perseus version 1.6.7.0, Skyline version 24.1.0, and FragPipe v21/v22 (MSFragger version 4.0, Philosopher v5.1.0/v5.1.1). Proteomics data analysis and visualization were conducted using R (RStudio v2024.04.2). Statistical analyzed and graph preparation were done using GraphPad Prism v5. Gene ontology term analysis was done using Enrichr.

For manuscripts utilizing custom algorithms or software that are central to the research but not yet described in published literature, software must be made available to editors and reviewers. We strongly encourage code deposition in a community repository (e.g. GitHub). See the Nature Portfolio guidelines for submitting code & software for further information.

Data

Policy information about availability of data

All manuscripts must include a data availability statement. This statement should provide the following information, where applicable:

- Accession codes, unique identifiers, or web links for publicly available datasets
- A description of any restrictions on data availability
- For clinical datasets or third party data, please ensure that the statement adheres to our policy

The mass spectrometry proteomics data have been deposited to the ProteomeXchange Consortium via the PRIDE partner repository with the dataset identifier PXD054175.82 The corresponding token for accession is Pv7yHEDOIr2D.Database for the human proteome: uniprot (https://www.uniprot.org/uniprotkb?query=Human). Database for NME1 phosphorylation sites: PhosphoSitePlus (https://www.phosphosite.org/-proteinAction.action?id=3836&showAllSites=true). The cryo-EM densities and resulting structural models of pS94-NME1, ppS94-NME1, and oligo-pS94-NME1 have been deposited in the EMDB and PDB under accession numbers 51248 and 9GD6, 51250 and 9GD8, 51251 and 9GD9, respectively. Source data are provided with this paper.

Research involving human participants, their data, or biological material

Policy information about studies with human participants or human data. See also policy information about sex, gender (identity/presentation), and sexual orientation and race, ethnicity and racism.

Reporting on sex and gender	N/A
Reporting on race, ethnicity, or other socially relevant groupings	N/A
Population characteristics	N/A
Recruitment	N/A
Ethics oversight	N/A

Note that full information on the approval of the study protocol must also be provided in the manuscript.

Field-specific reporting

Please select the one below that is the best fit for your research. If you are not sure, read the appropriate sections before making your selection.

☒ Life sciences ☐ Behavioural & social sciences ☐ Ecological, evolutionary & environmental sciences

For a reference copy of the document with all sections, see nature.com/documents/nr-reporting-summary-flat.pdf

Life sciences study design

All studies must disclose on these points even when the disclosure is negative.

Sample size	Sample sizes were chosen based on prior extensive experience on the assays performed in this study (Fuhs et al., Cell 2015; Morgan et al., Nat. Chem. Biol. 2024; Celik et al., J. Am. Chem. Soc. 2024; Cheng et al., Curr. Opin. Struct. Biol. 2024; Bekker-Jensen et al., Cell Syst. 2017).
Data exclusions	No data was excluded from the analysis.
Replication	Experiments were conducted in independent biological replicates. The number of replicates is indicated in the respective figure legend. All attempts at replication were successful.
Randomization	In cell-based experiments including western blotting, interactomics, Cryo-EM, and mass spectrometry, randomization is not possible or applicable.
Blinding	Western blot analysis was not blinded as the gel loading order needs to be defined. For MS experiments, blinding is not applicable.

Reporting for specific materials, systems and methods

We require information from authors about some types of materials, experimental systems and methods used in many studies. Here, indicate whether each material, system or method listed is relevant to your study. If you are not sure if a list item applies to your research, read the appropriate section before selecting a response.

Materials & experimental systems

n/a	Involved in the study
☐	☒ Antibodies
☐	☒ Eukaryotic cell lines
☒	☐ Palaeontology and archaeology
☒	☐ Animals and other organisms
☒	☐ Clinical data
☒	☐ Dual use research of concern
☒	☐ Plants

Methods

n/a	Involved in the study
☒	☐ ChIP-seq
☒	☐ Flow cytometry
☒	☐ MRI-based neuroimaging

Antibodies

Antibodies used	Antibodies used in this study for western blot analysis were: Anti-Nl-Phosphohistidine (1-pHis) Antibody, clone SC50-3 (Sigma-Aldrich, MABS1341, dilution 1:1000); Anti-NMEl Antibody (Cell Signaling Technology, #3345, dilution 1:1000); Anti-rabbit IgG, HRP-linked Antibody (Cell Signaling Technology, #7074, dilution 1:1000).
Validation	As per the manufacturer's website, Anti-Nl-Phosphohistidine (Sigma-Aldrich, MABS1341) was validated by WB using recombinant human NME1 (NM23-H1) autophosphorylation reaction (5 µg), and Anti-NMEl Antibody (Cell Signaling Technology, #3345) was validated in extracts from various cell lines by WB and IHC.

Eukaryotic cell lines

Policy information about cell lines and Sex and Gender in Research

Cell line source(s)	HEK293T cells were from ATCC, CRL-3216.
Authentication	HEK293T cells were authenticated using STR profiling. STR profiling was performed following ISO 9001 and ISO/IEC 17025 quality standards.
Mycoplasma contamination	Cell line tested negative for Myocoplasma contamination.
Commonly misidentified lines (See ICLAC register)	Not listed as commonly misidentified lines.

Plants

Seed stocks	N/A
Novel plant genotypes	N/A
Authentication	N/A
