# Supplementary figures and images for: Nucleoside diphosphate kinase A (NME1) catalyses its own oligophosphorylation
Source: Nat Chem. 2025 Aug 20;17(11):1757–67. doi: 10.1038/s41557-025-01915-8 (PMC12580328; doi:10.1038/s41557-025-01915-8)

## Uncropped blots for Figure 2c

anti-NME1

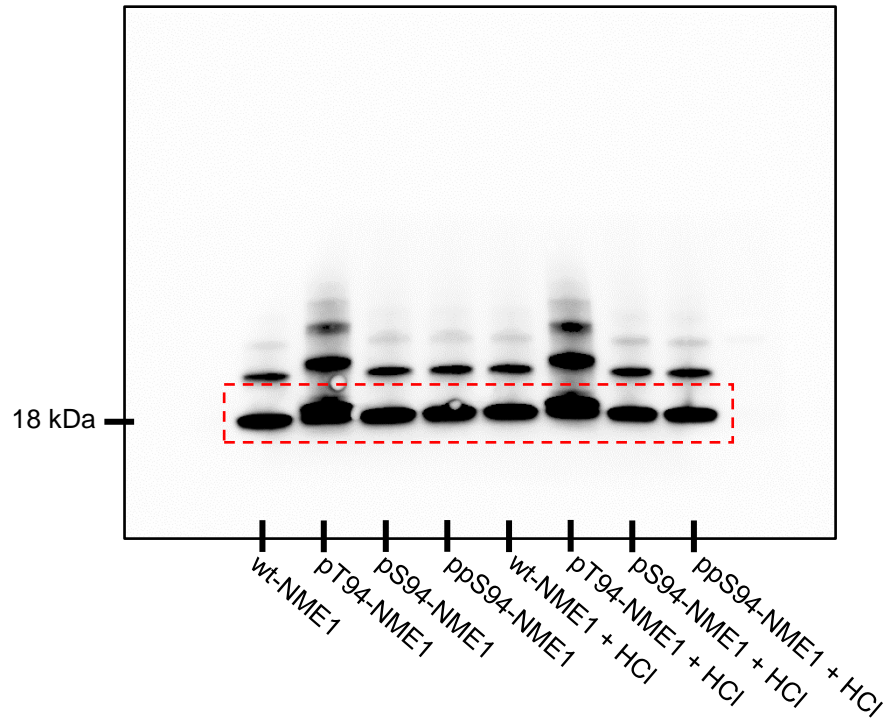

anti-pHis

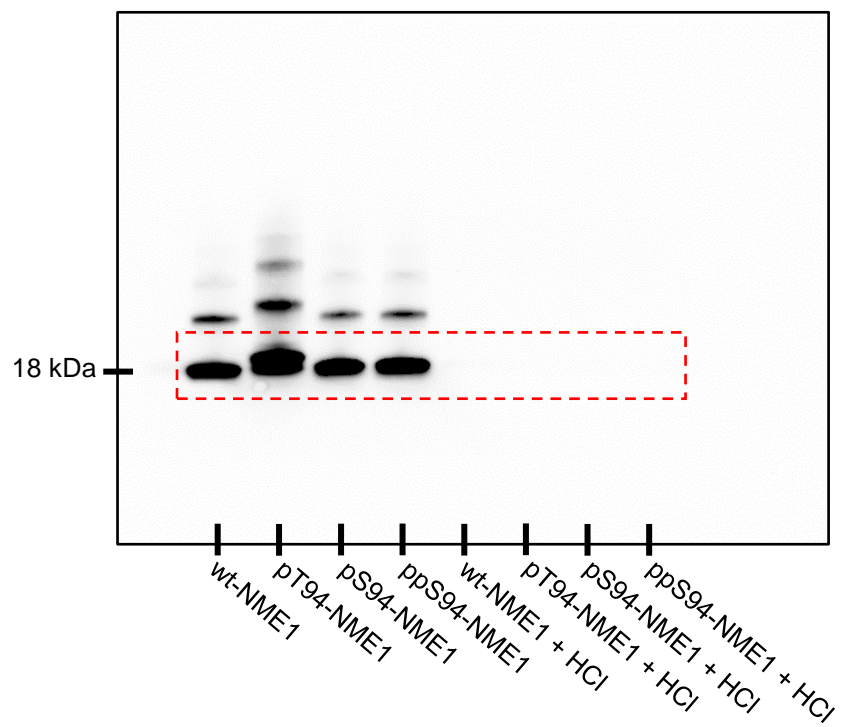

Supplement: Supplementary file 5 — Unprocessed western blots [file 41557_2025_1915_MOESM5_ESM.pdf]
